# Bioadhesive‐Integrated 3D‐Printed Multilayer Scaffolds Secure a Mechanically‐Stable Osteoimmune Niche for Enhanced Bone Augmentation

**DOI:** 10.1002/advs.77028

**Published:** 2026-08-11

**Authors:** Delu Zhao, Qiuyi Li, Lingyun Wang, Chongao Zhang, Yutian Cheng, Shuang Liu, Fulan Wei, Zheqin Dong

**Affiliations:** ^1^ Department of Orthodontics & Additive Manufacturing, School and Hospital of Stomatology, Cheeloo College of Medicine Shandong University & Shandong Key Laboratory of Oral Diseases & Shandong Engineering Research Center of Dental Materials and Oral Tissue Regeneration & Shandong Provincial Clinical Research Center for Oral Diseases Jinan Shandong China

**Keywords:** 3D printing, adhesive fixation, bone regeneration, mechanical stability, mechanobiology

## Abstract

Early mechanical stabilization within the osteogenic niche is a key regulator of bone regeneration fate, and maintaining stability in material‐driven repair remains a clinical challenge in dynamic, non‐retentive, and anatomically complex bone defects, where implant materials are difficult to achieve stable fixation. However, most existing repair systems lack satisfactory strategies for addressing it. Here, this study reports an interfacial‐fixation–centered scaffold design realized by a bioadhesive‐integrated multilayer architecture fabricated via 3D‐printing, comprising a soft‐tissue–blocking barrier layer, a wet‐adhesive interface, and a porous osteogenic framework. The scaffold exhibits rapid and robust adhesion to bone under blood‐rich physiological conditions, enabling immediate stabilization of the osteogenic niche. In rabbit mandibular and tibial augmentation models, it significantly outperforms non‐adhesive scaffolds and the specific guided‐bone‐regeneration‐like comparator. Mechanistically, adhesive‐mediated stabilization reduced scaffold micromotion and was associated with a pro‐regenerative mechano‐immune profile, including increased M2‐like macrophage polarization and enhanced VEGF/BMP2‐associated angiogenic–osteogenic coupling. By highlighting the interface‐fixation‐mediated mechanical stabilization of the osteogenic niche, this work offers a new framework for enhancing bone augmentation in dynamic and complex defect settings, provides insights into potential mechano‐immune cues for mechanical‐stability‐secured bone augmentation, and demonstrates the scaffold's potential in bone repair.

## Graphical abstract

A 3D‑printed multilayer adhesive scaffold, firmly anchored to bone defects by simple finger pressing, suppresses micromotion and secures mechanical stability of the osteogenic niche. This stabilization likely contributes to the enhanced bone augmentation by fostering a pro‑regenerative mechano‐immune profile—characterized by M2‑like macrophage polarization and enhanced VEGF/BMP2‑mediated angiogenic–osteogenic coupling.

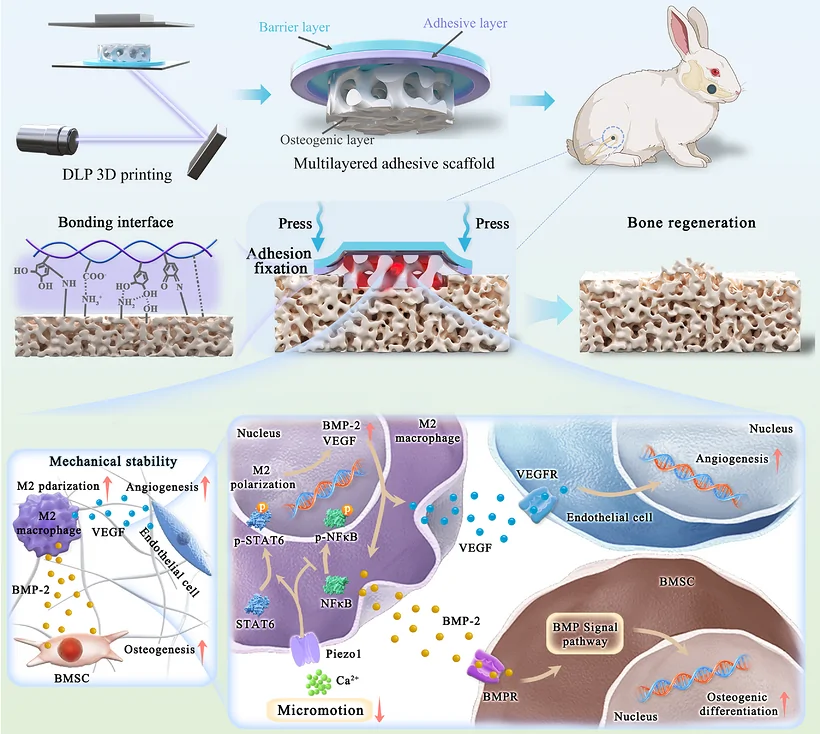

## 1 Introduction

The repair of bone defects, particularly those in dynamic, non‐retentive, or anatomically complex defects, remains a major clinical conundrum. For these defects, in the scaffold design of scaffold‐mediated regenerative therapies that possess clinical translation potential, beyond the regulation of biochemical signals for bone regeneration, mechanical stability mediated by scaffold‐bone interfacial fixation is also a critical core factor. Because bone regeneration is governed by the establishment of a mechanically stable and biologically permissive osteogenic niche during the early healing phase. Mechanical stability is not merely a structural prerequisite but an active regulator of blood clot retention, immune cell recruitment and phenotypization, angiogenesis, and osteogenic lineage commitment [1, 2, 3, 4, 5]. Conversely, insufficient stabilization induces micromotion or displacement at the tissue interface, disrupting early repair cascades and frequently leading to fibrous tissue formation [6], delayed union, or nonunion [7, 8], and even bone regeneration failure [6, 9]. Importantly, the mechanical stability of the osteogenic zone dictates the nature of local mechanical stimuli, thereby shaping distinct early mechano‐immunological microenvironments that regulate subsequent immune–angiogenic–osteogenic cascade events [10, 11, 12, 13]. These findings indicate that mechanical stability within the osteogenic zone is critical for successful bone regeneration. Nevertheless, achieving stable fixation of the implanted scaffold and orchestrating the involved cascade remain a major challenge in these bone defects.

Typically, maxillomandibular defect repair often exemplifies these challenges, as it faces complex and unfavorably retentive defects combined with persistent dynamic loading from mastication and facial movement, hindering the maintenance of early interfacial‐fixation stability for implanted scaffolds, thus compromising the mechanical stability of the osteogenic niche (local cellular and tissue microenvironment within the bone augmentation region reconstructed by the engineered scaffold) [14, 15, 16]. Clinically, guided bone regeneration (GBR) represents the predominant clinical approach to address these challenges through bone grafts, barrier membranes, and fixation hardware intended to preserve space and immobilize grafted materials. While widely adopted, this strategy remains limited by poor shape fidelity of particulate grafts, susceptibility of membranes and grafts to displacement, and reliance on metallic fixation devices that increase surgical complexity, patient burden, and the risk of secondary trauma [14, 17]. Collectively, these shortcomings underscore a critical unmet need: bone repair systems that intrinsically establish early interfacial mechanical stability without external hardware.

In this context, additive manufacturing provides a powerful platform for fabricating patient‐specific bone tissue engineering (BTE) scaffolds with precisely defined architectures and spatially organized functionalities, enabling high shape fidelity and functional integration [18, 19, 20, 21]. Leveraging this design freedom, substantial progress has been made in developing porous scaffolds or multilayer scaffolds that promote osteogenesis and vascularization while preventing soft‐tissue infiltration [18, 22]. However, most 3D‐printed scaffolds remain primarily engineered with respect to bulk architecture and biochemical cues, whereas the dynamic mechanical state at the scaffold–tissue interface—the locus where early biological decisions are initiated—has received comparatively little direct design attention. Consequently, intrinsic interfacial stabilization capability is still largely absent from current scaffold designs, despite its well‐established importance for successful bone regeneration.

Bioadhesives offer a compelling materials‐based solution to this challenge. Modern bioadhesives form robust bonds to wet tissues through covalent and noncovalent interactions [23, 24] and have been applied in wound closure [25], gastrointestinal sealing [26], neural electrode fixation [27], and bone bonding [28, 29, 30]. Adhesive‐functionalized GBR membranes further demonstrate improved positional stability and enhanced regeneration [14, 31, 32]. These studies indicate that integrating adhesive gels into BTE scaffolds to provide adhesive fixation and ensure mechanical stability in the osteogenic niche holds great promise. However, how to integrate adhesive functionality into scaffolds remains unclear. Therefore, it is necessary to rationally design scaffolds that simultaneously possess osteogenic activity and strong adhesive fixation as intrinsic properties.

Here, we hypothesize that embedding intrinsic interfacial fixation directly into scaffold architecture suppresses early micromotion, establishes a mechanically stabilized osteogenic niche, and reprograms mechano‐immune signaling to coordinate angiogenic–osteogenic coupling and bone regeneration. Guided by this concept, we propose a novel scaffold design concept for BTE that integrates adhesive fixation functionality into the scaffold construct, enabling immediate fixation to bone defects to ensure early‐stage mechanical stability of the bone regeneration niche and promote osteogenesis. Specifically, we designed and fabricated a multilayered adhesive scaffold (ML‑AD) via 3D printing, which integrates three functional layers: (i) a soft‐tissue‐blocking barrier layer serving as the connection structure between the adhesive and the osteogenic layer; (ii) a wet‐adhesive interlayer enabling rapid fixation under blood‐rich conditions; (iii) a patient‐specific porous osteogenic layer (Scheme 1). Using rabbit mandibular and tibial augmentation models, we demonstrate that ML‐AD achieves immediate fixation without external hardware and significantly outperforms non‐adhesive scaffolds and the specific GBR‐like comparator used in this rabbit augmentation model. Mechanistically, adhesive‐mediated stabilization improving bone augmentation is correlated with suppressed scaffold micromotion and the consequent favorable mechano‐immune profile characterized by enhanced M2‐like macrophage polarization, increased VEGF/BMP2‐associated angiogenic–osteogenic coupling. By elevating the mechanical stabilization to a central scaffold design principle, this work will establish a new framework for bone augmentation in dynamic and complex defect settings, and demonstrates the scaffold's potential in bone repair.

**SCHEME 1:**
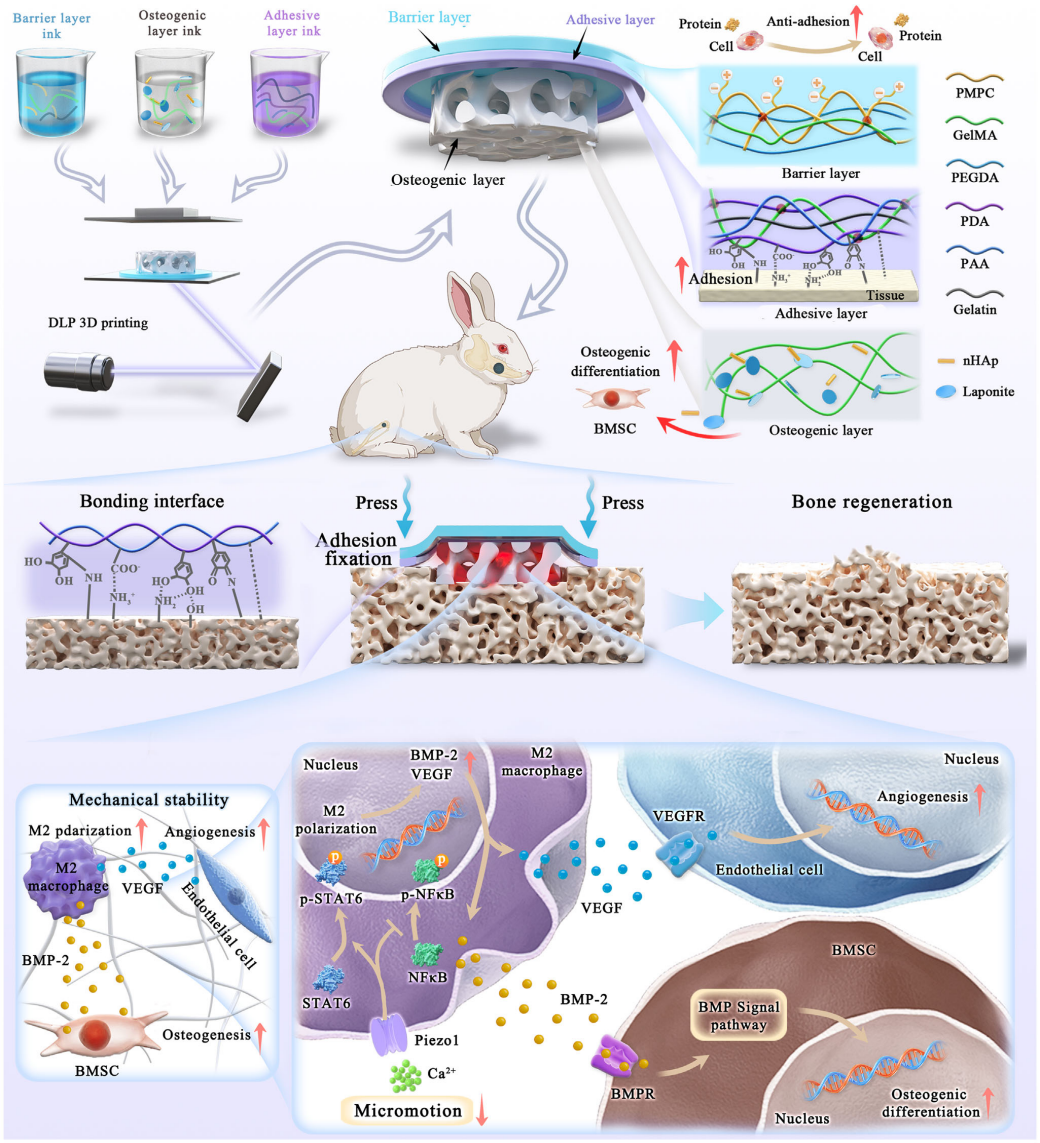
Schematic illustration of the design, fabrication, and application of a multi‐layer adhesive scaffold (ML‐AD) for bone augmentation. The scaffold comprises three layers: a barrier layer (preventing soft tissue detrimental to osteogenesis from invading the osteogenic zone), an adhesive layer (providing adhesive fixation), and an osteogenic layer (patient‐specific osteogenic porous architecture). The scaffold achieves instantaneous bonding to bone defects through pressing, ensuring mechanical stability in the osteogenic zone. By reducing micromotion in the region, it promoted M2‐like macrophages polarization, thereby enhanced VEGF‐ and BMP2‐mediated angiogenic and osteogenic signaling in the osteogenic region, ultimately facilitating bone augmentation.

## 2 Results

### 2.1 Optimization of Formulations for Each Layer of ML‐AD and Corresponding Characterization

To construct a multilayer adhesive scaffold with integrated barrier, adhesive, and osteogenic functions, the formulations of each layer were systematically optimized, and the corresponding characterizations were performed, including anti‐adhesion performance, adhesive performance, osteogenic differentiation‐promoting capacity, and the corresponding physicochemical property characterizations.

The barrier layer, analogous to the membrane used in GBR, is designed to resist protein adsorption and prevent soft‐tissue infiltration (e.g., fibroblasts), thereby preserving space for osteogenesis‐related cell colonization [33]. The barrier layer hydrogel formulation comprises GelMA and PEGDA to provide mechanical integrity, with varying concentrations of methacryloyloxyethyl phosphorylcholine (MPC)—a zwitterionic monomer with strong hydration‐mediated anti‐fouling properties—incorporated to suppress protein and cell adhesion (Figure 1A) [34]. The mixture is subsequently photopolymerized to form the barrier layer hydrogel (the chemical structure of the barrier layer is shown in Figure 1A; based on MPC contents of 0, 10%, 20%, and 30%, the barrier layer hydrogels were designated as M0, M10, M20, and M30, respectively; the detailed grouping and formulation compositions are shown in Table S1). All groups exhibited excellent cytocompatibility (Figure S2). As expected, increasing MPC content progressively reduced protein adsorption (BSA and FBS) (Figure 1B,C; Figure S3A–C), reaching saturation at 20% MPC. A similar trend was observed for cell adhesion, as both rat gingival fibroblasts (R‐GFs) and BMSCs showed markedly reduced attachment on M20 hydrogels (Figure 1B,D; Figures S3D,S4). Subsequently, characterization of the hydrogel of the barrier layer (M20) was conducted. Mechanical property tests showed that the compressive and tensile moduli of M20 were significantly higher than those of M0 (*P* < 0.05), indicating that the addition of MPC significantly improved its mechanical properties (Figure S5). Fourier‐transform infrared (FTIR) spectra confirmed successful MPC incorporation, with M20 hydrogel exhibiting a new peak at 783 cm^−^^1^ and enhanced peaks at 954 and 1725 cm^−^^1^ compared with M0 and poly MPC (PMPC, a single‐component MPC, was used as a control for comparison) (Figure 1L). Scanning electron microscope (SEM) test revealed that the freeze‐dried M20 hydrogel exhibited a uniformly porous structure throughout its interior, with pore sizes (10–30 µm), and EDS elemental analysis detected phosphorus (P) in M20 but not in M0 (as a control), indicating successful incorporation of MPC into the hydrogel (Figure S6). In addition, M20 hydrogels demonstrated dimensional stability with a modest swelling ratio of ∼7, and an appropriate degradation profile to sustain long‐term barrier function (Figure S7). Based on these evaluations, a 20% MPC formulation was selected for fabricating the barrier layer of the ML‐AD scaffold.

**FIGURE 1:**
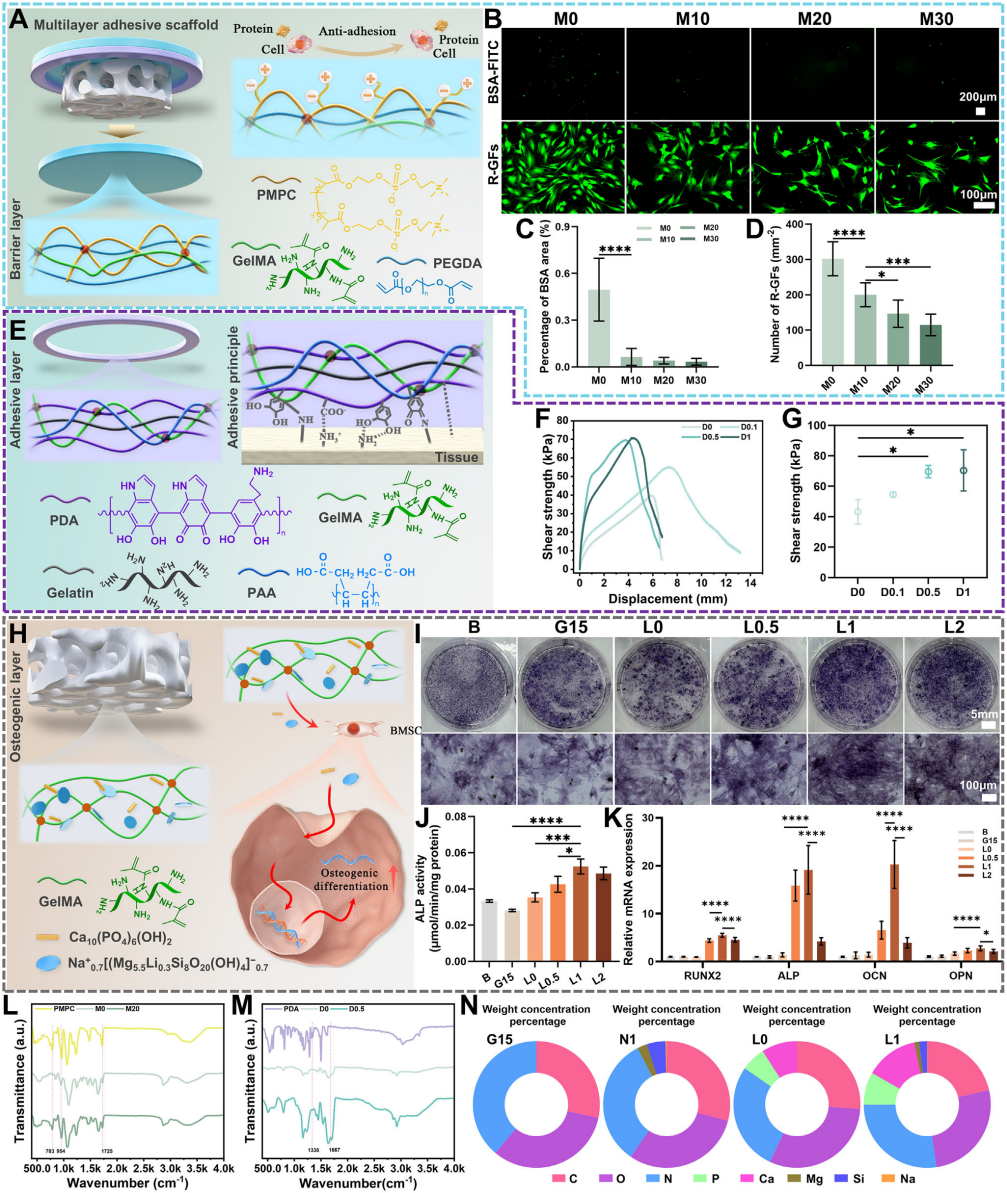
Formulation optimization of each layer (barrier layer, adhesive layer, and osteogenic layer) of the ML‐AD scaffold is based on specific functions and corresponding characterization. (A) Schematic illustration of the chemical composition and anti‐adhesion property of the barrier layer; based on MPC contents of 0%, 10%, 20% and 30%, unoptimized barrier layer hydrogels were denoted as M0, M10, M20, and M30, respectively (listed in Table S1). (B–D) Representative images of BSA‐FITC and R‐GFs (Calcein‐AM/PI) adhered to hydrogels with different formulations of the barrier layer (B), along with quantitative analysis of corresponding protein adhesion (C) and R‐GFs adhesion (D) (*N* = 3). (E) Schematic of chemical composition and adhesion function of the adhesive layer; unoptimized adhesive layer hydrogels were named D0, D0.1, D0.5, and D1 according to the DA contents of 0%, 0.1%, 0.5%, and 1%, respectively (listed in Table S2). (F,G) Representative displacement–shear strength curves (F) and bone adhesion shear strength (G) for bone adhesion shear strength tests (in the presence of PBS) of hydrogels with different formulations in the adhesive layer (*N* = 3). (H) Schematic diagram of the chemical composition and osteogenic differentiation‐promoting function of the osteogenic layer; unoptimized osteogenic layer hydrogels were divided into G15, L0, L0.5, L1, and L2 according to the contents of nHAp (0% and 6%) and Laponite (0%, 0.5%, 1%, and 2%) (detailed in Table S3). (I,J) Representative ALP staining images (I) and corresponding quantitative ALP activity assays (J) after 7 days of BMSC culture with hydrogels of different formulations in the osteogenic layer (*N* = 3). (K) Quantitative analysis of osteogenesis‐related gene expression in BMSCs determined by RT‐qPCR after 7 days of co‐culture with hydrogels from each osteogenic layer formulation (*N* = 3). (L,M) FTIR spectra of the optimized barrier layer (M20) and its components (L), as well as the optimized adhesive layer (D0.5) and its components (M). (N) EDS elemental weight percentages of the optimized osteogenic layer (L1) and the control group (G15, L0, and N1(comprising only 1% Laponite and 15% GelMA)). Statistical analysis in this figure was performed using one‑way ANOVA. *p < 0.05; ***p < 0.001; ****p < 0.0001.

The adhesive layer, a core component of the scaffold, is designed to wet‐bond the construct to bone surfaces in vivo, thereby providing immediate mechanical fixation and stability. To achieve strong wet adhesion, a hydrogel formulation composed of acrylic acid (AA), GelMA, gelatin, and dopamine (DA) was developed, in which GelMA serves as the crosslinkable backbone, gelatin enhances biocompatibility, and AA and DA function as the primary adhesive moieties (Figure 1E). This mixture is photopolymerized to form the adhesive hydrogels (adhesive hydrogels were named D0, D0.1, D0.5, and D1 according to the DA contents of 0%, 0.1%, 0.5%, and 1%, respectively; detailed information regarding their grouping and formulations is shown in Table S2). Because the adhesive performance depends strongly on the density of polydopamine (PDA) generated from DA oxidation and on the crosslinking degree of the network [35, 36, 37], both DA concentration and UV exposure time were optimized. Adhesion testing revealed that shear strength increased with UV illumination time, reached a maximum at 150 s, and then declined with further exposure (Figure S8). Increasing DA concentration significantly enhanced adhesive strength, reaching a plateau at 0.5% DA (D0.5) (Figure 1F,G). The adhesive hydrogels also exhibited excellent cytocompatibility, as demonstrated by cell viability and proliferation assays (Figure S9). Therefore, a DA concentration of 0.5% and a UV exposure time of 150 s were selected as the optimal parameters for fabricating the adhesive layer in the scaffold. Then, the physicochemical properties of the adhesive hydrogel (D0.5) were further characterized. In the FTIR analysis, compared to D0, D0.5 exhibits a new peak at 1338 cm^−^^1^ and an enhanced peak at 1667 cm^−^^1^a new peak, which, when cross‐referenced with the PDA spectrum, confirmed the successful incorporation of DA (Figure 1M). SEM characterization and EDS results revealed that D0.5 exhibits a dense, non‐porous structure, and the incorporation of DA into D0 was successfully achieved (Figure S10). Additionally, the adhesive layer (D0.5) exhibited good dimensional stability and an appropriate degradation profile conducive to bone adhesive (Figure S11). Thus, these further indicate that a 0.5% DA concentration is appropriate for the adhesive layer.

The osteogenic layer was formulated using GelMA to ensure biocompatibility and 3D printability, nano‐hydroxyapatite (nHAp) to mimic bone mineral, and Laponite (Na^+^_0_._7_[(Mg_5_._5_Li_0_._3_Si_8_O_20_(OH)_4_]^−^_0_._7_) to promote osteogenic differentiation and enhance bone‐forming capacity (osteogenic layer hydrogels were divided into G15, L0, L0.5, L1, and L2 according to the contents of nHAp (0% and 6%) and Laponite (0%, 0.5%, 1%, and 2%); the specific grouping and formulations are detailed in Table S3; the chemical structure and mechanism diagram of promoting osteogenic differentiation of BMSCs shown in Figure 1H) [38]. First, cytocompatibility tests demonstrated excellent BMSC viability and proliferation of BMSCs across all groups (Figure S12). Osteogenic evaluation further showed that alkaline phosphatase (ALP) activity increased with rising Laponite content and peaked in the L1 group, as evidenced by ALP staining and quantitative ALP assays (Figure 1I,J). Von Kossa and alizarin red S (ARS) staining exhibited the same trend (Figure S13). Consistently, expression levels of osteogenesis‐related genes (RUNX2, ALP, OCN, and OPN) were highest in the L1 group (Figure 1K). Thus, these results identify the L1 formulation as optimal for constructing the osteogenic layer in the scaffold. Subsequently, the osteogenic layer hydrogel (L1) was prepared and subjected to physicochemical characterization. SEM and EDS analyses confirmed the uniform distribution of Laponite and nHAp throughout the hydrogel, with representative elemental signals (P, Ca, Mg, and Si) clearly detected in L1 (Figure 1N; Figure S14). Because the osteogenic layer serves as a structural compartment for bone‐regeneration‐related cell infiltration and bone formation, its mechanical properties are critical [39]. Mechanical testing revealed that incorporating nHAp and Laponite into GelMA (G15) substantially enhanced both compressive (93.93 ± 2.50 kPa) and tensile modulus (88.58 ± 6.50 kPa) in the L1 formulation compared with other control groups (Figure S15), ensuring sufficient mechanical performance for tissue regeneration. Swelling and degradation assessments further indicated that L1 exhibited a moderate swelling ratio and an appropriate degradation rate compatible with bone healing timelines (Figure S16). Hence, these results further suggest that the L1 formulation is appropriate.

### 2.2 Fabrication and Characterization of ML‐AD

Having optimized the formulations of the individual layers, the multilayer adhesive scaffold consisting of a film‐like barrier layer, a ring‐shaped adhesive layer, and a porous osteogenic layer was next fabricated (Figure 2A). The porous compartment adopted a triply periodic minimal surface (TPMS) Split‐P architecture, a geometry widely used in BTE scaffolds for its interconnected porosity and mechanical robustness (Figure S17) [40]. Digital light processing (DLP) printing was selected for fabricating the multilayer scaffold due to its high resolution and design flexibility. Photorheology measurements indicated that all ink formulations exhibited a rapid sol–gel transition (<20 s), ensuring their compatibility with DLP printing (Figure S18). Following optimization of printing parameters for each formulation (Figure S19), the multilayer adhesive scaffold was successfully fabricated.

**FIGURE 2:**
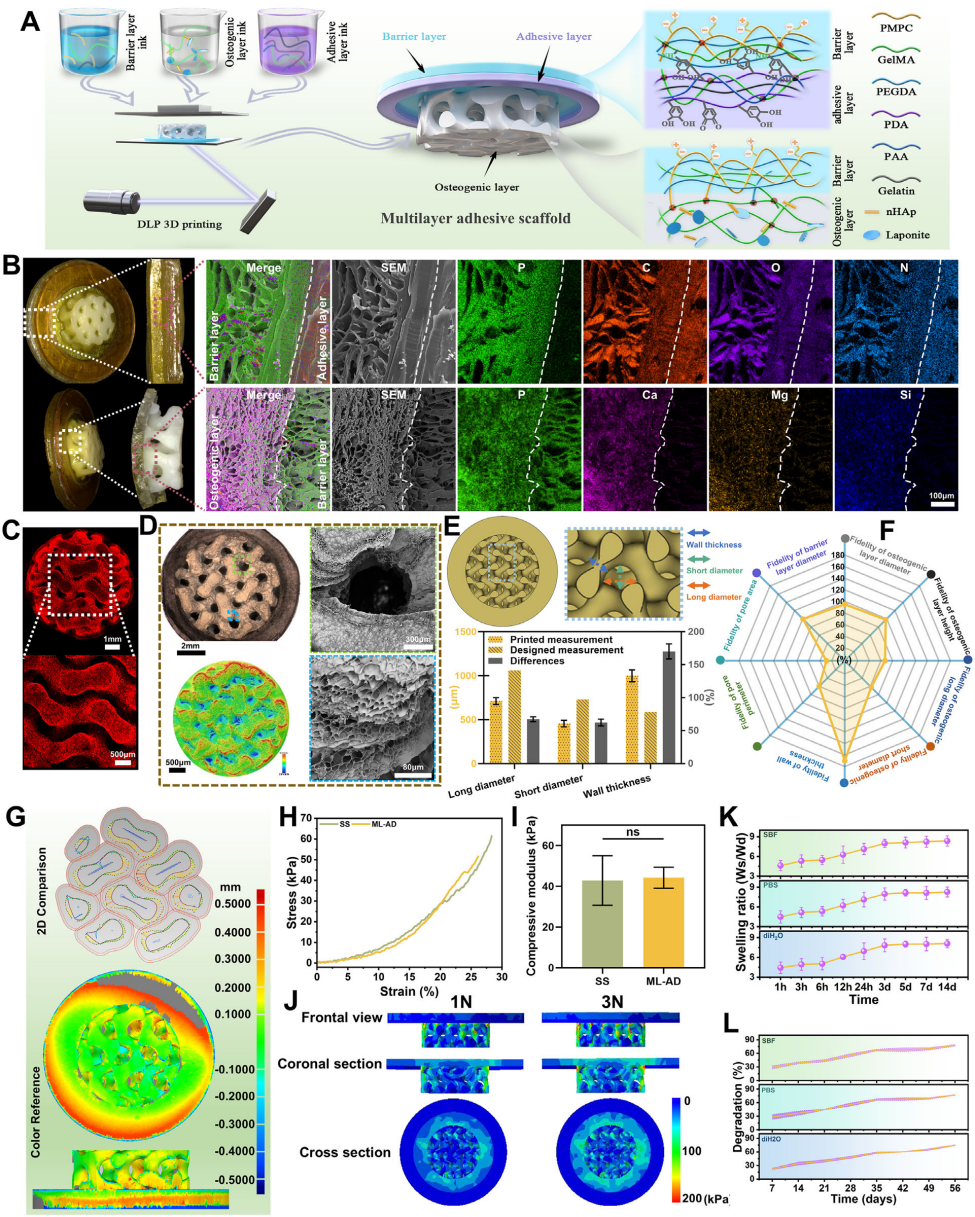
Design, fabrication, and physicochemical characterization of the ML‐AD scaffold. (A) Schematic of the fabrication process and chemical composition of the scaffold. (B) SEM and elemental mapping characterization of the three‐layer internal structure and adjacent interfaces of the scaffold. (C) Rhodamine‐stained image of the scaffold's osteogenic layer. (D) SEM characterization of the osteogenic layer and surface structure contour map. (E) Quantitative analysis of the printing accuracy of the scaffold's osteogenic layer (*N* = 3). (F) Radar chart illustrating the precision of the scaffold (*N* = 3). (G) 3D deviation analysis of the scaffold's osteogenic layer performed using Geomagic Qualify. (H,I) Representative stress–strain curves (H) and compressive moduli (I) from compressive modulus tests of the scaffold and SS (Single scaffold, a single‐layer construct composed solely of the osteogenic layer) (*N* = 3), and unpaired Student's *t*‐test was used for statistical analysis. (J) Stress distribution nephograms of the scaffold under vertical downward forces (1 and 3 N) via finite element analysis (FEA). (K) Swelling kinetics of the scaffold in diH_2_O, PBS, and SBF over time. (L) Degradation profiles of the scaffold in diH_2_O, PBS, and SBF over time (*N* = 3). “ns” indicates no statistical significance (*P *> 0.05).

As shown in Figure 2B, the printed construct accurately reproduced the designed multilayer architecture. SEM and EDS analyses verified the distinct chemical composition of each functional layer and demonstrated seamless interfacial integration—a feature essential for coordinated barrier, adhesive, and osteogenic performance (Figure 2B; Figure S20). The TPMS porous structure of the osteogenic layer was clearly visualized through microscopy and SEM imaging (Figure 2C,D), and elemental mapping analysis from the osteogenic layer (Figure S21) confirms that both nanoparticles were uniformly incorporated into the inner of the osteogenic layer. Scaffold printing fidelity is a pivotal determinant for translating patient‐specific design to successful in‐vivo implantation and intended function, especially for both the membranous and porous layers of this scaffold. A 3D digital microscope was used to measure the printing precision, revealing that the prepared scaffold possesses high precision (Figure 2E,F). Three‐dimensional accuracy, assessed using the Geomagic Qualify software, showed pore deviations within 100 µm, demonstrating the high precision of the DLP‐printed structures (Figure 2G; Figure S22). Furthermore, DLP printing provided excellent design versatility, enabling scaffolds to conform to a variety of defect geometries across tibial, mandibular, and cranial models, underscoring its suitability for personalized bone regeneration (Figure S23).

Mechanical evaluation demonstrated that the ML‐AD scaffold exhibited a compressive modulus of 44.17 ± 5.15 kPa, comparable to that of the single scaffold (SS, only osteogenic layer), indicating that the porous compartment governs the overall mechanical performance (Figure 2H,I). Finite element analysis (FEA) further revealed uniform stress distribution across the scaffold under different loading conditions (vertical, 45°, horizontal) and force magnitudes (1 N, 3 N)— attributable to the TPMS architecture (Figure 2J; Figure S24). These results indicate the scaffold's uniform force‐bearing capacity and structural stability.

As shown in Figure 2K, ML‐AD swelled slowly within 6 h, reached equilibrium on day 3 (in diH_2_O, PBS, SBF), and maintained stability until day 14 with an equilibrium swelling ratio approximately twice the initial value,, indicating the scaffold's dimensional structure stability during 14‐day swelling. Figure 2L shows ∼60% gradual degradation (in solution containing 1 U/mL of type II collagenase) for 35 days followed by slow degradation. Subcutaneous degradation experiments in rabbits were conducted to evaluate the degradation behavior of the ML‐AD scaffold and its individual layers. 3‐month degradation rates were 84.97% ± 6.50% (the osteogenic layer), 79.26% ± 6.40% (the adhesive layer), 43.81% ± 7.54% (the barrier layer), and 62.69% ± 8.59% (ML‐AD) (Figure S25). Encouragingly, the in vivo degradation characteristics of each layer in the designed scaffold roughly meet these requirements: The barrier layer requires slow degradation, while the osteogenic layer requires degradation that matches the rate of bone regeneration (relatively rapid degradation).

### 2.3 Wet‐Adhesive Fixation of the Scaffold Ensures Early Mechanical Stability

Having prepared the multilayer construct, its instant fixation capability was evaluated, a prerequisite for establishing a mechanically stable healing niche. Adhesion performance was first assessed for the optimized dopamine‐containing adhesive layer hydrogel (D0.5), followed by in vitro and in vivo validation of the multilayer adhesive scaffold (adhesion schematic shown in Figure 3A; the test plan displayed in Figure 3B).

**FIGURE 3:**
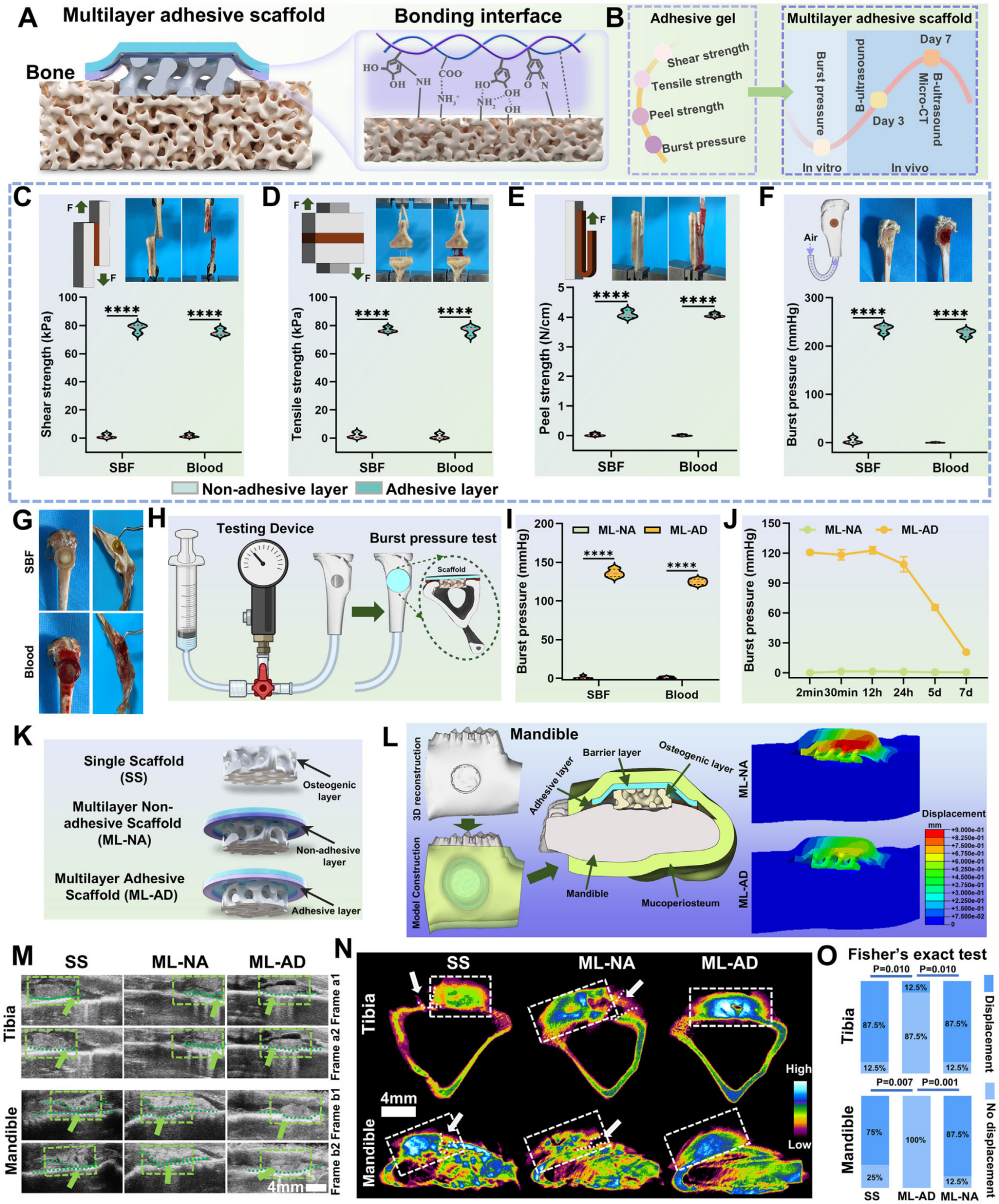
Bone adhesion properties of the non‐adhesive layer and the adhesive layer hydrogels, and the ML‐NA and ML‐AD scaffolds. (A) Schematic illustration of the scaffold adhered to the bone defect site. (B) Schematic of the bone adhesion strength testing plan. (C–F) Bone adhesion strength tests of the adhesive layer and the non‐adhesive layer gels (in the presence of SBF and blood), including shear strength (C), tensile strength (D), peel strength (E), and burst pressure (F) (*N* = 3); two‐way ANOVA was applied to conduct the statistical analysis. (G) Representative images showing the scaffold adhered to the tibial bone defect site in the presence of SBF and blood. (H) Schematic illustration of the burst pressure test apparatus. (I) Burst pressure of ML‐AD and multi‐layer non‐adhesive scaffold (ML‐NA) adhered to tibial bone defect sites in the presence of SBF and blood (*N* = 3); Statistical evaluation was carried out via two‐way ANOVA. (J) Long‐term burst pressure tests (1 week) of ML‐AD and ML‐NA scaffolds adhered to tibial bone defect sites in the presence of SBF (*N* = 3). (K) Schematic illustrations of the ML‐AD scaffold, ML‐NA scaffold, and single scaffold (SS); detailed in Table S4. (L) Displacement contour plots of ML‐AD and ML‐NA scaffolds under simulated mechanical stress post‐implantation in the rabbit mandibular bone augmentation model via FEA. (M) B‐mode ultrasound images (different frames) showing scaffold displacement at 3 days post‐implantation of SS, ML‐NA, and ML‐AD in rabbit mandibular bone augmentation model. (N,O) Micro‐CT images showing the displacement of SS, ML‐NA, and ML‐AD scaffolds at 7 days post‐implantation in the rabbit mandibular bone augmentation model. along with corresponding quantitative analysis (O) (*N *= 4). ****p < 0.0001.

Since the adhesive hydrogel achieves bonding through pressure application, the applied pressure and duration have a significant impact on its adhesive strength. To determine the optimal pressing parameters that maximize the hydrogel's adhesive performance, the optimal conditions (at least 60 s and 8 N) were explored prior to the adhesive strength tests (Figures S26,S27). Dopamine incorporation, which oxidizes to PDA and forms covalent and non‐covalent interactions with bone (coordination, hydrogen bonding, π–π stacking), substantially enhanced the wet‐adhesive performance of the hydrogel [32, 35, 41]. Under SBF and blood, the adhesive layer gel exhibited markedly higher shear, tensile, and peel strengths (in SBF:77.92 ± 3.15 kPa, 76.94 ± 1.88 kPa, and 4.10 ± 0.13 N/cm, respectively; in blood: 75.15 ± 2.43 kPa, 76.32 ± 3.17 kPa, and 4.07 ± 0.06 N/cm, respectively) than the non‐adhesive layer (Figure 3C–E). Tensile testing videos further confirmed tight interfacial bonding (Video S1). Burst‐pressure testing revealed a value of 235.30 ± 8.31 mmHg in SBF or 227.16 ± 7.44 mmHg in blood for the adhesive layer, far exceeding the non‐adhesive layer (Figure 3F), and an in vivo cranial adhesion demonstration in anesthetized rats validated its strong bone‐adhesive capability (Video S2). Further comparison of the bone adhesion performance of the adhesive layer before and after sterilization revealed no significant difference (Figure S28).

The adhesive performance of the multilayer adhesive scaffold was then evaluated. When pressed onto rabbit tibial bone in the presence of SBF or blood, the scaffold remained firmly fixed even when grasped, shaken, or forcibly tugged with forceps (Figure 3G; Video S3). Burst‐pressure testing further quantified its strong adhesion: the multilayer adhesive scaffold (ML‐AD) reached 136.07 ± 5.07 mmHg in SBF and 124.75 ± 3.58 mmHg in blood, whereas the multilayer adhesive scaffold (ML‐NA) showed near‐zero pressure resistance (Figure 3H,I; Video S4). Long‐term wet adhesion testing (simulate humidity only; temperature is 4°C) at rabbit tibial defects (Ex vivo tibia) revealed that although adhesive strength gradually decreased due to water uptake, the scaffold retained measurable adhesion for at least 7 days (65.64 ± 2.89 mmHg at day 5; 20.69 ± 1.24 mmHg at day 7), providing sufficient early mechanical stability (Figure 3J; Video S5).

Because scaffolds are most susceptible to displacement during the early postoperative period—before tissue integration occurs—it was assessed whether adhesive fixation improves positional stability under physiological loading. FEA simulations of a mandibular defect demonstrated markedly reduced displacement for ML‐AD compared with ML‐NA under identical loading directions and magnitudes (the definitions and schematic diagrams of the scaffolds used in this study are shown in Figure 3K,L; Figure S29). Finally, in vivo adhesive fixation was evaluated in rabbit tibial and mandibular superficial defects subjected to daily physiological motion (running and chewing) (Videos S6 and S7). B‐ultrasound monitoring on days 3 and 7 revealed minimal displacement in the ML‐AD group, whereas both the SS and ML‐NA groups exhibited obvious micromotion (Figure 3M; Videos S8,S9; and Figure S30). Micro‐CT at day 7 corroborated these observations: ML‐AD maintained its original position, while SS and ML‐NA showed varying degrees of displacement (Figure 3N,O).

Collectively, these results demonstrate that the ML‐AD scaffold provides robust instant fixation and sustained early mechanical stability at bone defect sites, ensuring a stabilized regenerative niche essential for subsequent healing.

### 2.4 Biocompatibility and Biofunction of the Scaffold

Biocompatibility is a prerequisite for biomaterials to exert their intended functions [42]. Therefore, the multilayer adhesive scaffold was comprehensively assessed in vitro and in vivo in terms of biocompatibility. BMSC proliferation over 7 days showed normal growth across all groups, and RAW264.7 macrophages and HUVECs cultured with the scaffold exhibited favorable cell viability, confirming excellent cytocompatibility (Figures S31,S32). Live/Dead and cytoskeletal staining further demonstrated that BMSCs readily adhered, spread, and proliferated within the porous architecture of the scaffold (Figure S33). Further cell viability assays using the degradation media showed that BMSCs cultured with the scaffold maintained high viability at both 5 days and 4 weeks, demonstrating the excellent cytocompatibility of the long‐term degradation products (Figure S34). In vivo histocompatibility is equally important. Hemolysis testing verified good hemocompatibility (Figure S35), and H&E staining during degradation studies revealed no significant inflammatory response at the implantation site (Figure S25A). Moreover, histological evaluation of major organs (heart, liver, spleen, lungs, kidneys) at 1 month post‐implantation showed normal morphology comparable to the blank control, indicating an absence of systemic or organ toxicity (Figure S36). Collectively, these results demonstrate excellent biocompatibility of the multilayer adhesive scaffold.

While the adhesive layer provides instant fixation to maintain a mechanically stable healing niche, the bioactivity of the osteogenic layer is essential for promoting BMSC infiltration and osteogenic differentiation (schematic illustration of experimental plan displayed in Figure 4A). Scratch and transwell assays showed that BMSC migration in the ML‐AD and osteogenic layer groups was significantly higher than in other groups (*p* < 0.05) (Figure 4B,F,G; Figure S37). ALP staining and ALP activity assays further revealed markedly higher osteogenic activity in the scaffold and osteogenic layer groups compared with other groups (*p* < 0.05), with no significant difference between ML‐AD and osteogenic layer groups—consistent with the staining results (Figure 4C,H). Mineralization assays, including ARS staining at day 14 and 21 of culture (Figure 4D,I) and Von Kossa staining at day 14 of culture (Figure 4E), followed the same trend. Expression of osteogenesis‐related genes (RUNX2, OSX, ALP, Col‐1, OCN, and OPN) at day 7 and 14 of culture and proteins (OCN and OPN) at day 7 of culture was also significantly upregulated in the scaffold and osteogenic layer groups (Figure 4J–O). Furthermore, BMSCs cultured on the scaffolds also exhibited a pronounced osteogenic differentiation effect (Figure S38). These findings indicate that the scaffold effectively promotes osteogenic differentiation—an effect attributable primarily to the osteogenic porous layer. Importantly, the adhesive layer alone showed negligible osteogenic activity, which is advantageous as it allows the adhesive performance to be evaluated independently from osteogenic effects when assessing in vivo bone regeneration.

**FIGURE 4:**
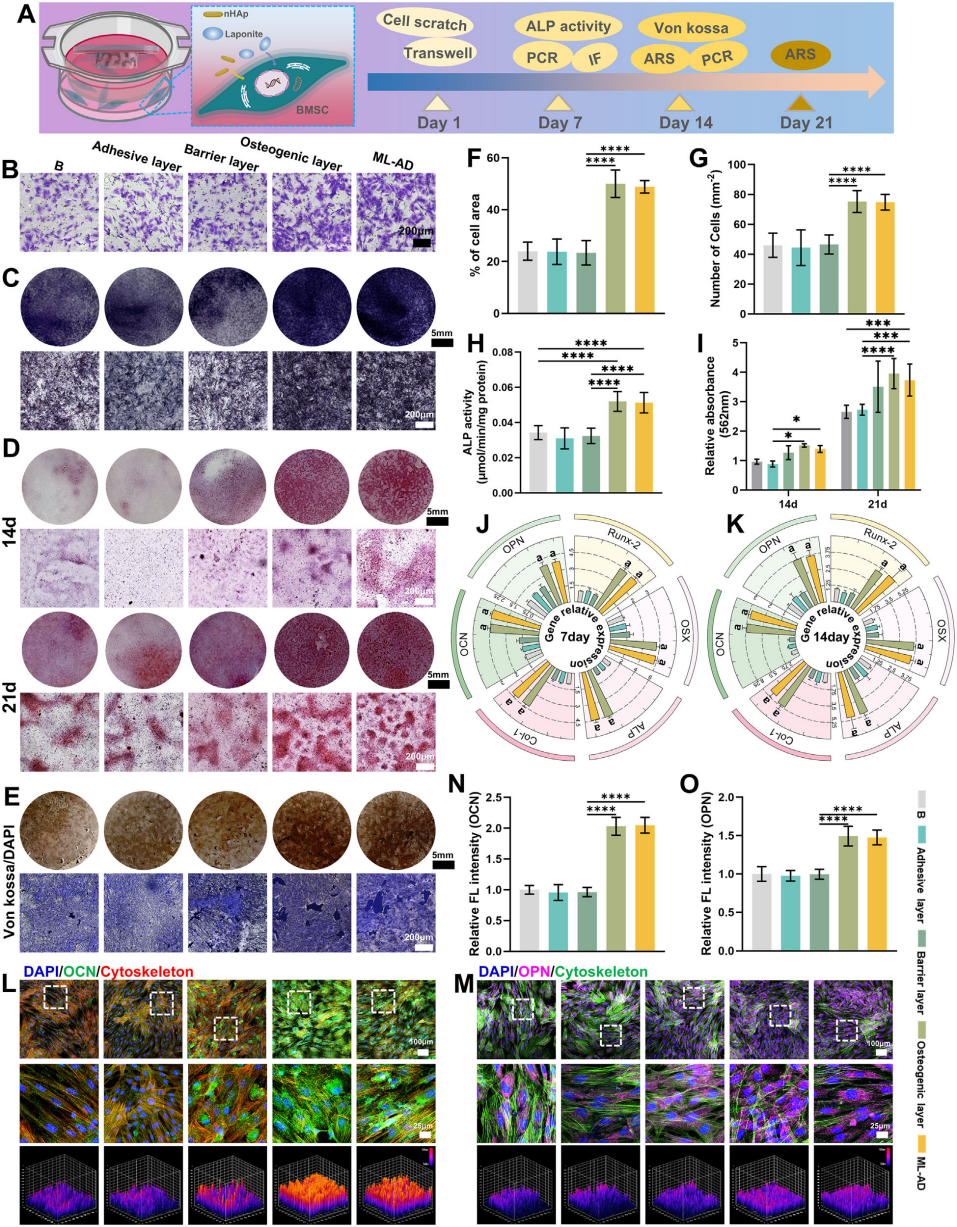
Bioactivity of the ML‐AD scaffold and its individual layers: barrier layer, adhesive layer and osteogenic layer. (A) Schematic diagram of the overall in vitro experimental plan for evaluating the scaffold's bioactivity. (B) Representative images of BMSC migration capacity tested via Transwell assay under the scaffold and its individual layer interventions. (C) Representative ALP staining images of BMSCs after 7 days of co‐culture with the scaffold and its individual layers. (D) Representative alizarin red S (ARS) staining images of BMSCs cultured with the scaffold and its individual layers for 14 and 21 days. (E) Representative Von Kossa staining images of BMSCs after 14 days of co‐culture with the scaffold and its individual layers. (F,G) Quantitative analyses of the Transwell assay, including the area ratio (F) and number ratio (G) of migrated BMSCs (*N* = 3). (H) Quantitative analysis of ALP activity in BMSCs following 7 days of co‐culture with the scaffold and its individual layers (*N* = 3). (I) Quantitative analysis of ARS staining in BMSCs cultured with the scaffold and its individual layers for 14 and 21 days (*N* = 3). (J,K) Quantitative analysis of osteogenesis‐related gene expression in BMSCs determined by RT‐qPCR after 7 days (J) and 14 days (K) of co‐culture with the scaffold and its individual layers (*N* = 3); “a” denotes that *p* < 0.05 compared with blank control (B), adhesive layer and barrier layer. (L–O) Representative immunofluorescence staining images and corresponding quantitative analyses of the expression of osteogenesis‐related proteins osteocalcin (OCN) (L,N) and osteopontin (OPN) (M,O) in BMSCs after 7 days of co‐culture with the scaffold and its individual layers (*N* = 3). B: blank control group. Statistical significance in this figure was assessed by one‐way ANOVA. *p < 0.05; ***p < 0.001; ****p < 0.0001.

In vitro validation for barrier function of the barrier layer of the scaffold demonstrated that the barrier layer can effectively prevent cell (rat gingival fibroblasts and skin fibroblasts) penetration, potentially isolating soft tissues (which impede osteogenesis) from the bone regeneration area (Figure S39).

### 2.5 In Vivo Bone Augmentation Study of the Scaffold in Rabbits

After establishing the in vitro performance of the multilayer adhesive scaffold, we next examined whether its instant‐fixation capability could enhance bone regeneration under physiologically dynamic conditions. Mandibular and tibial bone augmentation models were established in rabbits, representing two anatomical sites subjected to continuous micromotion from mastication and ambulation. In these regions, implant fixation is challenging, and implant displacement frequently compromises clinical outcomes. A shallow, mild 0.5‐mm deep defect model was adopted to mimic the clinically common condition of insufficient maxillary bone volume caused by bone resorption or congenital defects.

Six experimental groups were included: B (no implant materials used), CM‐BP (collagen membrane+bone powder, conventional clinical treatment (GBR)), CM‐BP‐P (collagen membrane+ bone powder+ pins, conventional clinical treatment (GBR) with fixation), SS (single scaffold), ML‐NA (multilayer non‐adhesive scaffold), and ML‐AD (multilayer adhesive scaffold) (detailed in Table S4). After the surgery (Figure S40), the rabbits were allowed normal feeding and subjected to 30‐min daily running exercises for 7 days onward to simulate physiological loading, ensuring that the mandible and tibia maintained physiological movement patterns (Videos S6,S7). Bone regeneration was assessed by micro‐CT, histological staining, and immunostaining at 1 and 2 months postoperatively.

Interestingly, the ML‐AD scaffold substantially simplified the surgical operation. In both mandibular and tibial models, implantation of the ML‐AD scaffold required only ∼65 s—representing a 60%–70% time reduction relative to CM‐BP and an 85%–90% reduction relative to CM‐BP‐P (Figure S41; Videos S10,S11). The scaffold avoided bone powder shaping, membrane trimming, and pin fixation, demonstrating excellent practicality for clinical translation.

At 1 and 2 months postoperatively, the rabbits were euthanized, dissected, and fixed in 4% paraformaldehyde. Micro‐CT scanning and quantitative analysis were performed to radiographically evaluate the osteogenic efficacy of the scaffolds (Figure 5A). Micro‐CT analysis at 1 and 2 months revealed clear differences in bone augmentation across groups. In both the mandibular and tibial models, all groups (including the B group) showed substantial bone filling, which can be attributed to the relatively mild initial defect (Figure 5B,C). However, it is worth noting that the model used in this study was designed as a bone augmentation model rather than a critical‐sized bone defect regeneration model. To interrogate the central design principles of our scaffold system, we focused on four comparisons: (i) SS versus ML‑NA, to isolate the effect of the multilayer barrier architecture; (ii) ML‑NA versus ML‑AD, to assess the contribution of adhesive fixation with matched chemistry; and (iii) ML‑AD versus CM‑BP‑P or CM‐BP, to benchmark against a clinical‑grade comparator.

**FIGURE 5:**
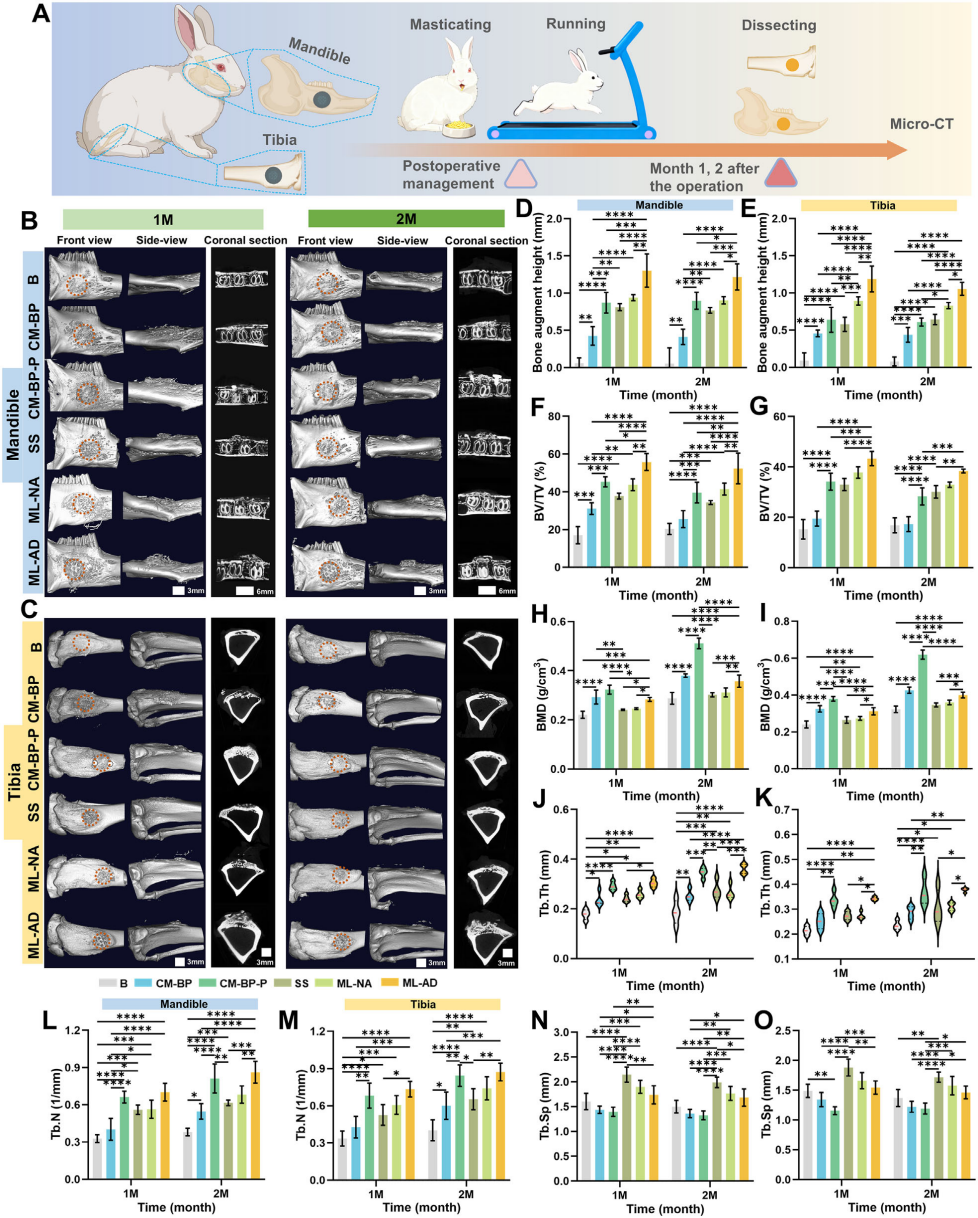
Micro‐CT analysis of bone augmentation effects of CM‐BP, CM‐BP‐P, and SS, ML‐NA, and ML‐AD scaffolds in rabbit mandibular and tibial sites. (A) Schematic of bone augmentation model construction, postoperative management, and planning for rabbit mandibles and tibiae. (B,C) Representative frontal, lateral, and coronal‐sectional views of Micro‐CT 3D reconstructions of bone augmentation regions in the mandible (B) and tibia (C) at 1 and 2 months post‐implantation of clinically commonly used treatments (CM‐BP and CM‐BP‐P), SS, ML‐NA, and ML‐AD scaffolds; detailed experimental grouping is presented in Table S4. (D–O) Quantitative analysis of Micro‐CT data including bone augmentation height (mandible (D), tibia (E)), BV/TV (mandible (F), tibia (G)), bone mineral density (BMD) (mandible (H), tibia (I)), trabecular thickness (Tb.Th) (mandible (J), tibia (K)), trabecular number (Tb.N) (mandible (L), tibia (M)), and trabecular separation (Tb.Sp) (mandible (N), tibia (O)) (*N* = 4). All statistical analyses in this figure were implemented using two‐way ANOVA. *p < 0.05; **p < 0.01; ***p < 0.001; ****p < 0.0001.

In both mandibular and tibial defects, the ML‑AD group consistently produced the highest bone augmentation among all groups, with augmentation heights exceeding 1 mm at both time points (Figure 5B–E). Compared with ML‑NA, the ML‑AD group showed significantly greater augmentation height and BV/TV (Figure 5D–G), demonstrating that adhesive fixation substantially enhances osteogenic outcomes beyond the benefit of the multilayer architecture alone. Notably, this improvement is attributed to mechanical stabilization rather than compositional differences, as ML‑AD and ML‑NA share identical chemical formulations. The effect of the barrier layer was evident when comparing SS and ML‑NA: ML‑NA yielded significantly higher augmentation heights and BV/TV than SS, particularly in the tibial model (Figure 5D–G), implying that the barrier layer effectively contributed to bone augmentation. Furthermore, comparison between ML‑AD and the clinical‑grade comparator groups (CM‑BP and CM‑BP‑P) based on Micro‑CT data revealed that ML‑AD achieved significantly greater bone augmentation than both CM‑BP and CM‑BP‑P (Figure 5B–G). These results demonstrate that the ML‑AD scaffold outperforms clinical GBR‐like approaches in promoting bone augmentation.

While CM‑BP‑P exhibited the highest BMD among all groups‐likely due to residual dense hydroxyapatite‐the ML‑AD group demonstrated superior mineralization among the engineered scaffolds (Figure 5H,I). Furthermore, trabecular microarchitecture parameters (Tb.Th, Tb.N, and Tb.Sp) consistently indicated denser and more mature bone structure in the ML‑AD group (Figure 5J–O). Collectively, these data reveal that ML‑AD outperforms both conventional scaffolds and clinical GBR‐like procedures in rabbits, with adhesive fixation serving as the key driver for enhanced bone augmentation.

The decalcified samples were embedded, sectioned, and subjected to histological staining (Figure 6A). HE and Goldner staining analysis corroborated Micro‐CT findings (Figure 6B,C). There is no significant bone augmentation in the defect area of Group B, which was covered by soft tissue, whereas CM‐BP and CM‐BP‐P showed residual bone powder encapsulated by new bone. In the SS, ML‐NA, and ML‐AD groups, new bone formed throughout the scaffold interior, with ML‐AD exhibiting the greatest augmentation height on histological sections (Figure 6D,E). Quantitative analysis based on Goldner staining further revealed results consistent with the Micro‑CT findings (Figure S42). Moreover, histological evaluation (HE) confirmed robust osseointegration between the newly formed bone and the native host bone (Figure S43). Notably, histological augmentation height comparison between the SS and ML‐NA groups revealed that the ML‐NA group achieved significantly higher augmentation heights than the SS group at both 1 and 2 months (Figure 6D,E), indicating that the addition of the barrier layer of the scaffold improves bone augmentation height. Complementing these findings, Goldner staining provided direct visual evidence that the barrier layer effectively inhibited the ingrowth of surrounding soft tissue into the scaffold, thereby maintaining a protected osteogenic compartment (Figure S44). For TRAP staining, ML‐NA and SS showed higher osteoclast activity (TRAP^+^) than ML‐AD, explaining their greater scaffold degradation and reduced regenerative volume (Figure S45). Enhanced angiogenesis (CD31^+^) and early osteogenic activity (OCN^+^) were evident in ML‐AD at 1 month post‐implantation (Figure 6F, G, I and J) by immunofluorescence staining, while IHC staining for OCN and BMP‐2 demonstrated sustained osteogenesis in the ML‐AD group (Figure 6H,K; Figure S46).

**FIGURE 6:**
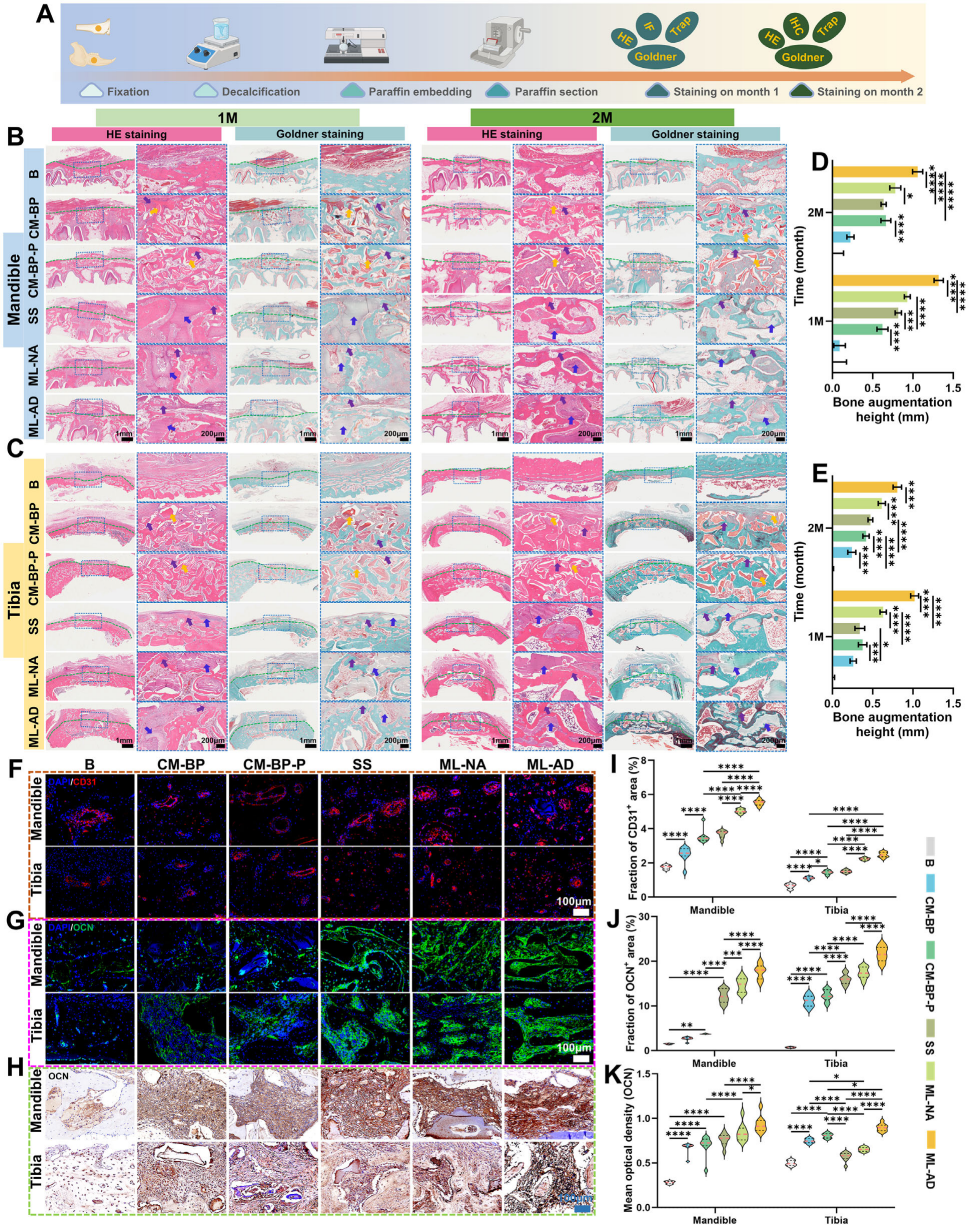
Histological analysis of bone augmentation regions at 1 and 2 months post‐implantation in rabbits. (A) Schematic illustration of the histological analysis procedure. (B,C) Representative HE staining and Goldner staining images of bone augmentation regions in the mandible (B) and tibia (C) at 1 and 2 months postoperatively; blue arrows indicate the scaffold material; yellow arrows indicate the bone powder; purple arrows indicate the newly formed bone. (D,E) Quantitative measurement of bone augmentation height in the mandible (D) and tibia (E) based on Goldner staining (*N* = 4). (F,G) Representative immunofluorescence staining images of CD31 (F) and OCN (G) in bone augmentation sites of the mandible and tibia at 1 month postoperatively, and representative immunohistochemical (IHC) staining images of OCN (H) at 2 months postoperatively; corresponding quantitative analyses include immunofluorescence staining of CD31 (I) and OCN (J), and IHC staining of OCN (K) (*N* = 4). Two‐way ANOVA was used for all statistical analyses in this figure. *p < 0.05; **p < 0.01; ***p < 0.001; ****p < 0.0001.

Taken together, across anatomical sites, imaging modalities, and histological assessments, the multilayer adhesive scaffold (ML‐AD) consistently achieved superior bone augmentation. Given the identical chemical composition of ML‐AD and ML‐NA, these results suggest that adhesive‐driven mechanical stability may serve as a key contributing factor associated with the enhanced bone regeneration observed in vivo.

### 2.6 Exploration of Potential Mechanisms of Instant‐Fixation on Promoting Bone Augmentation in Rats

To investigate how adhesive‐driven mechanical stability enhances bone regeneration, a rat tibial augmentation model—parallel to the rabbit model—was established (Figure 7A; Figure S47). The ML‐NA served as the critical control, sharing identical chemistry and architecture with ML‐AD except for adhesive fixation. At 3 and 7 days post‑surgery, B‑mode ultrasound analysis revealed that scaffold micromotion in the ML‑NA group was significantly greater than that in the ML‑AD group (Figure 7B; Video S12), indicating that adhesive fixation effectively reduces scaffold micromotion and preserves mechanical stability of the osteogenic region. Furthermore, Micro‑CT quantitative analysis at 1 month post‑surgery demonstrated that the ML‑AD group exhibited significantly greater bone augmentation height and volume compared with the ML‑NA group (Figure 7C). Collectively, these findings reveal that adhesive fixation‑mediated mechanical stability promotes bone augmentation in rats, consistent with observations in rabbits, thereby supporting the rationale for using rats as a substitute for rabbits in mechanistic studies.

**FIGURE 7:**
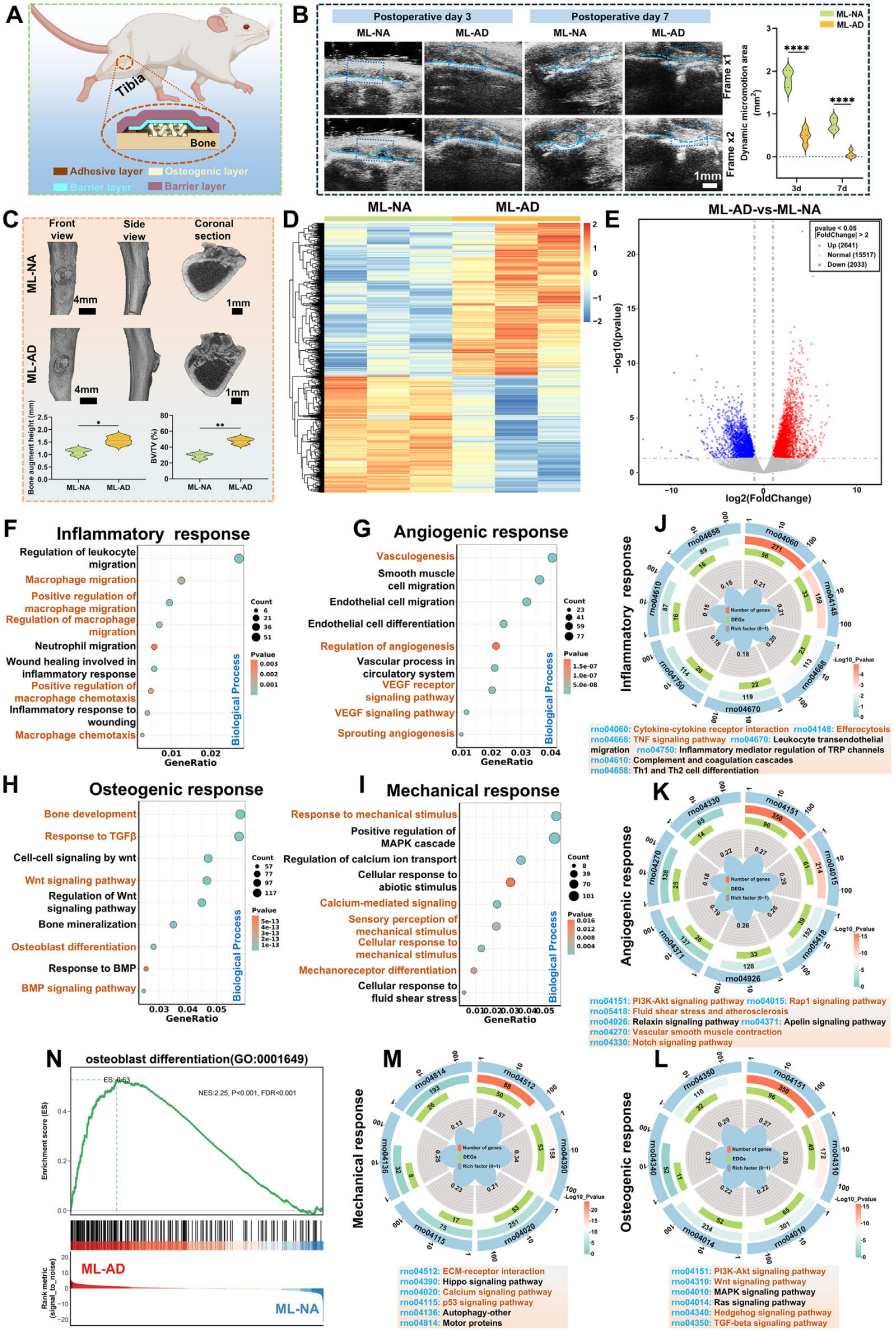
Bone augmentation effects and RNA‐seq analysis of ML‐NA and ML‐AD scaffolds in a rat tibial bone augmentation model. (A) Schematic illustration of scaffold implantation into the tibia in the rat tibial bone augmentation model. (B) Ultrasound‐based assessment of scaffold micromotion at 3 and 7 days following implantation of ML‐NA and ML‐AD scaffolds in rat tibia (*N* = 3); unpaired Student's *t*‐test was used for statistical analysis. (C) Micro‐CT assessment of bone augmentation induced by ML‐NA and ML‐AD scaffolds in rat tibiae at 1 month post‐implantation, including representative images and quantitative data of BV/TV and bone augmentation height (*N* = 3); statistical analyses were performed via one‐way ANOVA. (D) Clustering heatmap of significantly differentially expressed genes (DEGs) between ML‐AD and ML‐NA (*N* = 3). (E) Volcano plot of significantly DEGs between ML‐AD and ML‐NA (*N* = 3). (F–I) Bubble chart of GO‐BP enrichment analysis for DEGs between ML‐AD and ML‐NA, including inflammatory response (F), angiogenic response (G), osteogenic response (H), and mechanical response (I) (*N* = 3). (J–M) Circos plots of KEGG pathway enrichment analysis of DEGs between ML‐AD and ML‐NA, encompassing inflammatory response (J), angiogenic response (K), osteogenic response (L), and mechanical response (M) (*N* = 3). (N) GSEA analysis for osteoblast differentiation (*N* = 3, *FDR* < 0.05).

Transcriptomic sequencing (RNA‐seq) was performed at day 7 post‐surgery, followed by targeted experimental validation. RNA‐seq showed satisfactory intra‐group consistency (Figure S48). Compared with ML‐NA, the ML‐AD group displayed extensive transcriptional reprogramming, with 2641 upregulated and 2033 downregulated DEGs (Figure 7D,E). GO enrichment revealed significant activation of biological processes central to bone regeneration, including inflammatory response, angiogenic response, osteogenic response, and cell migration (Figure 7F–H; Figure S49). Immune‐related GO terms indicated altered macrophage recruitment and differentiation, while angiogenic (e.g., VEGF signaling pathway, etc.) and osteogenic pathways (e.g., Wnt signaling pathway, BMP signaling pathway, etc.) were likewise enhanced in ML‐AD. Chemotaxis response was also upregulated, suggesting significantly enhanced cell chemotaxis to the bone regeneration site, a critical prerequisite for osteogenesis. KEGG and GSEA enrichment analyses further validated these findings (Figure 7J–L,N; FiguresS50–S52).

Notably, ML‐NA and ML‐AD were identical in all aspects (chemical composition and structural configuration) except for the presence of adhesive fixation in the ML‐AD group. This experimental design ensures that the observed differential enrichment of bone‐forming‐related genes and pathways can be attributed to the mechanical response triggered by adhesive fixation. Strikingly, GO enrichment analysis (Figure 7I) and KEGG pathway analysis (Figure 7M) consistently identified significant enrichment of differential genes associated with mechanical response (e.g., response to mechanical stimulus, mechanoreceptor differentiation, and cellular response to mechanical stimulus). These findings collectively suggest that the adhesive fixation of the ML‐AD scaffold mediates cellular behaviors and functions in the osteogenic microenvironment through affecting mechanical responses, thereby regulating bone regeneration. Together, these results indicate that adhesive fixation stabilizes the niche and affects cellular responses to mechanical stimuli, which in turn may modulate inflammatory, angiogenic, and osteogenic programs.

Integrating the micromotion data, bone augmentation performance, sequencing findings, and published evidence, we hypothesize that the marked micromotion of ML‑NA induces detrimental mechanical cues, promoting a pro‑inflammatory macrophage shift that impairs angiogenesis–osteogenesis coupling and hinders bone augmentation, whereas ML‑AD achieves the opposite via adhesive stabilization. To test this hypothesis, a rat tibial bone augmentation model was established, and the samples were harvested at 7 and 14 days postoperatively for follow‐up experiments. Flow cytometry testing demonstrated greater macrophage recruitment (CD68^+^) into ML‐AD scaffolds. Subsequent profiling revealed substantially higher M2‐like polarization (CD206^+^) and reduced M1‐like polarization (CD86^+^) in ML‐AD at 7 and 14 days postoperatively (Figure 8A,B; Figures S53,S54), with multiplex immunofluorescence staining (CD68/iNOS/CD163) demonstrating this trend (Figure S55). Functionally, ML‐AD scaffolds exhibited significantly higher numbers of CD68^+^BMP‐2^+^ and CD68^+^VEGF^+^ macrophages (Figure 8C–E), which in turn enhanced angiogenesis and osteogenic differentiation, including abundant H‐type vessels (Figure 8C,F; FigureS56) and upregulated osteogenic differentiation markers (OSX, OPN) in the ML‐AD group (Figure 8C,G; Figure S57).

**FIGURE 8:**
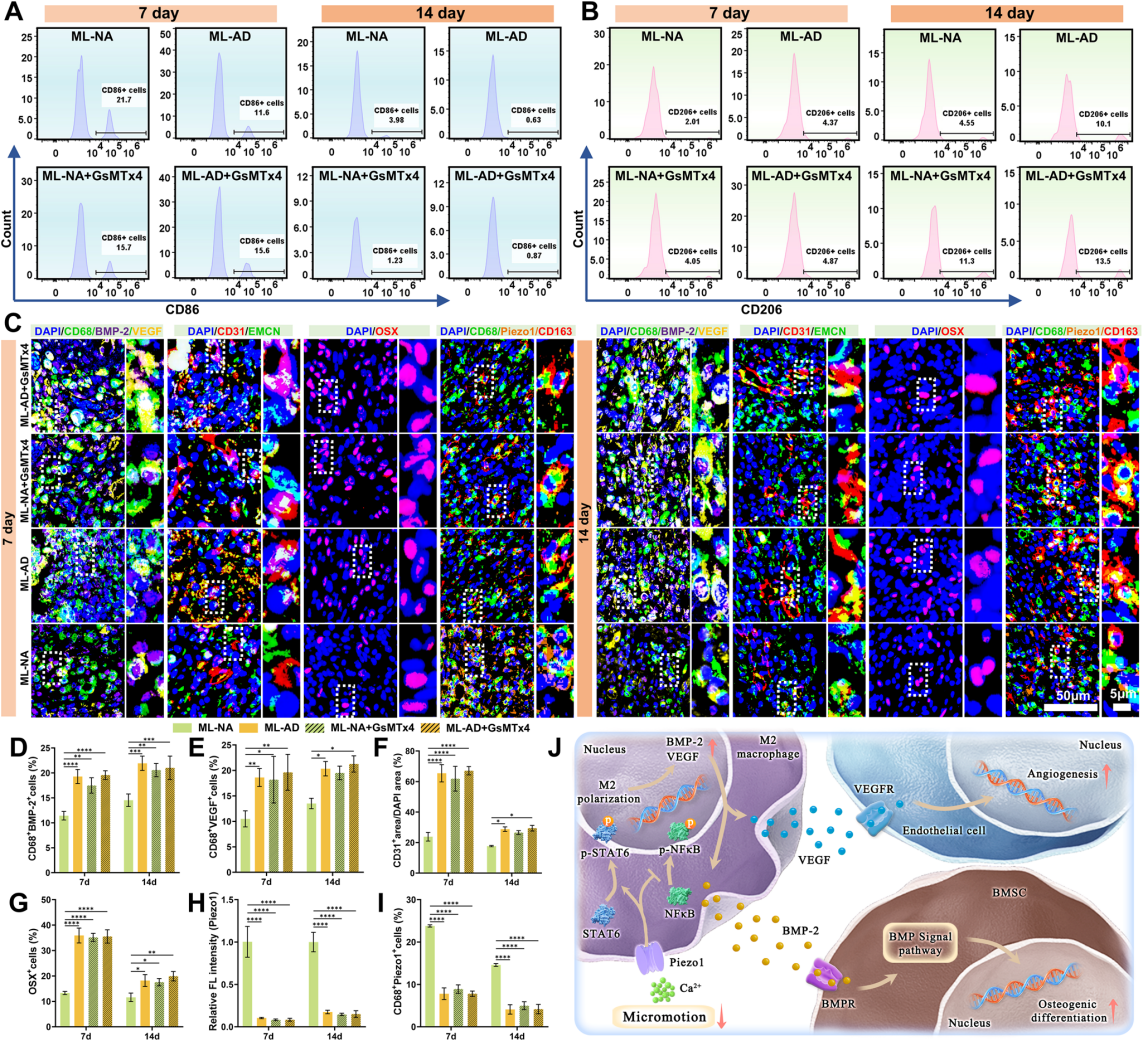
Exploration of the bone augmentation‐promoting potential mechanism of the ML‐AD scaffold using a rat tibial bone augmentation model, with or without pharmacological inhibition of Piezo1 using GsMTx4. (A,B) Flow cytometry analysis of macrophage profiles within the scaffold at 7 and 14 days postoperatively (*N* = 3). (C) Representative immunofluorescence staining images (CD68/BMP‐2/VEGF, CD31/EMCN, OSX, and CD68/Piezo1/CD163) of the bone augmentation region at 7 and 14 days postoperatively. (D–I) Semi‑quantitative immunofluorescence analysis of the local biological responses, including (D) CD68^+^BMP‑2^+^ cells, (E) CD68^+^VEGF^+^ cells, (F) CD31^+^ area, (G) OSX^+^ cells, (H) Piezo1 relative fluorescence intensity, and (I) CD68^+^Piezo1^+^ cells (*N* = 3). (J) Schematic illustration of the regulatory effect of scaffold adhesive fixation on osteogenic cascade events. Statistical analyses in this figure were performed via two‐way ANOVA. *p < 0.05; **p < 0.01; ***p < 0.001; ****p < 0.0001.

In the early stages of bone regeneration, macrophage recruitment and polarization play a pivotal role [43]. Given that macrophage polarization is highly sensitive to mechanical cues, we hypothesized that the differential macrophage polarization observed between the ML‑NA and ML‑AD groups may be attributed to distinct mechanical stimuli arising from scaffold micromotion, potentially mediated by Piezo1. To test this hypothesis, additional groups were included with Piezo1 inhibitor (GsMTx4) treatment, namely ML‑NA+GsMTx4 and ML‑AD+GsMTx4. Immunofluorescence co‐staining for CD68/Piezo1/CD163 revealed that Piezo1 expression and the number of CD68^+^Piezo1^+^ macrophages were significantly lower in the ML‑AD, ML‑NA+GsMTx4, and ML‑AD+GsMTx4 groups compared with the ML‑NA group (Figure 8C,H,I; Figure S58), demonstrating successful Piezo1 inhibition in the ML‑NA+GsMTx4 group. Furthermore, combined flow cytometric and immunofluorescence analyses demonstrated that Piezo1 inhibition reversed the macrophage phenotype shift in the ML‑NA group, promoting an M2‑like polarization (Figure 8A,B; Figure S53–S55). Notably, the ML‑AD, ML‑NA+GsMTx4, and ML‑AD+GsMTx4 groups showed comparable M2‑like polarization levels, all significantly superior to that of the ML‑NA group (Figure 8A,B; Figures S53–S55). These trends were consistently observed across multiple immunostaining panels, including CD68/Piezo1/iNOS, CD68/p‑STAT6, and CD68/Piezo1/p‑NFκB p65 (Figures S59–S61). In line with the macrophage polarization findings, the subsequent immunostaining for angiogenic and osteogenic markers revealed analogous trends (Figure 8C–G; Figures S56,S57). Together, these findings may support our hypothesis that the unfavorable micromotion of ML‑NA generates detrimental mechanical cues, which drive Piezo1‑mediated pro‑inflammatory macrophage polarization through mechanotransduction, impair angiogenesis–osteogenesis coupling, and ultimately lead to poor bone augmentation—whereas ML‑AD achieves the opposite through adhesive fixation.

Integrated RNA‐seq analysis together with subsequent experimental validation suggests that the ML‐AD scaffold may enhance bone regeneration by creating a mechanically stabilized healing niche through its adhesive fixation. This early stability favors a pro‐regenerative mechano‐immune environment within the osteogenic region of the scaffold, which facilitates macrophage recruitment and M2‐like polarization, as shown by increased macrophage migration into the scaffold, elevated phosphorylation and nuclear translocation of STAT6, downregulation phosphorylation of the NFκB, and ultimately enhanced M2‐associated macrophage polarization. These mechanoimmunity‐related signals are associated with a bias of macrophages toward an M2‐like phenotype, along with elevated secretion of VEGF and BMP‐2—factors that are collectively linked to improved angiogenesis and osteogenic differentiation. Through this cascade of cellular events potentially orchestrated by mechanical stabilization conferred by adhesive fixation, the ML‐AD scaffold may contribute to the development of a mechanoimmune—vascular–osteogenic microenvironment that is correlated with promoting bone formation (Figure 8J).

## 3 Discussion

Inspired by the applications of diverse biomimetic scaffolds and multi‐layered functional scaffolds in tissue regeneration [22, 44, 45], as well as the emerging paradigm that mechanical stability of the osteogenic niche is an important factor in scaffold‐mediated bone regeneration [1, 2, 3, 4, 5], we designed and fabricated an ML‐AD scaffold integrating barrier, adhesive, and osteogenic layers as a unified system (Scheme 1). Upon implantation, the scaffold provides instant adhesive bonding to the defect site, secures early‑stage mechanical stability for the osteogenic niche, and thereby facilitates bone augmentation. Through layer‐specific formulation optimization and multimaterial DLP 3D printing, the scaffold achieves robust wet adhesion to bone defects via a simple finger‐press application, analogous to bandage‐like fixation. To test the efficiency of the scaffolds, we selected two representative bone augmentation models, including rabbit mandibular and tibial augmentation models widely used in therapeutic approach in oral clinic, but remain a major clinical challenge due to their high motion, making it difficulty in securing the implant material—two anatomically and biomechanically demanding sites characterized by continuous micro‐movements from mastication and ambulation. In these challenging defects of rabbits, the ML‐AD scaffold markedly enhanced bone augmentation compared with non‐adhesive scaffolds (SS and ML‐NA, >30% in augmentation height), and even outperformed the specific GBR‐like comparator used in this study (CM‐BP and CM‐BP‐P, >35% in augmentation height). It is worth noting that excessive bone formation outside the intended defect region could potentially lead to abnormal protrusions or undesirable interactions with surrounding tissues [46, 47, 48]. To explore this, we further examined the interface between the newly formed bone and surrounding tissues using high‐magnification H&E histological images of the defect‐edge region (Figure S43). The images revealed direct bony bridging between the newly formed bone and the host bone. The newly regenerated bone extended inward from the host bone margin toward the scaffold interior and the defect center, with minimal outgrowth beyond the scaffold and no obvious fibrous tissue intervention. These findings demonstrate favorable integration between regenerated and host bone, indicating that the observed bone formation is well‐regulated and structurally organized, rather than ectopic or excessive.

DLP‐based 3D printing enables fabrication of multilayer scaffolds with a porous osteogenic niche and patient‐specific conformability, allowing precise adaptation to diverse defect geometries and broad clinical applicability [49]. Incorporation of a top barrier layer that recapitulates the function of GBR membranes effectively excludes soft‐tissue infiltration while mechanically coupling the adhesive and osteogenic layers. Consistent with prior studies highlighting the importance of space maintenance in bone regeneration [22], in vivo analyses demonstrated that the barrier‐containing ML‐NA scaffold promoted superior bone formation compared with the single scaffold (SS). In addition, the slow degradation of the barrier layer (about 44% at 3 months) is consistent with its functional requirements, and its good biocompatibility suggests a low risk of adverse effects in vivo. Nevertheless, further optimization of its degradation behavior and long‑term biocompatibility evaluation are required. Critically, the integrated adhesive layer in ML‐AD provides instantaneous and robust anchorage (burst pressure > 120 mmHg), which is particularly important during the early postoperative period when implants are most susceptible to displacement. Sustained positional stability of ML‐AD under physiological motion was further tested in vivo by B‐mode ultrasound. In contrast to conventional fixation strategies such as screws or titanium meshes, embedding a degradable bioadhesive directly within the scaffold simplifies fixation, eliminates the need for secondary removal procedures, and reduces both surgical trauma and economic burden. While multilayer BTE scaffolds have been explored to integrate multiple functions, adhesive fixation has rarely been treated as a core design principle—particularly for defects with poor intrinsic retention [22, 50]. By addressing this unmet clinical need, ML‐AD streamlines surgical workflows while achieving superior regenerative outcomes, underscoring its strong translational potential. Notably, despite its excellent bone augmentation in the dynamic defect model, the scaffold's elastic modulus (∼44 kPa) may preclude its use in high‑load repair of large structural bone defects, and further improvement may be required.

Beyond structural performance, this study provides mechanistic insight into how fixation‐mediated mechanical stability regulates bone augmentation. Mechanical stabilization has long been clinically recognized as essential for fracture healing and GBR success [10, 51, 52, 53], yet the potential underlying biological mechanisms remain incompletely understood. Transcriptomic analysis comparing ML‐AD and ML‐NA—two scaffolds sharing identical chemistry and architecture but differing in adhesive fixation—revealed that adhesive fixation significantly upregulated gene programs associated with immune regulation, angiogenesis, osteogenesis, and cell migration, all of which are central to successful bone regeneration. Consistently, flow cytometry and immunofluorescence analyses suggest that ML‐AD promoted macrophage recruitment and favored M2‐like polarization, accompanied by elevated VEGF and BMP‐2 secretion, enhanced angiogenesis, and increased osteogenic differentiation. The early inflammatory phase is critical for establishing an osteogenesis‐conducive microenvironment [10]. Notably, macrophages play a central role during the early inflammatory state [54]. and exhibit mechanosensitivity with a nonlinear response to local mechanical cue: moderate stimulation favors M2 polarization, while excessive stimulation suppresses M2 polarization and biases the M1 phenotype [13, 55, 56]. Thus, combining further RNA‐sequencing analysis with differential enrichment in mechanical response, we propose that adhesive fixation in ML‐AD suppresses scaffold micromotion, thereby regulating an immune microenvironment that favors M2‐like macrophage polarization. Supporting this hypothesis, Piezo1—a mechanosensitive ion channel whose expression scales with mechanical load [57, 58]—was markedly reduced in macrophages within ML‐AD scaffolds. Mechanistically, Piezo1 activation in macrophages induces calcium influx, which promotes NFκB phosphorylation and nuclear translocation to drive M1 polarization, while inhibiting STAT6 phosphorylation and nuclear translocation to suppress M2 polarization [55, 59]. In agreement, ML‐AD exhibited reduced Piezo1 and nuclear localization of p‐NFκB, and enhanced STAT6 activation in macrophages, suggesting a mechanotransductive shift toward a pro‐regenerative immune phenotype. Importantly, the administration of the Piezo1 inhibitor (GsMTx4) in the ML‑NA+GsMTx4 group effectively reversed the adverse immune phenotype and enhanced the angiogenic and osteogenic activities observed in the ML‑NA group, bringing them to levels comparable to those in the ML‑AD and ML‑AD+GsMTx4 groups. Notably, all three GsMTx4‑treated or adhesive groups significantly outperformed the ML‑NA group. These findings suggest that Piezo1, a mechanotransductive molecule, may play a pivotal role in mediating the mechanical stimulation‑driven immune responses. The findings align with previous observations that excessive motion at fracture sites impairs healing by disrupting vascular ingrowth and delaying the transition from pro‐inflammatory to reparative immune states [10, 11, 12]. Although RNA‐seq, flow cytometry, and immunofluorescence analyses have identified key regulatory pathways, rigorous mechanistic validation is still lacking. A comprehensive delineation of cell‐type‐specific responses and underlying mechanisms demands further in‐depth investigation, including single‐cell RNA sequencing, spatial transcriptomics, and genetic perturbation models. Collectively, these results imply an association whereby adhesive fixation of the ML‐AD scaffold suppresses micromotion, which in turn may contribute to the formation of a mechano‐immune microenvironment conducive to M2‑like macrophage polarization. The resulting M2 macrophage phenotype is associated with elevated VEGF‐ and BMP‐2‐mediated angiogenesis and osteogenesis, thereby promoting bone augmentation.

Overall, this study introduces adhesive‐mediated mechanical stabilization as a foundational design principle for bone tissue engineering scaffolds and provides both functional and mechanistic validation of its regenerative impact. Several limitations should nevertheless be acknowledged. First, validation was performed in small animal models with a relatively short experimental duration and an insufficiently large sample size; Future studies should be conducted in large animal models to better recapitulate the physiological conditions and mechanical loading microenvironment of human bones. Moreover, extending the experimental period and adopting larger sample sizes are required to evaluate long‐term bone regeneration outcomes and achieve more reliable statistical results. Further experimental design is therefore needed for subsequent verification in future work. Second, the bone adhesion test still cannot fully replicate the in vivo environment, which involves physiological temperature, enzymatic activity, and complex mechanical loading conditions. Finally, although macrophages play a central role in early regeneration, mechanical cues also regulate endothelial cells (excessive mechanical stimulation may directly cause mechanical damage to newly sprouted blood vessels), mesenchymal stem cells, and other immune populations [13, 60]. Future work will therefore focus on integrating multiscale mechanobiological analyses, optimizing refined experimental design and characterization, and advancing translational validation to support clinical deployment of the scaffold.

## 4 Conclusion

Guided by the perspective that mechanical stability within the osteogenic niche serves as a key regulator of bone regeneration, we developed a multilayer adhesive scaffold (ML‐AD) integrating a barrier layer, an adhesive layer, and an osteogenic layer into a single 3D‐printed construct (Scheme 1). The scaffold enables rapid wet adhesion to bone defects via simple finger pressing, providing immediate and robust fixation without external hardware, and thereby contributing to early mechanical stabilization of the regenerative niche. In rabbit mandibular and tibial augmentation models, the ML‐AD scaffold significantly enhanced bone augmentation compared with non‐adhesive scaffolds and even outperformed the specific GBR‐like comparator used in this study. Mechanistic investigations suggest an association whereby adhesive fixation correlates with reduced scaffold micromotion, which may facilitate the formation of a favorable mechano‐immune microenvironment. This microenvironment is accompanied by macrophage recruitment and M2‐like polarization, elevated VEGF and BMP‐2 secretion, as well as enhanced angiogenic and osteogenic differentiation, collectively contributing to improved bone augmentation. By highlighting interface‐fixation‐mediated mechanical stabilization of the osteogenic niche, this work provides a novel framework for facilitating bone augmentation in dynamic and complex defect settings, offers insights into potential mechano‐immune cues for mechanical‐stability‐secured bone augmentation, and demonstrates the promising potential of the scaffold in bone repair.

## 5 Materials and Methods

### 5.1 Materials

Gelatin was purchased from Sigma (USA); *N*, *N*‐dimethylformamide and sodium hydroxide were obtained from Fuyu Chemical Co., Ltd.(China); methacrylic anhydride was supplied by Aladdin (China); 2‐methacryloyloxyethyl phosphorylcholine and poly(ethylene glycol) diacrylate (PEGDA, average molecular weight ∼1000) were acquired from Aladdin (China); lithium phenyl‐2,4,6‐trimethylbenzoylphosphinate was purchased from Yuanye Bio‐Technology Co., Ltd. (China); dopamine (DA), sodium periodate (NaIO_4_), acrylic acid (AA), rhodamine, iohexol, and 2‐methacryloyloxyethyl phosphorylcholine were purchased from Aladdin (China); nanohydroxyapatite (nHAp, 60–80 nm) was obtained from Macklin Biochemical Co., Ltd. (China); Laponite was purchased from BYK (Germany). Additional materials are available in Section S1.1 or noted in the specific experimental procedures.

### 5.2 Cell Preparation

BMSCs and rat gingival fibroblasts were isolated and identified as detailed in Section S1.3.

### 5.3 GelMA Preparation

Detailed procedures for GelMA preparation are provided in Section S1.4.

### 5.4 Formulation Optimization and Characterization of Each Layer of ML‐AD Scaffold

The scaffold consisted of three structural layers: the barrier layer, adhesive layer, and osteogenic layer. The barrier, adhesive, and osteogenic layers were designed for anti‐protein or ‐cell adhesive performance, adhesive properties, and enhanced osteogenic differentiation capacity, respectively; their optimal formulations were screened for peak performance and then used for subsequent scaffold fabrication. The physicochemical properties of the optimized hydrogels were characterized. All detailed procedures are provided in Section S1.5.

### 5.5 Fabrication for ML‐AD Scaffold

The scaffold was fabricated in two sequential steps. The adhesive layer (the adhesive layer formulated as 30% AA, 0.5% GelMA, 10% Gelatin, and 0.5% DA) was first printed via DLP 3D printing with parameters: layer thickness = 1 mm, light intensity = 20 mW/cm^2^, and exposure time = 150 s. Post‐printing, the adhesive hydrogel was air‐dried in a sterile biosafety cabinet for 12 h and trimmed into an annular structure using a hole puncher. Then, the barrier and osteogenic layers (the barrier layer formulated as 9% PEGDA, 1% GelMA, and 20% MPC, and the osteogenic layer formulated as 15% GelMA, 6% nHAp, and 1% Laponite) were integrally printed via multi‐material mode with layer‐specific parameters: Barrier layer: layer thickness = 100 µm, light intensity = 20 mW/cm^2^, exposure time = 15 s, temperature = 45°C; Osteogenic layer: layer thickness = 100 µm, light intensity = 18 mW/cm^2^, exposure time = 5 s, temperature = 45°C. Finally, the pre‐fabricated annular adhesive layer was assembled to the integrated membrane‐porous structure to obtain the final scaffold.

### 5.6 Characterization of the Scaffold

#### 5.6.1 SEM

The structural morphology of the scaffold was observed using a SEM (Phenom ProX, The Netherlands) at an accelerating voltage of 10 kV. Meanwhile, EDS analysis was performed to determine the elemental composition at selected regions.

#### 5.6.2 Pore Morphology of the Osteogenic Layer of the Scaffold

For more intuitive observation of the porous structure, rhodamine (final concentration: 0.5 µg/mL) was added to the osteogenic layer slurry during printing. After printing, the pore morphology was observed using a laser confocal scanning microscope (LCSM, OLYMPUS, FV3000, Japan).

#### 5.6.3 The Precision Testing

The barrier and osteogenic layers were printed using their respective parameters (barrier: 100 µm thickness, 20 mW/cm^2^, 15 s; osteogenic: 100 µm thickness, 18 mW/cm^2^, 5 s). A 3D digital high‐definition microscope (KENYENCE VHX‐7000, Japan) was used to measure pore diameter (major/minor axes), perimeter, area, and overall diameter/height of the porous layer, as well as the diameter and thickness of the barrier layer (*N* = 3). For 3D precision, Iohexol contrast agent (final concentration: 300 mg/mL) was added to the inks. Printed scaffolds were scanned by Micro‐CT (Quantum GX, 90 kV, 88 µA, PerkinElmer, USA), reconstructed, and analyzed using Geomagic Qualify software.

#### 5.6.4 Mechanical Properties

Compressive mechanical tests were conducted to evaluate the mechanical performance of the Scaffold using a universal testing machine (CMT‐1000, SUST, China). All tests were performed at a speed of 10 mm/min at RT. Compressive modulus was calculated from the stress–strain curves (*N* = 3).

#### 5.6.5 Finite Element Analysis

Finite element analysis (FEA) was performed to analyze the stress distribution of the scaffold. A scaffold model was constructed using Magics software, and mechanical simulations were conducted using Abaqus software. Forces of 1 or 3 N were applied at 45°, horizontally, and vertically downward to observe the stress distribution.

#### 5.6.6 In Vitro Swelling and Degradation

The fabricated scaffold was freeze‐dried, weighed, and sterilized by EO gas. In vitro swelling and degradation tests (the degradation test used a solution containing 1 U/mL of type II collagenase, EFL‐ColII‐DE‐001) are provided in Section S1.7. (*N* = 3).

#### 5.6.7 In Vivo Degradation

In vivo degradation was evaluated in New Zealand rabbits (2.5–3 kg, purchased from Jinan Jinfeng Experimental Animal Co., Ltd), with approval from the Medical Ethical Committee of Stomatological Hospital affiliated with Shandong University (NO. 20240804). The scaffold and individual layer hydrogels were freeze‐dried and weighed (*W*_x_) prior to implantation. Anesthesia was induced by intravenous injection of a 1:1 mixture of Zoletil 50 and Xylazine (0.1 mL/kg), combined with local anesthesia using lidocaine. The scaffold and hydrogels were implanted subcutaneously in the rabbit back. At predetermined time points (1, 2, and 3 months post‐surgery), the rabbits were euthanized, and the implanted materials were retrieved, freeze‐dried, and weighed (*W*_y_). Histological analysis of the surrounding tissues was performed using HE staining (*N* = 3). The in vivo degradation rate was calculated using the formula:

$$\mathrm{Degradation}\;\mathrm{rate}\left(\%\right)=\left[\left(W_{x}-W_{y}\right)/W_{x}\right]\times 100\%\cdot$$

#### 5.6.8 Feasibility of Personalized Scaffold Fabrication

Rabbit heads and tibias were purchased commercially, and bone defects of various shapes (circular, square, triangular, and irregular) were created in the tibia, maxilla, and skull. The defect sites were scanned by micro‐CT and reconstructed. Personalized scaffolds were designed using Magics software, fabricated, and then fitted to the bone defects to verify the matching feasibility.

### 5.7 Bone Adhesion Performance of the Scaffold

#### 5.7.1 In Vitro Bone Adhesion Performance

For in vitro bone adhesion performance of the scaffold's adhesive layer, following optimized bonding conditions of the adhesive layer hydrogel (the detailed experimental methods are provided in Section S1.8), bone adhesion strength was tested under conditions simulating the presence of blood or SBF using the optimized parameters (8 N compressive force, 60 s duration). Key adhesion metrics of the scaffold's adhesive layer, including shear, tensile, and peel strength, as well as burst pressure, were evaluated. The shear, tensile, and peel strength tests were conducted at a crosshead speed of 50 mm/min, while the burst test was performed at a gas inlet rate of 9.7 mL/s. The maximum values of each indicator were recorded and statistically analyzed. The non‐adhesive layer hydrogel was prepared by prolonging the UV irradiation time of the adhesive layer hydrogel and served as the control group (*N* = 3).

The burst test was employed to assess the bone adhesion performance of the scaffold, given its ability to characterize the bonding strength at the bone defect site. For the burst pressure assay of the scaffold, a 6 mm‐diameter defect was created on rabbit tibial plateaus. The sterilized scaffold (prepared under sterile conditions and immersed and washed with 75% ethanol for sterilization) was bonded under conditions (in the presence of SBF or blood), and the maximum burst pressure was measured (a gas inlet rate of 9.7 mL/s, *N* = 3). In vitro long‐term adhesion test: a one‐week in vitro adhesion assay was performed to simulate the in vivo moist environment (only humidity was mimicked, while temperature was not, considering that high temperature would lead to tissue corruption). The scaffold was adhered to the bone defect, wrapped with muscle tissue to maintain moist conditions, and stored at 4°C. The scaffold was rehydrated with SBF daily. Burst pressure tests were conducted at predetermined time points (2 min, 30 min, 12 h, 24 h, 5 days, 7 days) to assess the long‐term adhesion strength (a gas inlet rate of 9.7 mL/s, *N* = 3).

#### 5.7.2 Finite Element Analysis

To further investigate the stress and displacement of the scaffold under in vivo loading conditions, a sophisticated model was established in which the scaffold was adhered to a rabbit mandibular defect, thereby simulating its in vivo bonding and fixation capability. Experimental details are provided in Section S1.9.

#### 5.7.3 In Vivo Adhesion Fixation Evaluation

New Zealand rabbits (2.5–3 kg) were purchased from Jinan Jinfeng Experimental Animal Co., Ltd, with approval from the Medical Ethical Committee of Stomatological Hospital affiliated with Shandong University (NO. 20240804). Iohexol (300 mg/mL) was added to the porous layer inks for visualization. The scaffold dimensions were as follows: osteogenic layer (diameter: 6 mm, thickness: 2 mm), barrier layer (diameter: 12 mm, thickness: 0.8 mm), and adhesive layer (annular structure with inner diameter: 8 mm, outer diameter: 12 mm). Anesthesia was induced by intravenous injection of a 1:1 mixture of Zoletil 50 and Xylazine (0.1 mL/kg), combined with local anesthesia using lidocaine. A circular defect (diameter: 6 mm, depth: 0.5 mm) was created on the tibial plateau or mandible. Five nutrient holes were drilled in the tibial plateau defect area. Multilayer adhesives scaffold (ML‐AD, a three‐layer construct consisting of an osteogenic layer, an adhesive layer, and a barrier layer) was implanted and fixed by pressing, while multilayer non‐adhesive scaffold (ML‐NA group: Irradiate the adhesive layer with a UV for 8 min to eliminate bonding properties; without the application of any compression during placement) and single scaffold (SS, a single‐layer construct composed solely of the osteogenic layer) served as controls. Detailed group information is summarized in Table S4. The rabbits were fed normally post‐surgery and subjected to 30 min of running on a small animal treadmill daily at a speed of 3 km/h. The scaffold fixation was evaluated by B‐mode ultrasound under anesthesia at 3 and 7 days post‐surgery. At 7 days post‐surgery, rabbits were euthanized and dissected. Micro‐CT scanning was performed to analyze the scaffold displacement (*N* = 4).

### 5.8 Biocompatibility, Cell Barrier Effect and Bioactivity of the Scaffold

#### 5.8.1 Biocompatibility

The biocompatibility of the scaffolds was evaluated (*N* = 3), including cytocompatibility, cytotoxicity of scaffold degradation products, and hemocompatibility. Detailed experimental procedures are provided in Section S1.10.

#### 5.8.2 Cell Barrier Effect

The cell isolation effect of the scaffold was assessed in vitro (*N* = 3), with detailed experiments provided in Section S1.11.

#### 5.8.3 Bioactivity

The in vitro performance of the scaffold was assessed for cell migration and osteogenic differentiation potential using ALP staining, ALP activity assay, RT‐qPCR, and immunofluorescence staining of osteogenic marker proteins (*N* = 3), with detailed experimental procedures provided in Section S1.12.

### 5.9 Bone Augmentation Study in Rabbits

#### 5.9.1 Surgery and Postoperative Management

This study was approved by the Medical Ethical Committee of Stomatological Hospital affiliated with Shandong University (NO. 20240804). New Zealand rabbits (2.5–3 kg, male, 4 months old, purchased from Jinan Jinfeng Experimental Animal Co., Ltd.) were randomly divided into 6 groups with 4 rabbits per group per time point; detailed group information is summarized in Table S4. The scaffold dimensions, anesthesia method, surgical site, and bone defect size were consistent with those described above for Section 5.7.3. The ML‐AD scaffold (ML‐AD group) was implanted and fixed by pressing (about 60 s). ML‐NA (ML‐NA group: irradiate the adhesive layer with UV for 8 min to eliminate bonding properties; without the application of any compression during placement) and SS groups were directly implanted as controls. For the CM‐BP group, bone powder was shaped into a cylinder (height: 1.5 mm, diameter: 6 mm) at the defect site, covered with a collagen membrane, and sutured. For the CM‐BP‐P group, titanium pins were used to fix the membrane in addition to the same procedures as the CM‐BP group. The implantation time for each material (CM‑BP, CM‑BP‑P, and ML‑AD) was measured in a blinded manner by a single operator, recorded from the start of placement to completion (before suturing), with *N* = 4 per group. Each rabbit contained both a mandibular defect site and a tibial defect site. The rabbits were fed normally post‐surgery and subjected to 30 min of running on a small animal treadmill daily at a speed of 3 km/h. Then, the rabbits were euthanized and dissected at 1 and 2 months post‐surgery. The heart, liver, spleen, lung, and kidney of rabbits at 1 month post‐surgery were fixed with 4% paraformaldehyde for 48 h, followed by hematoxylin and eosin (HE) staining to evaluate the in vivo toxicity of the material.

#### 5.9.2 Micro‐CT

After fixation, samples were stored in ethanol. The mandible and tibia were scanned and analyzed by micro‐CT (Quantum GX, 90 kV, 88 µA, PerkinElmer, USA). (The total volume (TV) was defined as a cylinder with a diameter of 6 mm and a height of 3 mm, extending from the base of the defect to 1 mm above the upper surface of the porous layer of the scaffold). Quantitative micro‑CT analysis was conducted using CTAn software, assessing key parameters including bone volume fraction (BV/TV), bone augmentation height (defined as the distance from the host bone surface to the apex of the newly formed bone), bone mineral density (BMD), trabecular thickness (Tb.Th), trabecular number (Tb.N), and trabecular separation (Tb.Sp) (*N* = 4).

#### 5.9.3 Histological Staining

For histological evaluation, bone tissue specimens were decalcified with 10% EDTA for 15 weeks, dehydrated in gradient ethanol, and embedded in paraffin. Serial 5 µm‐thick sections of the target area were prepared and stained with HE and Goldner's trichrome stain. Immunofluorescence staining for OCN (GeneTex, GTX13418, 1:300) and CD31 (Proteintech, 11265‐1‐AP, 1:500) was performed on tissues at 1 month post‐surgery. Immunohistochemical staining for OCN (GeneTex, GTX13418, 1:800) and BMP‐2 (Bioss, bs‐1012R, 1:300) was conducted on tissues at 2 months post‐surgery to evaluate new bone formation. Tartrate‐resistant acid phosphatase (TRAP) staining was used to assess bone remodeling in these regions. All staining procedures were performed according to the manufacturer's instructions. The height of bone augmentation, the area of new bone formation, and the positive rate of immunostaining were quantified using ImageJ software (*N* = 4). All tissue sections were randomly and uniformly sampled, and all histological observations and quantitative analyses were performed in a blinded manner to eliminate subjective bias.

### 5.10 Exploration of the Potential Mechanism in Rats

#### 5.10.1 Surgical Operation

Rats were selected instead of rabbits for the mechanistic studies, as research reagents (particularly antibodies) are more readily available in rats. The scaffolds (ML‐NA and ML‐AD) used in the rat tibial defect model had the following dimensions: osteogenic layer (diameter: 3.5 mm, height: 1.8 mm), barrier layer (8 × 5 × 0.5 mm), and adhesive layer (5 × 1 × 0.5 mm). The scaffolds were fabricated and assembled using a DLP 3D printer. Additionally, the adhesive layer of ML‐NA was rendered non‐adhesive by an additional 8 min of UV irradiation. ML‑NA and ML‑AD have identical compositions. This experiment was approved by the Medical Ethical Committee of Stomatological Hospital affiliated with Shandong University (NO. 20240804). Rats (Wistar, 8‐week‐old, purchased from Charles River) were divided into four groups: ML‐NA (non‐adhesive scaffold), ML‐AD (adhesive scaffold), ML‐NA+GsMTx4, and ML‐AD+GsMTx4. Rats were anesthetized with isoflurane gas. The local area of the tibial plateau was shaved and disinfected, and local anesthesia with lidocaine was administered to further reduce pain. A defect (diameter: 3.5 mm, depth: 0.5 mm) was created on the tibial plateau, five nutrient holes were drilled, and the scaffold was implanted. The ML‐AD scaffolds were securely placed via manual compression to achieve close bone contact, whereas the ML‐NA scaffolds were gently implanted without any mechanical compression. The rats were fed normally and allowed 30 min of out‐of‐cage activity daily for the first 7 days post‐surgery. Specifically, rats in the ML‑NA+GsMTx4 and ML‑AD+GsMTx4 groups received intraperitoneal injections of the Piezo1 inhibitor GsMTx‑4 (270 µg/kg; Abcam, USA) on alternate days. The ML‑NA and ML‑AD groups received physiological saline injections under the same schedule to serve as vehicle controls.

#### 5.10.2 In Vivo Micromotion Analysis by B‑Mode Ultrasound

At 3 and 7 days post‑surgery, rats were anesthetized with isoflurane and positioned to simulate hindlimb extension and retraction movements. B‑mode ultrasound videos were recorded during the movement cycles. The micromotion‑induced area of the scaffold was calculated by comparing two selected frames within one complete extension‑retraction cycle using ImageJ software (*N* = 3).

#### 5.10.3 Evaluation of Scaffold‑Promoted Bone Augmentation in Rat Tibia

At 1 month post‑surgery, the rats were euthanized, and the tibiae were harvested for Micro‑CT scanning and analysis. Bone augmentation efficacy was evaluated by measuring bone augmentation height and bone volume fraction (BV/TV) (*N* = 3).

#### 5.10.4 Transcriptome Sequencing

At 7 days post‐surgery, rats in ML‐NA and ML‐AD groups were euthanized, and samples were immediately collected, snap‐frozen in liquid nitrogen, and then used for RNA sequencing and data analysis (*N* = 3). The detailed experimental procedures were provided in Section S1.13.

#### 5.10.5 Flow Cytometry

At 7 and 14 days post‐surgery, ECM‐scaffold complexes (ML‐NA, ML‐AD, ML‑NA+GsMTx4 and ML‑AD+GsMTx4) were retrieved, and cells were isolated referring to the previous study [61, 62]. Subsequently, the cells were incubated with antibodies and analyzed by using the CytoFLEX SRT system (BECKMAN COULTER, USA) and FlowJo software (*N* = 3). The detailed experimental procedures are provided in Section S1.14.

#### 5.10.6 Immunofluorescence Staining

At 7 and 14 days post‐surgery, rats in the four groups were euthanized, and samples were dissected and fixed, followed by decalcification, embedding, and sectioning. Immunofluorescence staining was performed according to the antibody instructions: single staining was performed using the secondary antibody method, while double and triple staining were conducted using tyramide signal amplification (TSA) technology. All images were analyzed using ImageJ software. Staining targets included CD68, CD163, iNOS, VEGF, BMP‐2, CD31, EMCN, OSX, OPN, p‐NFκB p65, p‐STAT6 and Piezo1. Detailed antibody information and dilution ratios are summarized in Table S6 (*N* = 3).

### 5.11 Statistical Analysis

All statistical analyses of experimental data in this study were performed using GraphPad Prism 9.0, and the specific analytical strategies are as follows: (1) Comparison between two groups: Student's *t*‐test was used to evaluate the statistical significance of differences between two groups after passing the normality test and confirming a normal distribution. (2) One‐way/two‐way analysis of variance (ANOVA) was applied for multi‐group data comparison after passing the normality test and homogeneity of variance test, followed by Tukey's post hoc test. The type of ANOVA was selected based on data characteristics: one‐way ANOVA was adopted for single‐factor designs, while two‐way ANOVA was used for designs involving two or more factors. (3) Fisher's test was employed to compare the in vivo displacement differences between the two types of scaffolds. Significance criteria were defined as follows: ns (*p *≥ 0.05); * (*p *< 0.05); ** (*p *< 0.01); *** (*p *< 0.001); and **** (*p *< 0.0001). All quantitative data are expressed as mean ± standard deviation (SD). At least three biological replicates were set for all experiments (*N* ≥ 3) to ensure the reliability of data and the validity of statistical results.

## Author Contributions

D.L.Z.: Investigation, Methodology, Software, Data curation, Formal analysis, Validation, Visualization, Writing – original draft, Conceptualization. Q.Y.L.: Methodology, Software. L.Y.W.: Methodology, Software, Data curation. C.A.Z.: Methodology. Y.T.C.: Methodology. S.L.: Methodology. F.L.W.: Funding acquisition, Writing – review & editing, Project administration, Supervision, Resources. Z.Q.D.: Funding acquisition, Writing – review & editing, Project administration, Supervision, Resources.

## Conflicts of Interest

The authors declare no conflicts of interest.

## Supporting information




**Supporting File 1**: advs77028‐sup‐0001‐SuppMat.pdf.

**Supporting File 2**: advs77028‐sup‐0002‐VideoS1‐S12.zip.

## Data Availability

The data that support the findings of this study are available from the corresponding author upon reasonable request.
