# Supplementary material for: Bioadhesive‐Integrated 3D‐Printed Multilayer Scaffolds Secure a Mechanically‐Stable Osteoimmune Niche for Enhanced Bone Augmentation
Source: Adv Sci (Weinh). 2026 Aug 11:e77028. Online ahead of print. doi: 10.1002/advs.77028 (PMC13460421; doi:10.1002/advs.77028)
Supplement: Supplementary file 1 [file ADVS-9999-e77028-s001.pdf]

Bioadhesive-Integrated 3D-Printed Multilayer Scaffolds Secure a Mechanically-Stable Osteoimmune Niche for Enhanced Bone Augmentation

Delu Zhao[1], Qiuyi Li[1], Lingyun Wang[1], Chongao Zhang[1], Yutian Cheng[1], Shuang Liu[1], Fulan Wei[1], Zheqin Dong[1*]*

1 Department of Orthodontics & Additive Manufacturing, School and Hospital of Stomatology, Cheeloo College of Medicine, Shandong University & Shandong Key Laboratory of Oral Diseases & Shandong Engineering Research Center of Dental Materials and Oral Tissue Regeneration & Shandong Provincial Clinical Research Center for Oral Diseases, Jinan, Shandong, 250012, China

*** Correspondence**: Fulan Wei (weifl@email.sdu.edu.cn), Zheqin Dong (zheqindong@sdu.edu.cn)

This PDF file includes:

1. Supplementary Experimental Section.
2. Figures S1 to S63.
3. Tables S1 to S6.
4. Videos S1 to S12.
5. References.

Table of Contents

Supplementary Experimental Section......S-5

Supporting Figures......S-18

Figure S1......S-18

Figure S2......S-19

Figure S3......S-20

Figure S4......S-21

Figure S5......S-22

Figure S6......S-22

Figure S7......S-23

Figure S8......S-24

Figure S9......S-25

Figure S10......S-26

Figure S11......S-27

Figure S12......S-28

Figure S13......S-29

Figure S14......S-30

Figure S15......S-31

Figure S16......S-32

Figure S17......S-32

Figure S18......S-33

Figure S19......S-34

Figure S20......S-35

Figure S21......S-35

Figure S22......S-36

Figure S23......S-37

Figure S24......S-38

Figure S25......S-39

Figure S26......S-39

Figure S27......S-40

Figure S28....................S-41

Figure S29....................S-42

Figure S30....................S-43

Figure S31....................S-44

Figure S32....................S-45

Figure S33....................S-46

Figure S34....................S-47

Figure S35....................S-48

Figure S36....................S-49

Figure S37....................S-50

Figure S38....................S-51

Figure S39....................S-52

Figure S40....................S-53

Figure S41....................S-54

Figure S42....................S-54

Figure S43....................S-55

Figure S44....................S-55

Figure S45....................S-56

Figure S46....................S-57

Figure S47....................S-58

Figure S48....................S-58

Figure S49....................S-59

Figure S50....................S-59

Figure S51....................S-60

Figure S52....................S-61

Figure S53....................S-62

Figure S54....................S-62

Figure S55....................S-63

Figure S56....................S-64

Figure S57....……………………………………………………………..............................…S-64

Figure S58....……………………………………………………………..............................…S-65

Figure S59....……………………………………………………………..............................…S-65

Figure S60....……………………………………………………………..............................…S-66

Figure S61....……………………………………………………………..............................…S-66

Figure S62....……………………………………………………………..............................…S-67

Figure S63....……………………………………………………………..............................…S-67

Supporting Tables..S-68

Supporting videos..S-70

References..……………………………………………………………...........................…S-71

1. Supplementary Experimental Section

1.1 Materials

Fetal bovine serum (FBS), α-minimum essential medium (α-MEM), and penicillin-streptomycin (double antibodies) were purchased from Gibco (China); Calcein-AM/PI Kit, alkaline phosphatase (ALP) staining kit, ALP activity assay kit, and deoxyribonuclease I (DNase I) were obtained from Beyotime Biotechnology (China); Cell Counting Kit-8 (CCK-8) was purchased from Biosharp; bovine serum albumin (BSA), BSA-FITC, simulated body fluid (SBF), and rhodamine-conjugated phalloidin were acquired from Solarbio Science & Technology Co., Ltd. (China); Von Kossa staining kit and Alizarin Red S (ARS) were purchased from Wuhan Servicebio Technology Co., Ltd. (China); Zoletil 50 and Xylazine were obtained from Suzhou Kunchen Biotechnology Co., Ltd.; bone powder was purchased from Geistlich (Switzerland); collagen membrane was acquired from ZH-BIO Co., Ltd. (China); dispase II was purchased from Roche (Switzerland); collagenase type IV was obtained from Thermo Fisher Scientific (USA); and the FlexAble Kit was supplied by Wuhan Proteintech (China). Other reagents and materials are specified in the corresponding experimental procedures.

1.2 Basic elements of experimental design

To ensure the reliability and objectivity of experimental results, strict quality control measures were implemented throughout the study: all animal experiments involving rats and rabbits adopted the principle of complete randomization for animal allocation, and the order of surgical operations was also determined by randomization to avoid artificial bias between groups; blind analysis was adopted throughout the process for imaging and histological detections, including Micro-CT scan quantification, histological staining, and immunohistochemical result analysis, where analysts were completely unaware of the experimental grouping information; the gating strategy for flow cytometry was preset and standardized, with detection and analysis strictly completed in accordance with preset procedures; in addition, clear inclusion criteria, exclusion criteria, and supplementary criteria were formulated before the experiment.

1.3 Cell preparation

1.3.1 BMSCs

The isolation of BMSCs was performed as previously described.[1] Briefly, two-week-old Wistar (Charles River, USA) rats were used as donors. BMSCs were isolated from the femoral bone marrow cavity and cultured in α-MEM supplemented with 10% fetal bovine serum (FBS) and 1% penicillin-streptomycin in a cell incubator. The cells were passaged until reaching passage 3, and BMSCs with good growth status were selected for subsequent experiments. Briefly, BMSCs were identified by flow cytometry via detection of the positive rates of $CD90^+$ (Biolegend, 206105) and $CD34^+$ cells (Invitrogen, 11-0341-85). The results showed that the proportion of $CD90^+$ cells was over 99%, whereas the percentage of $CD34^+$ cells was below 10%, indicating a high purity of BMSCs (**Figure S62**).

1.3.2 Rat gingival fibroblasts

For the isolation of rat gingival fibroblasts (R-GFs), two-week-old Wistar rats were used. Gingival tissues were excised under sterile conditions using sterile scissors, then minced into small fragments. The tissue fragments were thoroughly rinsed six times with PBS containing 3% penicillin-streptomycin to remove contaminants. Subsequently, the minced tissues were transferred to 6-well plates and subjected to enzymatic digestion using a solution containing 3 mg/mL type I collagenase and 4 mg/mL type II dispase. After digestion, the released cells were cultured in DMEM supplemented with 10% FBS and 1% penicillin-streptomycin. The cells were cultured until passage 3, and R-GFs exhibiting excellent growth vigor were chosen for subsequent experiments. Briefly, rat gingival fibroblasts (R-GFs) were characterized by flow cytometry by detecting the positive rates of $Vimentin^+$ (Proteintech, 10366-1-AP) and $Pan\text{-}Cytokeratin^+$ cells (Arigo, ARG56129). Flow cytometric results revealed that the proportion of $Vimentin^+$ cells reached 96%, while the fraction of $Pan\text{-}Cytokeratin^+$ cells was lower than 7%. These results confirmed the high purity of the isolated R-GFs (**Figure S63**).

1.4 GelMA preparation

Accurately weigh 4 g of gelatin and dissolve it in 200 mL of diH_2O. Stir the mixture at 40 °C until the gelatin is completely dissolved. Adjust the pH value of the solution to 7.4 by adding 1 M sodium hydroxide (NaOH) solution. Subsequently, 70 mL of N, N-dimethylformamide (DMF) was introduced to form a reaction interface. Then, 600 μL of methacrylic anhydride was slowly added dropwise into the gelatin solution in three aliquots, followed by continuous stirring at 40 °C for 3 hours to complete the reaction. The resultant reaction solution was poured into 1500 mL of absolute ethanol to induce precipitation. The white cotton-like precipitate was collected, and the residual absolute ethanol was squeezed out manually. After being sheared into small pieces, the precipitate was redissolved in 100 mL of diH_2O. The reconstituted solution was freeze-dried, and the degree of substitution (62.27%, **Figure S1**) of the final product was characterized by ^{1}H NMR spectroscopy (BRUKER, Germany).

1.5 Formulation optimization and characterization of each layer of ML-AD

1.5.1 The barrier layer

1.5.1.1 Preparation of the barrier layer hydrogels

Synthesis of the barrier layer hydrogels with different formulations (designated as M0, M10, M20, and M30) were fabricated as follows. Briefly, GelMA (crosslinking agent and regulation of degradation rate), 2-methacryloyloxyethyl phosphorylcholine (MPC), poly (ethylene glycol) diacrylate (PEGDA), and lithium phenyl-2,4,6-trimethylbenzoylphosphinate (LAP, final concentration: 0.1% w/v) were dissolved in deionized water via ultrasonication and stirring at 40 °C. The homogeneous precursor solution was pipetted into polytetrafluoroethylene (PTFE) molds (pore diameter: 10 mm, height: 4 mm) and photo-crosslinked under ultraviolet (UV) light (25 mW/cm^2, 405 nm) at room temperature for 4 min. Alternatively, the precursor solution was cast onto acrylic plates and photo-crosslinked into 1 mm-thick hydrogel sheets. These sheets were either punched into thin hydrogel discs of various diameters using a hole puncher or cast into standard tensile test specimens using dedicated molds. All resulting hydrogels were reserved for subsequent experimental analyses. The formulations of all hydrogel groups are summarized in **Table S1**.

1.5.1.2 Cell compatibility

The cell compatibility assay was performed as previously described. [1] Cylindrical hydrogels from each group were sterilized by immersion in 75% ethanol followed by UV irradiation overnight. After sterilization, the hydrogels were gently rinsed six times with PBS and aseptically transferred to 6-well plates. BMSCs were cultured the hydrogels at a density of 2×10^5 cells/well. After 2 days of co-culture, the cells were stained using the Calcein-AM/PI double-staining kit, and fluorescent images were captured via a fluorescence microscope (KENYENCE BC-X810, Japan). Meanwhile, the Cell Counting Kit-8 (CCK-8) assay was conducted to quantify cell activity. For the cell proliferation assay, BMSCs were seeded at the same density (2×10^4 cells/well), and Calcein-AM/PI staining and CCK-8 assays were performed at 1, 3, 5, and 7 days post-seeding. Detailed experimental procedures followed the manufacturers' instructions (N=3).

1.5.1.3 Anti-adhesion performance

The anti-protein adhesion assay was carried out as follows: Hydrogels from each group were fabricated into circular discs (diameter: 10 mm) and rinsed three times with deionized water. The discs were then immersed in protein solutions (FBS or BSA, 2 mg/mL) and incubated in a 37 °C cell incubator for 2 hours. After incubation, the hydrogels were removed and gently rinsed three times with deionized water to remove unbound proteins. Subsequently, each hydrogel disc was transferred to a tube containing 2 mL of deionized water, sonicated for 5 minutes to elute adsorbed proteins, and the resulting supernatant was collected. The protein concentration in the supernatant was determined using the BCA assay, and the protein adsorption amount was calculated accordingly. For visual and quantitative validation, BSA-FITC was used as the model protein. After adhesion, fluorescent images were captured using a fluorescence microscope (KENYENCE BC-X810, Japan), and the fluorescence intensity was analyzed to quantify protein adsorption using ImageJ software (N=3).

The hydrogel discs (diameter: 10 mm) were sterilized with 75% ethanol and placed in 12-well plates. BMSCs or R-GFs were seeded onto the hydrogels at a density of

2×10^5 cells/well and cultured for 24 hours. After incubation, BMSCs or R-GFs were stained with Calcein-AM/PI and rhodamine-conjugated phalloidin following the manufacturers' protocols. Stained cells were imaged via a fluorescence microscope (KENYENCE BC-X810, Japan), and the number of adherent cells was quantified using ImageJ software (N=3).

1.5.1.4 Fourier Transform Infrared Spectroscopy (FTIR)

M0 and M20 hydrogels were freeze-dried and then subjected to FTIR (ALPHAII, BRUER, Germany) to characterize their chemical compositions. The spectra were recorded over the wavenumber range of 4000-400 cm^{-1}.

1.5.1.5 SEM

M0 and M20 hydrogels were snap-frozen in liquid nitrogen and subsequently freeze-dried. Prior to imaging, the samples were sputter-coated with a thin layer of gold to improve conductivity. The microstructural morphology was observed using a SEM (Phenom ProX, The Netherlands) at an accelerating voltage of 10 kV. Meanwhile, EDS analysis was performed to determine the elemental composition of the hydrogels at selected regions.

1.5.1.6 Swelling testing

M0 and M20 hydrogels were freeze-dried and their dry weights (W_d) were measured. After sterilization via ethylene oxide (EO) gas, the hydrogels were immersed in sterile diH_2O, PBS, and simulated body fluid (SBF, pH 7.4) at 37 °C. At predetermined time intervals (1, 3, 6, 12, 24, 72h, and 5d, 7d and 14d), the swollen hydrogels were retrieved, blotted gently with filter paper to remove surface moisture, and their wet weights (W_s) were recorded. The swelling ratio (SR) was calculated using the formula: $SR= W_s/W_d$ (N=3).

1.5.1.7 Degradation behavior

Freeze-dried M0 and M20 hydrogels were weighed (dry mass: W_0) and then sterilized via EO gas. The sterilized hydrogels were immersed in sterile diH_2O, PBS, and SBF containing type II collagenase (1 U/ml, EFL-ColII-DE-001) and incubated at 37 °C in a humidified atmosphere. At specified time points (7, 14, 21, 28, 35, 42, 49

and 56 days), the hydrogels were retrieved, thoroughly washed with deionized water, freeze-dried, and their residual dry weights (W_n) were measured. The degradation rate (DR) was calculated using the formula: DR= $(W_0-W_n)/W_0 \times 100\%$ (N=3).

1.5.2 The adhesive layer

1.5.2.1 Preparation of the adhesive layer hydrogels

Adhesive layer hydrogels with different formulations (designated as D0, D0.1, D0.5, and D1) were fabricated as follows. Briefly, gelatin, GelMA, dopamine (DA), acrylic acid (AA), and LAP (final concentration: 0.1% w/v) were mixed at the ratios summarized in **Table S2**, with stirring in a water bath at 40 °C until homogeneous. And then $NaIO_4$ (final concentration: 0.05% w/v) was added. Initially, D0.5 hydrogel sheets (thickness: 1 mm) were prepared using a digital light processing (DLP) 3D printer (20 mW/cm², EFL-BP8601 Pro, China) under varying UV irradiation times (120–360 s) to optimize the exposure duration. After determining the optimal irradiation time (150s), sheet hydrogels of all groups were fabricated following the same protocol. The hydrogels were then air-dried at room temperature and cut into square specimens of various sizes. Alternatively, disc-shaped hydrogels were prepared using the dedicated molds (diameter: 10 mm, height: 4 mm). All hydrogel specimens were reserved for subsequent experiments.

1.5.2.2 Adhesive strength test

For the bone adhesion shear strength assay, 1 cm × 1 cm adhesive hydrogel specimens were placed between two bovine bone fragments (in the presence of PBS). A compressive force of approximately 8 N was applied for 60 seconds to ensure intimate contact. The bonded bone-hydrogel-bone constructs were then subjected to shear strength testing using a universal testing machine (CMT-1000, SUST, China) at a speed of 50 mm/min, and the shear strength was calculated from the test results (N=3).

1.5.2.3 Cytocompatibility

The cell compatibility of the adhesive hydrogels was evaluated following the same protocol as that used for the barrier hydrogels, including live/dead staining and CCK-8 assay (N=3).

1.5.2.4 Physical characterization

After optimizing the formulation, D0.5 and D0 hydrogels were selected for further characterizations, including FTIR spectroscopy, SEM coupled with EDS, swelling kinetics assays, and degradation assays. All tests were performed following the same procedures as those for the barrier hydrogels (N=3).

1.5.3 The osteogenic layer

1.5.3.1 Synthesis of the osteogenic layer gels

Osteogenic layer hydrogels with different formulations (designated as G15, N1, L0, L0.5, L1, and L2) were fabricated as follows. Detailed formulations are summarized in Table S3. Briefly, GelMA, nanohydroxyapatite (nHAp), and Laponite were mixed at the specified ratios and stirred at 40 °C until homogeneous. The precursor solution was either pipetted into polytetrafluoroethylene (PTFE) molds (pore diameter: 10 mm, height: 4 mm) and photo-crosslinked under ultraviolet (UV) light (25 mW/cm^2, 405 nm) at room temperature for 4 min. Alternatively, the precursor solution was cast onto acrylic plates and photo-crosslinked into 1 mm-thick hydrogel sheets. These sheets were either punched into thin hydrogel discs of various diameters using a hole puncher or cast into standard tensile test specimens using dedicated molds. All resulting hydrogels were reserved for subsequent experimental analyses.

1.5.3.2 Cytocompatibility

To evaluate the effects of osteogenic layer hydrogels on BMSC viability and proliferation, live/dead staining and the CCK-8 assay were performed following the same protocols as those for the barrier hydrogels

1.5.3.3 Osteogenic activity

Cylindrical hydrogels (diameter: 10 mm, height: 4 mm) were sterilized and placed in 6-well plates, followed by seeding with BMSCs at a density of 2×10^5 cells/well. After 7 days of co-culture, alkaline phosphatase (ALP) staining was conducted using an ALP staining kit, and ALP activity was quantified with an ALP activity assay kit. For the detection of osteogenic-related gene expression (RUNX2, ALP, OCN, and OPN), RT-qPCR was performed. Briefly, total mRNA was extracted using AG RNAex Pro

Reagent (Accurate Biology, China), and cDNA was synthesized using the Evo M-MLV RT Mix Kit (Accurate Biology, China). The cDNA was mixed with SYBR Green I (Accurate Biology, China) and osteogenic gene-specific primers (**Table S5**, Accurate Biology, China) according to the manufacturer's instructions. Gene expression levels were determined using a quantitative real-time PCR system (Light Cycler Roche 480 II, Roche, Swiss), and relative expression levels were calculated and normalized to GAPDH. After 14 days of culture, Von Kossa staining and Alizarin Red S (ARS) staining were performed following the respective kit protocols. For quantitative analysis of ARS staining, mineralized nodules were dissolved in cetylpyridinium chloride solution, and the absorbance was measured using a microplate reader (N=3).

1.5.3.4 Physicochemical characterization

After formulation optimization, the L1 hydrogel was selected for detailed characterizations, with G15/N1/L0 serving as the control group. Physicochemical properties, including SEM coupled with EDS, swelling kinetics assays, and degradation assays, were performed following the same procedures as those for the membranous hydrogels (N=3).

1.6 Performance of printing inks

1.6.1 Photosensitivity of printing inks

The photosensitivity of each layer's ink was also evaluated by monitoring the changes in viscoelastic properties of the ink upon UV irradiation using a rheometer (MCR 302, Anton Paar, Austria) with a parallel plate geometry (diameter: 25 mm, gap: 1 mm). The test parameters were set as follows: UV light (405 nm) with an intensity of 20 mW/cm^2, shear strain of 0.5%, angular frequency of 20 rad/s, temperature of 45 °C, and UV irradiation duration of 90 s for all groups.

1.6.2 Optimization of printing parameters

Printing precision tests were performed to optimize the printing parameters of M20 (barrier layer) and L1 (osteogenic layer). A printing precision test piece was designed, with a side length of 10 mm, thickness of 1 mm, and pore diameters ranging from 50 to 2400 μm (**Figure S19A**). The test pieces were printed under different parameter

combinations, and the printing precision was evaluated by measuring the actual dimensions of the printed samples. For barrier layer (M20), the slicing thickness was fixed at 100 μm, and the light intensity was set to 20 mW/cm². Four groups of samples were prepared with different layer UV exposure times (10, 12, 15, and 18 s), which were designated as M20/20-10, M20/20-12, M20/20-15, and M20/20-18, respectively. For osteogenic layer (L1), the slicing thickness was fixed at 100 μm, and the light intensity was set to 18 mW/cm². Four groups of samples were prepared with different layer UV exposure times (3, 4, 5, and 6 s), which were designated as L1/18-3, L1/18-4, L1/18-5, and L1/18-6, respectively.

After printing, a 3D digital high-definition microscope (KENYENCE VHX-7000, Japan) was used to capture images of the samples, and the actual dimensions (pore diameter) of the printed test pieces were measured to assess the printing precision (N=3).

1.7 In vitro swelling and degradation

The fabricated scaffold was freeze-dried, weighed, and sterilized by EO gas. In vitro swelling and degradation tests (the degradation test used a solution containing 1 U/mL of type II collagenase, EFL-ColII-DE-001) were performed following the same procedures as the barrier layer hydrogel tests (N=3).

1.8 Optimization bonding conditions

To enhance bone adhesion efficacy, the adhesive conditions of the adhesive layer hydrogel were optimized by varying compressive forces (2 N, 5 N, 8 N, and 12 N) and pressing durations (10 s, 30 s, 60 s, 120 s, and 180 s). Shear strength and burst pressure were measured to evaluate the adhesion performance. As shown in **Figure S26**, a custom-printed pressing tool was used to apply different forces and durations on a universal mechanics testing machine. The burst pressure test setup was assembled as illustrated in **Figure 3H**. The shear strength test was conducted at a crosshead speed of 50 mm/min, while the burst test was performed at a gas inlet rate of 9.7 mL/s. The optimal compressive force and pressing duration were determined by comparing the test results (N=3).

1.9 FEA of bone adhesion of the scaffold

To further investigate the stress and displacement of the scaffold under in vivo loading, a more complex model was established, which included the scaffold adhered to a rabbit mandibular defect, as well as the overlying skin and mucosa (**Figure 3L**). A dynamic obliquely upward force (gradually increasing over 0.6 s, with uniform force magnitude at each time point) was applied to the scaffold system. The stress distribution and displacement of each scaffold layer were compared with those of the non-adhesive scaffold group using Abaqus software. The material properties of each layer of the scaffold were defined as follows: for the barrier layer, an elastic modulus of 158.35 kPa and a Poisson's ratio of 0.436; for the adhesive layer, an elastic modulus of 16.75 kPa and a Poisson's ratio of 0.48; and for the osteogenic layer, an elastic modulus of 93.93 kPa and a Poisson's ratio of 0.452. The adhesive strength between the adhesive layer and the bone surface was determined as 69.61 kPa in shear. Briefly, all scaffold models were adaptively meshed in 3-matic using C3D4 tetrahedral elements, and the resulting meshes were exported as INP files and subsequently imported into Abaqus for finite element analysis. To simulate the outward displacement of the scaffold beyond the maxilla during mastication, a reference point was established on the membrane and kinematically coupled to it; an upward-anterior displacement was then imposed on this reference point to drive membrane-mediated outward migration of the scaffold, and the corresponding reaction force at the reference point was recorded. This reaction force, determined as 0.705 N vertically and 1.12159 N horizontally, was subsequently applied as the external load to both the bonded and non-bonded groups, with a linear ramp from zero to the full magnitude over a 0-to-0.6s interval. The distal end of the mandible was fully constrained in all six degrees of freedom as the boundary condition.

1.10 Biocompatibility of the scaffold

The cell compatibility of the scaffold was evaluated following the protocol used for the barrier layer hydrogel. Briefly, sterilized scaffold samples were co-cultured with BMSCs in 6-well plates at a cell density of 2×10^4 cells/well for 1, 3, 5, and 7 days. Similarly, RAW264.7 macrophages and HUVECs (2×10^5 cells/well) were also used to evaluate the biocompatibility of the scaffold. Live/dead staining and CCK-8 assay

were performed to assess cell viability (N=3).

To further investigate cell behavior on the scaffold, BMSCs were seeded onto the osteogenic layer of the scaffold at a density of 4×10^5 cells/scaffold. After 2 days of culture, CCK-8 assay, live/dead staining, and rhodamine-conjugated phalloidin staining were conducted to evaluate cell viability and observe the growth status of BMSCs on the scaffold (N=3).

Cell compatibility of degradation products from the scaffold and its individual hydrogel layers were also tested. Sterilized scaffold and hydrogel samples were placed in 50 mL centrifuge tubes, and culture medium was added. Degradation solutions collected at 5 days and 4 weeks were mixed with fresh culture medium at a 1:1 ratio, and the mixture was used to culture BMSCs. CCK-8 assay and Live/Dead staining were performed to characterize BMSCs viability (N=3).

The hemocompatibility of the scaffold and its individual hydrogel layers was evaluated using a hemolysis assay, following the method described in previous studies (N=3).[2]

1.11 Cell barrier effect of the scaffold barrier layer

The cell isolation effect of the scaffold was assessed according to previously reported protocols.[3] Sterilized scaffolds were placed in the inserts (as shown in **Figure S39A**) with the barrier layer facing upward. R-GFs and H-SFs (Human skin fibroblasts, STM-CL-5176) were seeded onto the barrier layer at a density of 2×10^5 cells/well. S-L1 scaffolds (without the barrier layer) served as the control group (N=3).

After 24 hours of culture, the inserts were removed, fixed, and stained with crystal violet. Images were captured using a microscope, and the number of cells was quantified (N=3).

1.12 Bioactivity of the scaffold

1.12.1 Cell migration assays

The sterilized scaffolds and their individual layer samples were placed in 50 mL centrifuge tubes containing α-MEM medium. According to GB/T 16886.12-2017 (Biological evaluation of medical devices), the ratio of hydrogel to medium was 0.1 g:1

mL.[4] The samples were incubated at 37 °C for 48 hours, and the extracts were collected. The extracts were used for cell migration assays, including Transwell migration assay and scratch wound healing assay, following the methods described in previous studies (N=3).[5]

1.12.2 Osteogenic differentiation potential

The osteogenic differentiation potential of the scaffold was evaluated in vitro following the protocol used for the osteogenic layer hydrogel. Briefly, BMSCs were co-cultured with the scaffold or its individual layer gel in 6-well plates, and relevant experiments were performed at 7, 14, and 21 days of culture. At 7 days: ALP staining, ALP activity assay, RT-qPCR (see **Table S5** for primer information), and immunofluorescence staining of osteogenic marker proteins (osteocalcin, OCN; osteopontin, OPN) were conducted (N=3). At 14 days: ARS staining, Von Kossa staining, and their quantitative analyses were performed (N=3). At 21 days: ARS staining and its quantitative analysis were carried out (N=3).

1.13 RNA sequencing

The samples were transported on dry ice to Cocmos Wisdom Co., Ltd. (Hangzhou, Zhejiang) for RNA sequencing and data analysis. The main workflow included total RNA extraction, quality control, mRNA enrichment using Oligo dT beads, mRNA fragmentation, cDNA synthesis, adaptor ligation, sequencing on the Illumina platform, and final data analysis and visualization (N=3). Briefly, based on the NovaSeq X Plus platform, the sequencing experiment adopted the Illumina NovaSeq Reagent Kit method for library construction. The alignment tool was HiSat2, and the reference genome version was Rattus Norvegicus mRatBN7.2 release-110. The gene expression level was calculated by the number of clean reads mapped to the genomic region (reads counts). The software RSEM was used to quantitatively analyze the expression levels of genes and transcripts respectively. The software used for differential expression analysis was DESeq2 (used by default for data with biological replicates) or DEGseq (used by default for data without biological replicates). The default screening criteria for significantly differentially expressed genes were: $P\text{-value}<0.05$ & $|\log2FC|>2$. A gene was considered a differentially expressed gene (DEG) when it met both criteria

simultaneously.

1.14 Flow cytometry test

Briefly, tissue blocks were minced and digested with dispase II (1 mg/mL), collagenase type IV (2 mg/mL), and DNase I (0.1 mg/mL). The cell suspension was filtered through a 40 μm strainer, centrifuged, and resuspended. The FlexAble kit (Proteintech) was used for fluorescent labeling of antibodies. Antibody incubation was performed according to the manufacturers' protocols using CD68 (BioLegend, 201003), CD86 (Proteintech, 65224-1-1G), and CD206 (Santa Cruz, sc-58986). Prior to data acquisition, the flow cytometer was calibrated using compensation beads. Unstained and single-stained samples were used to adjust fluorescence compensation and to define appropriate gating boundaries, thereby minimizing spectral overlap and ensuring reliable quantification. Flow cytometry analysis was performed using the CytoFLEX SRT system (BECKMAN COULTER, USA) and FlowJo software (N=3).

2 Supporting Figures

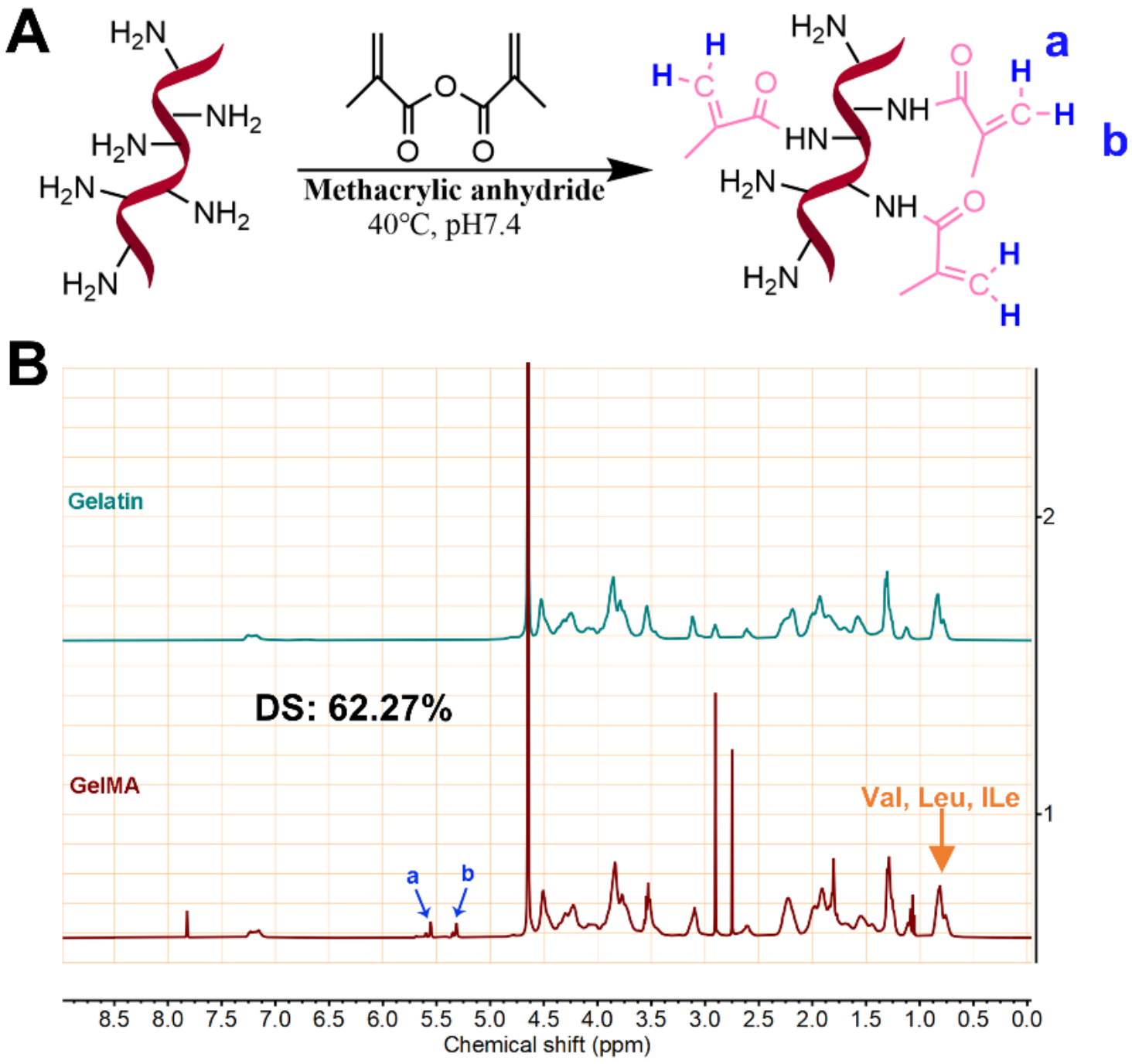

Figure S1. Preparation of GelMA. (A) Chemical reaction scheme for GelMA synthesis; (B) ^{1}H NMR spectrum of gelatin and GelMA. **a** and **b** represent the proton from the methacrylic anhydride; Degree of Substitution (DS) of GelMA:62.27%.

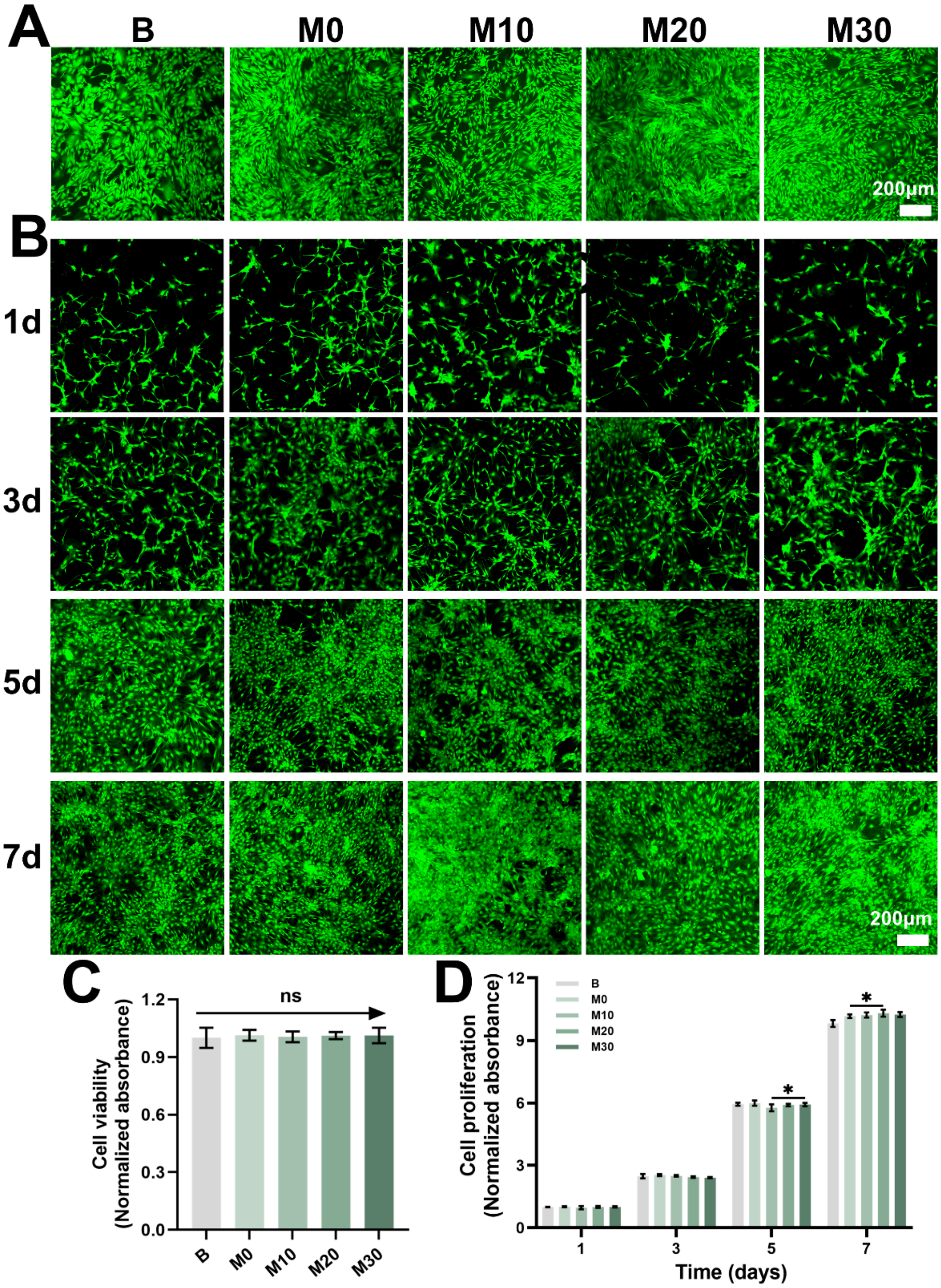

Figure S2. Cytocompatibility of the barrier layer hydrogels. (A) Representative fluorescence microscopy images of BMSCs stained with Calcein-AM/PI following 2 days of culture with various hydrogels. (B) Representative fluorescence microscopy images of BMSCs stained with Calcein-AM/PI following 1, 3, 5, and 7 days of culture with the hydrogels. Quantitative analysis of BMSC viability (C; statistical analysis was performed using one-way ANOVA) and proliferation (D; statistical analysis was performed using two-way ANOVA) determined by the CCK-8 assay. Scale bar = 200 μm; "ns" indicates no statistical significance ($P>0.05$); $^*P < 0.05$. N = 3.

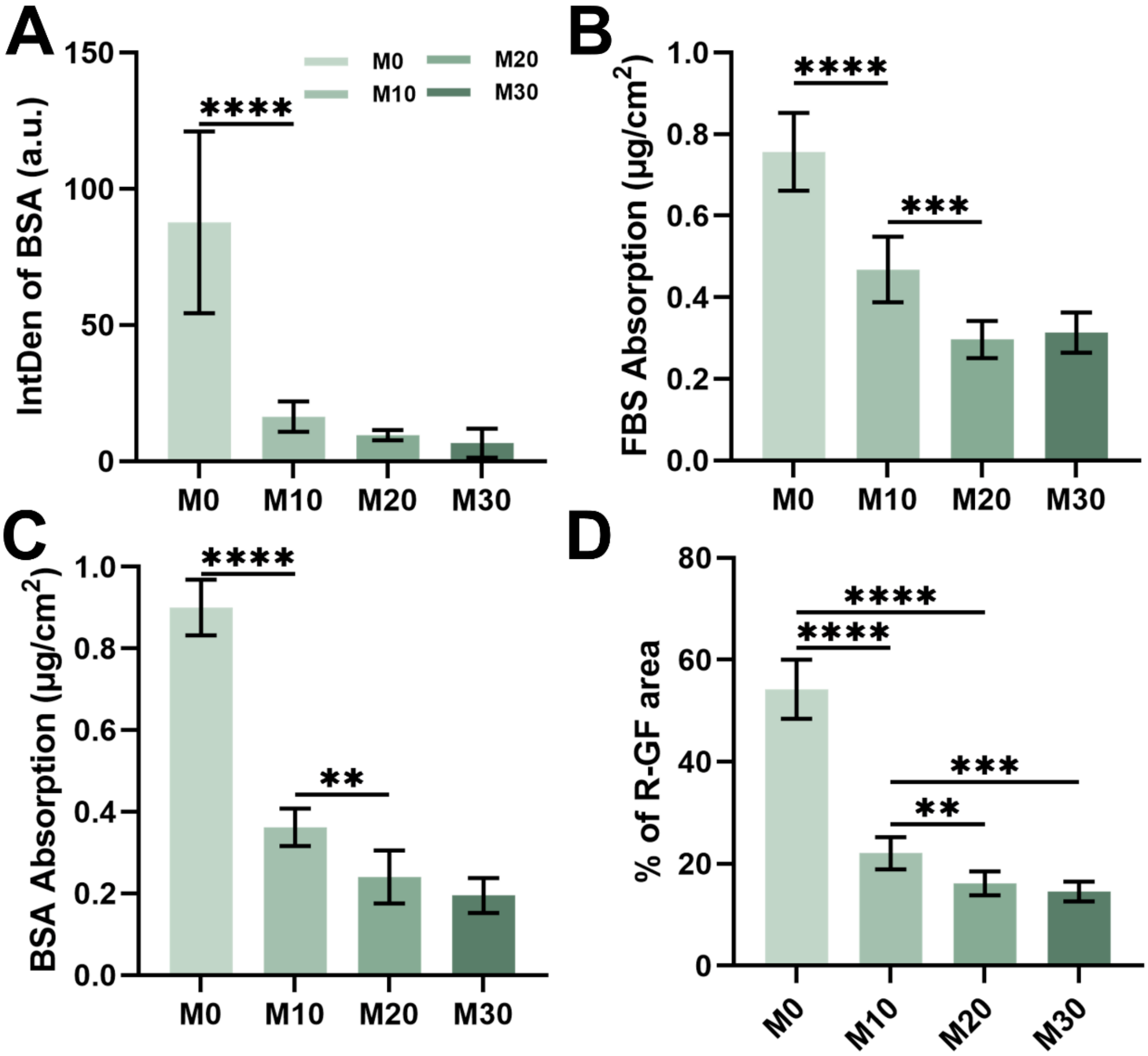

Figure S3. Quantitative evaluation of the anti-adhesive properties of barrier hydrogels. (A) Integrated density analysis of BSA-FITC adhered to barrier hydrogels. (B, C) Quantitative analysis of fetal bovine serum (FBS) (C) and bovine serum albumin (BSA) (B) and adhered to barrier hydrogels. (D) Quantitative analysis of the area ratio of R-GFs adhered to barrier hydrogels via Calcein-AM/PI staining. Statistical analysis in this figure was performed using one-way ANOVA. **P < 0.01; ***P < 0.001; ****P < 0.0001. N = 3.

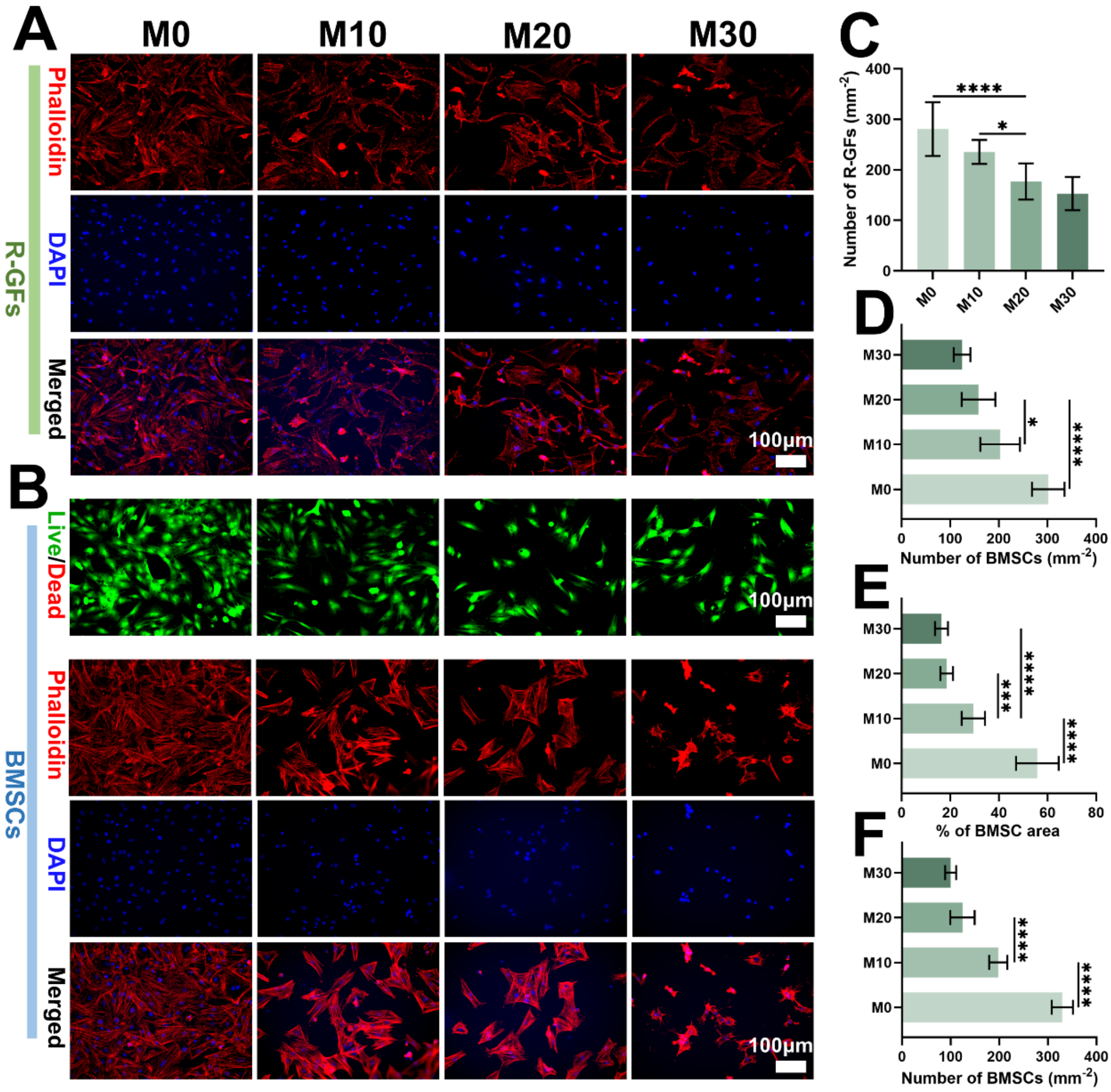

Figure S4. Anti-cell adhesion of barrier hydrogels. (A) Representative phalloidin-stained images of R-GFs adhered to barrier hydrogels. (B) Representative images of BMSCs adhered to barrier hydrogels stained with Calcein-AM/PI and phalloidin. (C) Quantitative analysis of the area ratio of R-GFs adhered to barrier hydrogels via phalloidin staining. (D-F) Quantitative analysis of BMSCs adhered to barrier hydrogels via Calcein-AM/PI (D, E) and phalloidin staining (F). Statistical analysis in this figure was performed using one-way ANOVA. *$P < 0.05$; ***$P < 0.001$; ****$P < 0.0001$. N = 3.

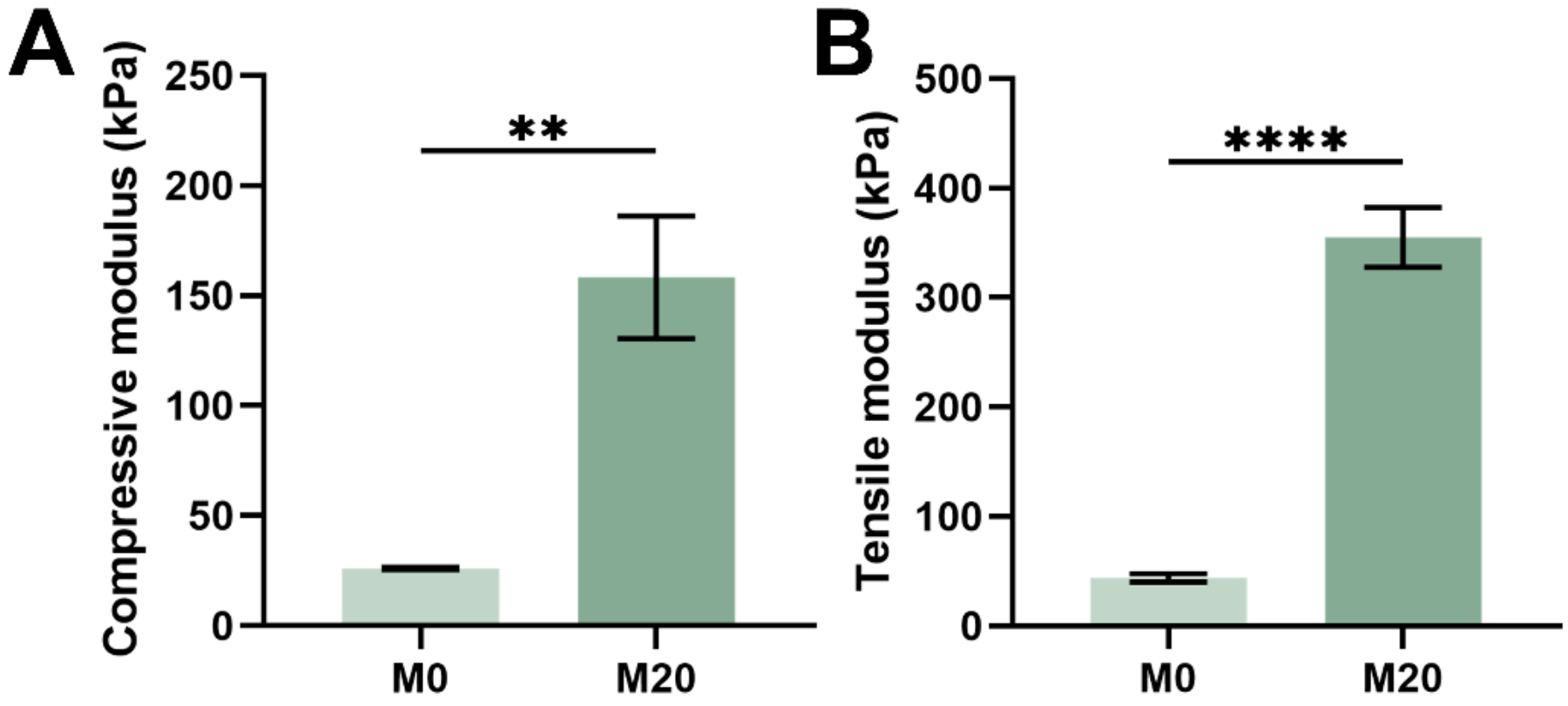

Figure S5. Mechanical properties of the barrier layer hydrogel (M20) and control group (M0). (A) Compressive modulus. (B) Tensile modulus. Unpaired Student's t-test was used for statistical analysis in this figure. $**P < 0.01$; $****P < 0.0001$. $N = 3$.

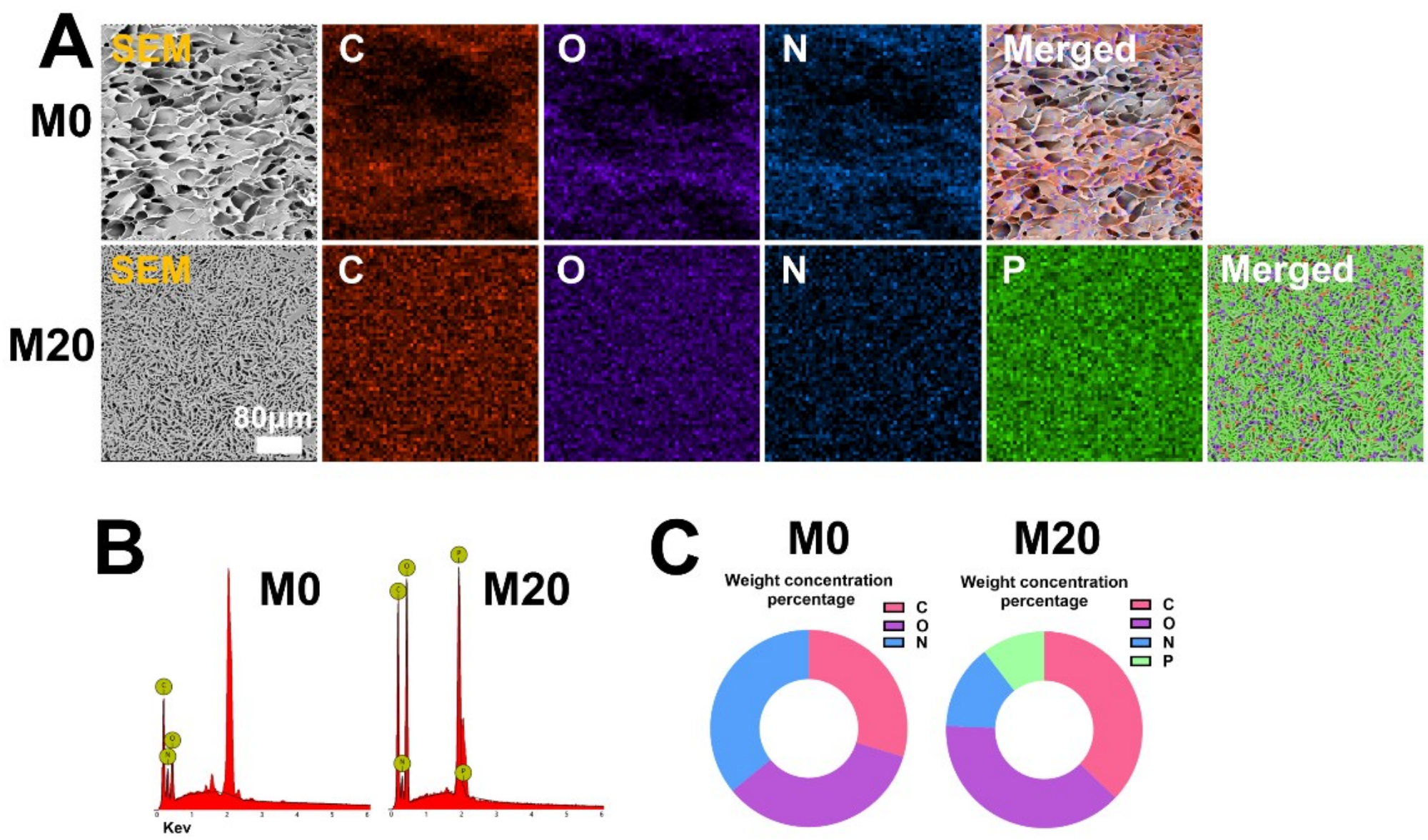

Figure S6. Morphology and elemental composition of the barrier hydrogels. (A) SEM and elemental mapping images of the barrier hydrogels. (B) EDS spectra of the barrier hydrogels. (C) Elemental composition and weight percentages of the barrier hydrogels.

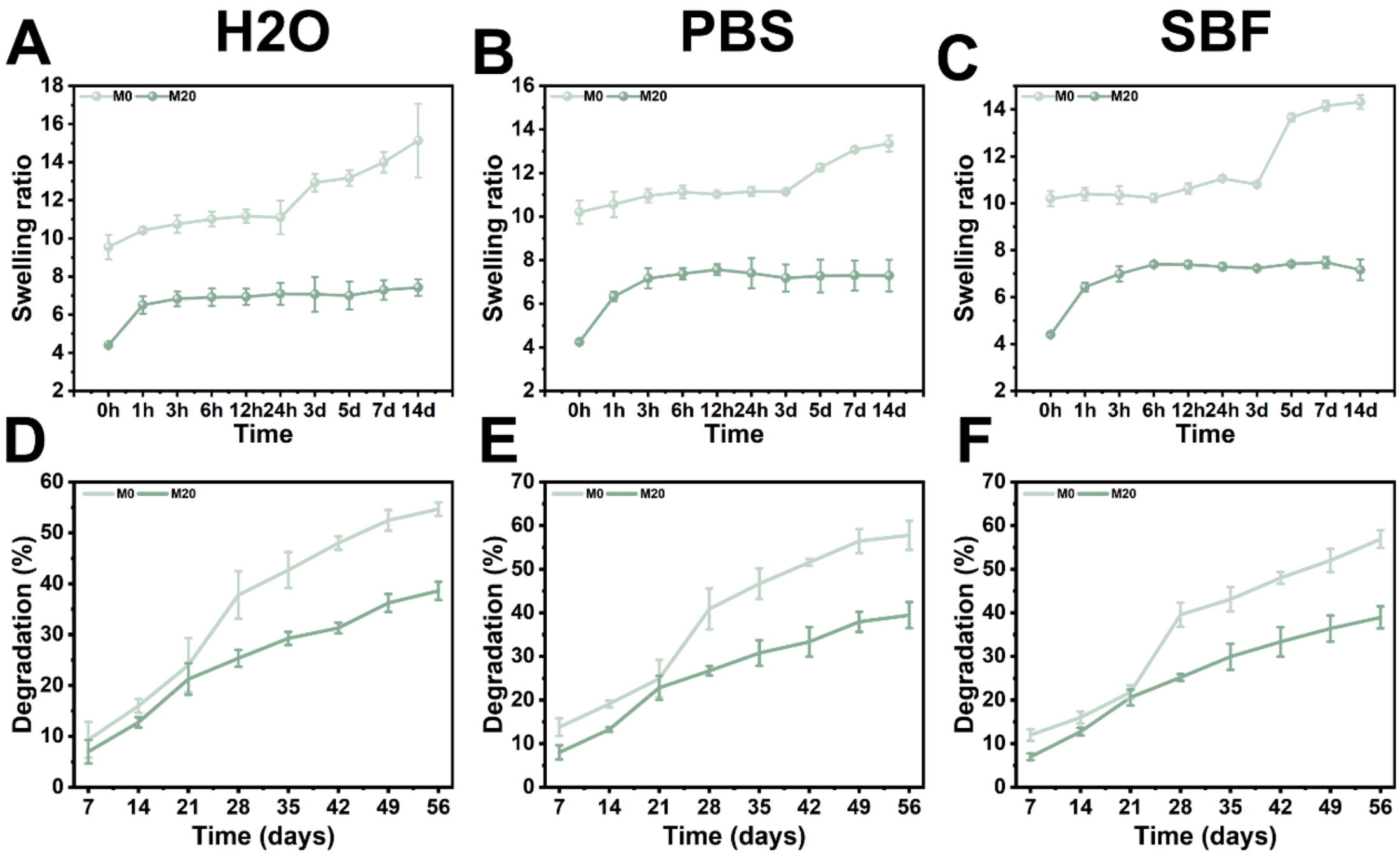

Figure S7. Swelling and degradation kinetics of the barrier hydrogels. (A-C) Swelling behaviors of barrier hydrogels in diH_2O(A), PBS(B), and SBF(C) over 2 weeks. (D-F) Degradation kinetics of barrier hydrogels in diH_2O(D), PBS(E), and SBF(F) over 8 weeks. N = 3.

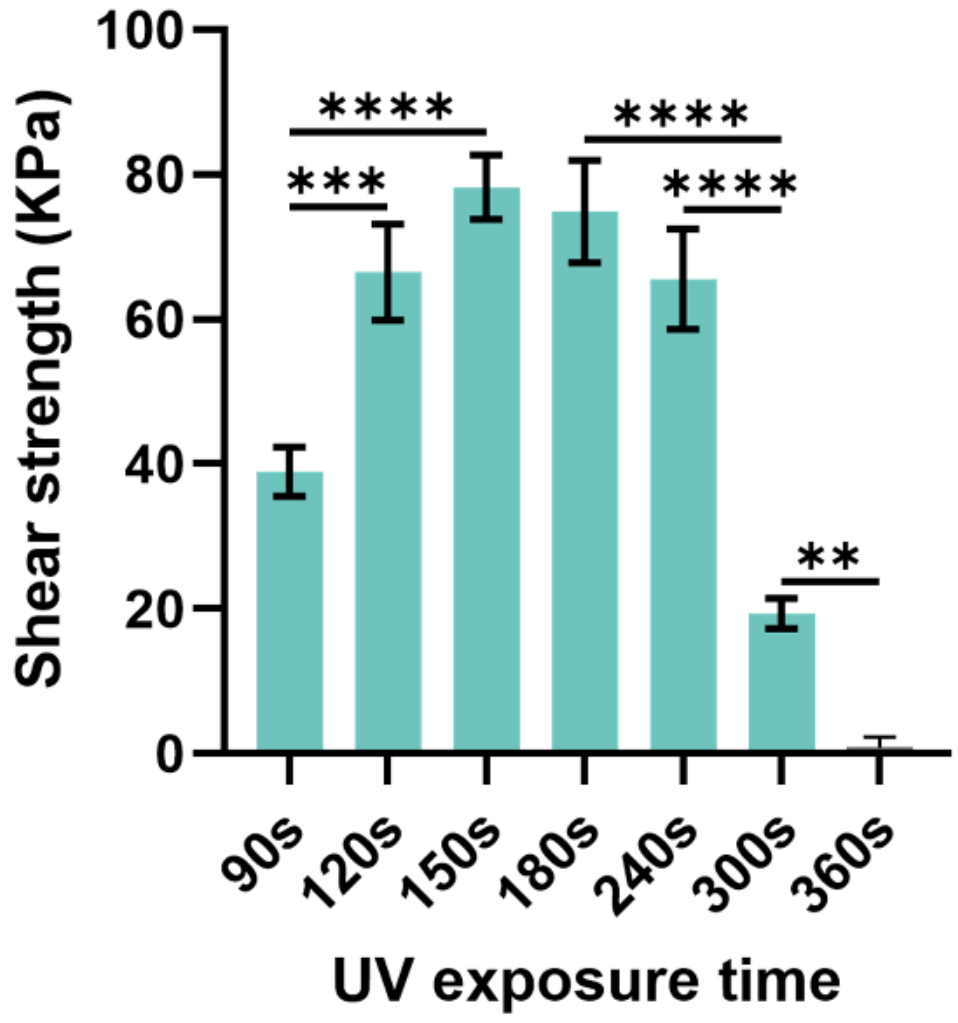

Figure S8. Bone adhesive shear strength of the adhesive layer hydrogel (D0.5) prepared with different UV exposure times. Statistical analysis in this figure was performed using one-way ANOVA. **P < 0.01; ***P < 0.001; ****P < 0.0001. N = 3.

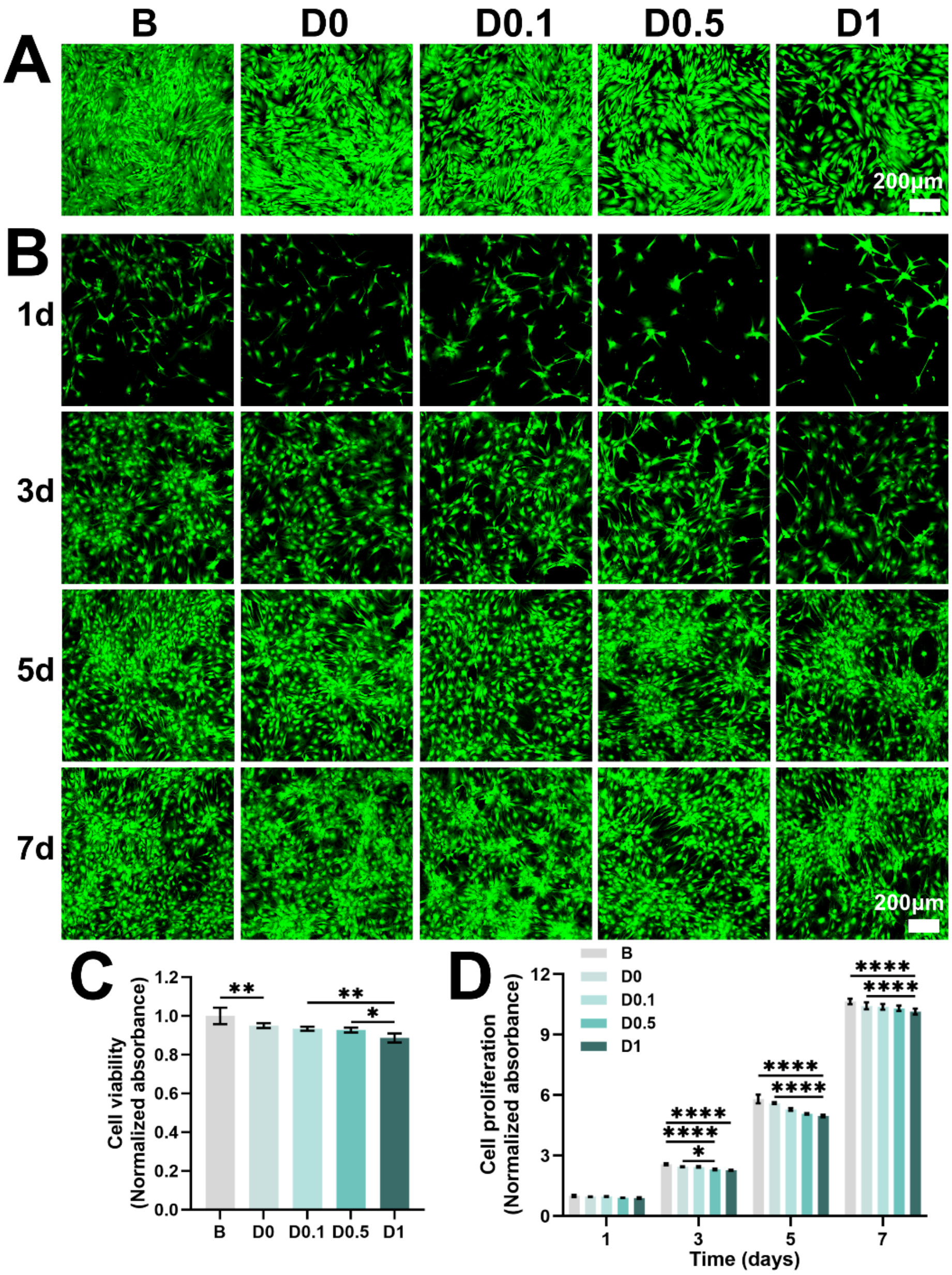

Figure S9. Cytocompatibility of the adhesive layer hydrogels. (A) Representative fluorescence microscopy images of BMSCs stained with Calcein-AM/PI after 2 days of culture with the hydrogels. (B) Representative fluorescence microscopy images of BMSCs stained with Calcein-AM/PI after 1, 3, 5, and 7 days of culture with the hydrogels. Quantitative analysis of BMSC viability (C; statistical analysis was performed using one-way ANOVA) and proliferation (D; statistical analysis was performed using two-way ANOVA) using the CCK-8 assay. Scale bar = 200 μm; *P < 0.05; **P < 0.01; ****P < 0.0001. N = 3.

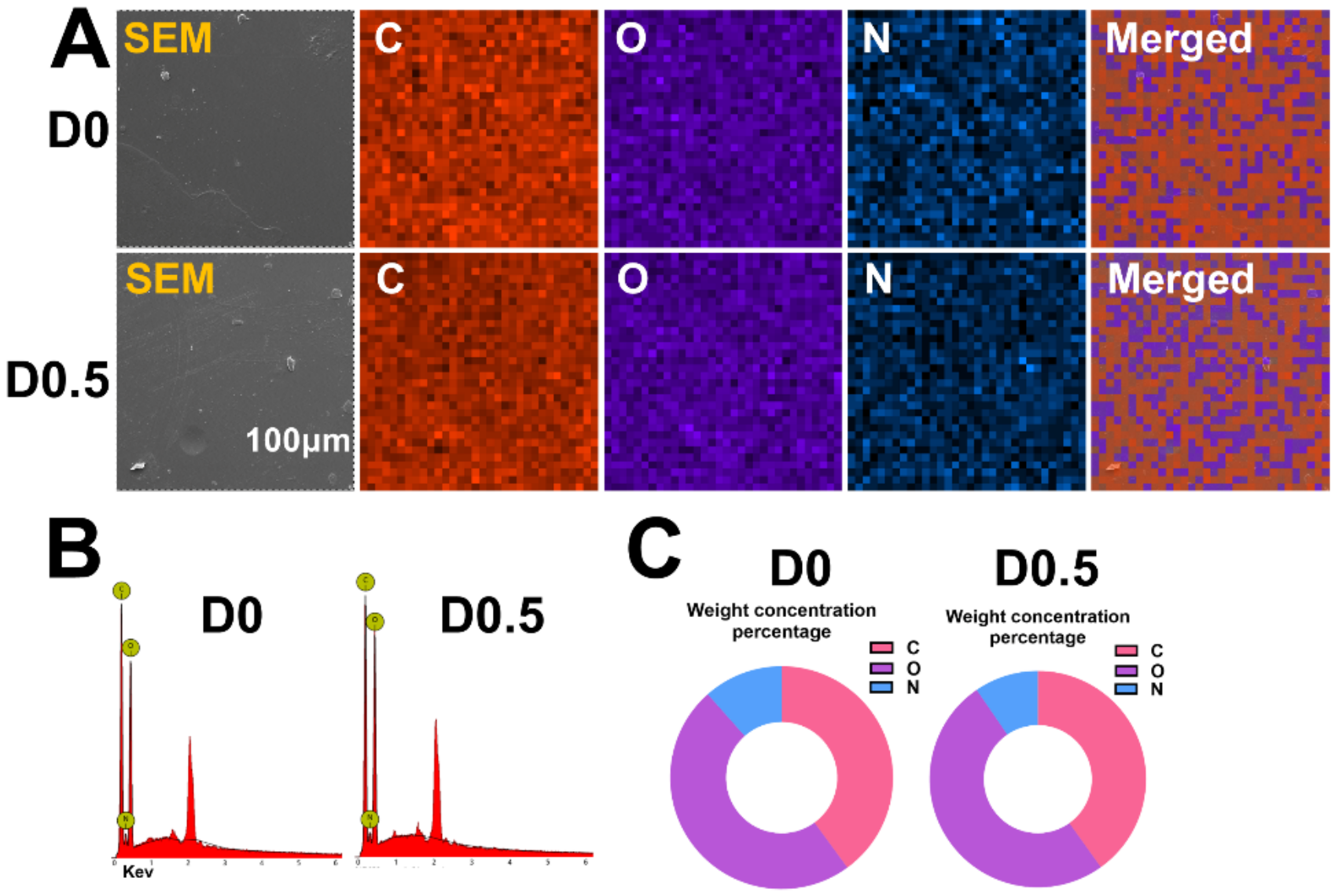

Figure S10. Morphology and elemental composition of the adhesive hydrogels. (A) SEM micrographs and corresponding elemental maps of the adhesive hydrogels. (B) Representative EDS spectra of the adhesive hydrogels. (C) Elemental composition and weight percentages of the adhesive hydrogels.

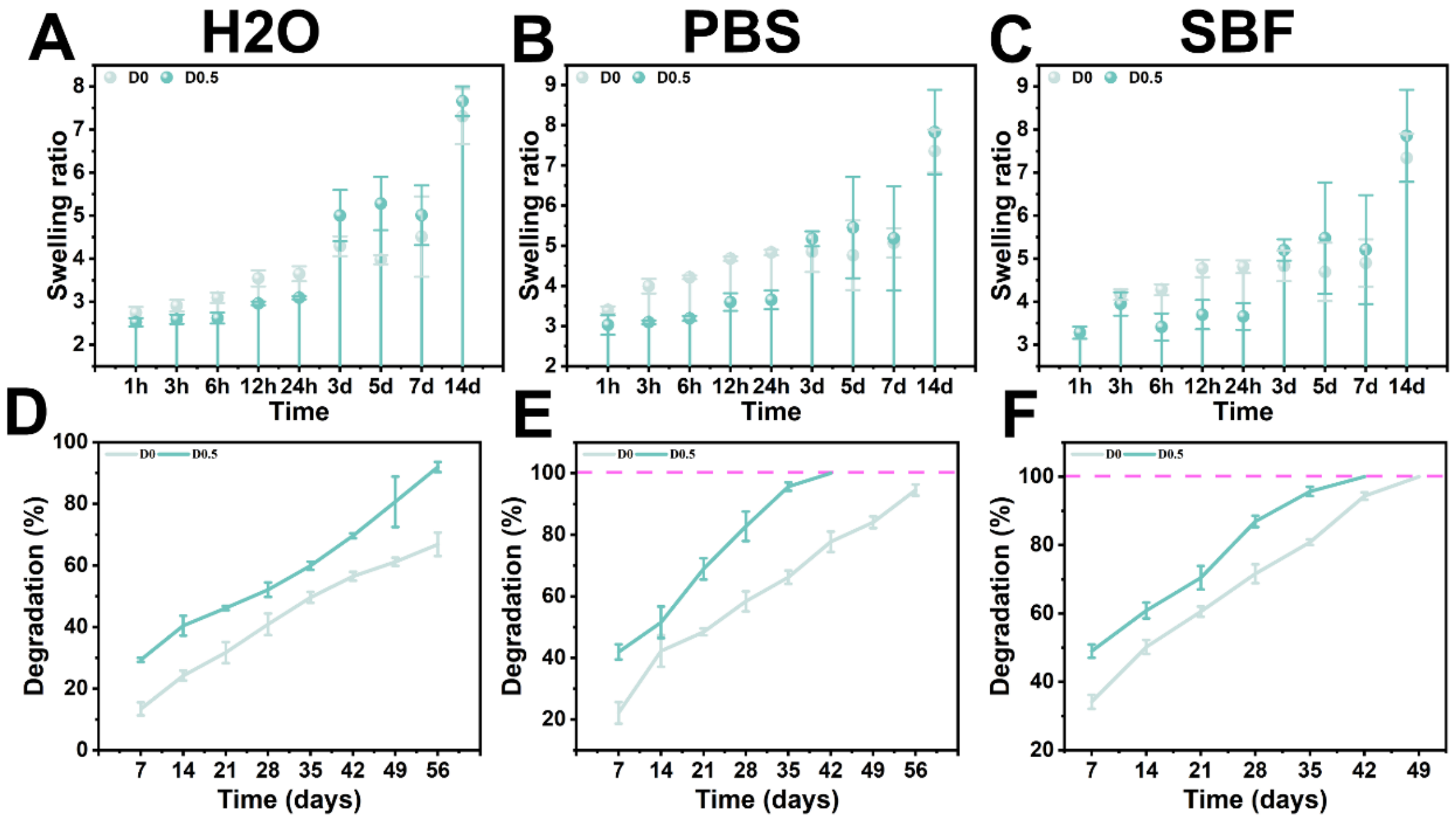

Figure S11. Swelling and degradation kinetics of the adhesive hydrogels. (A–C) Swelling behaviors of the adhesive hydrogels in diH_2O (A), PBS (B), and SBF (C) for 2 weeks. (D–F) Degradation profiles of the adhesive hydrogels in diH_2O (D), PBS (E), and SBF (F) for 8 weeks. N = 3.

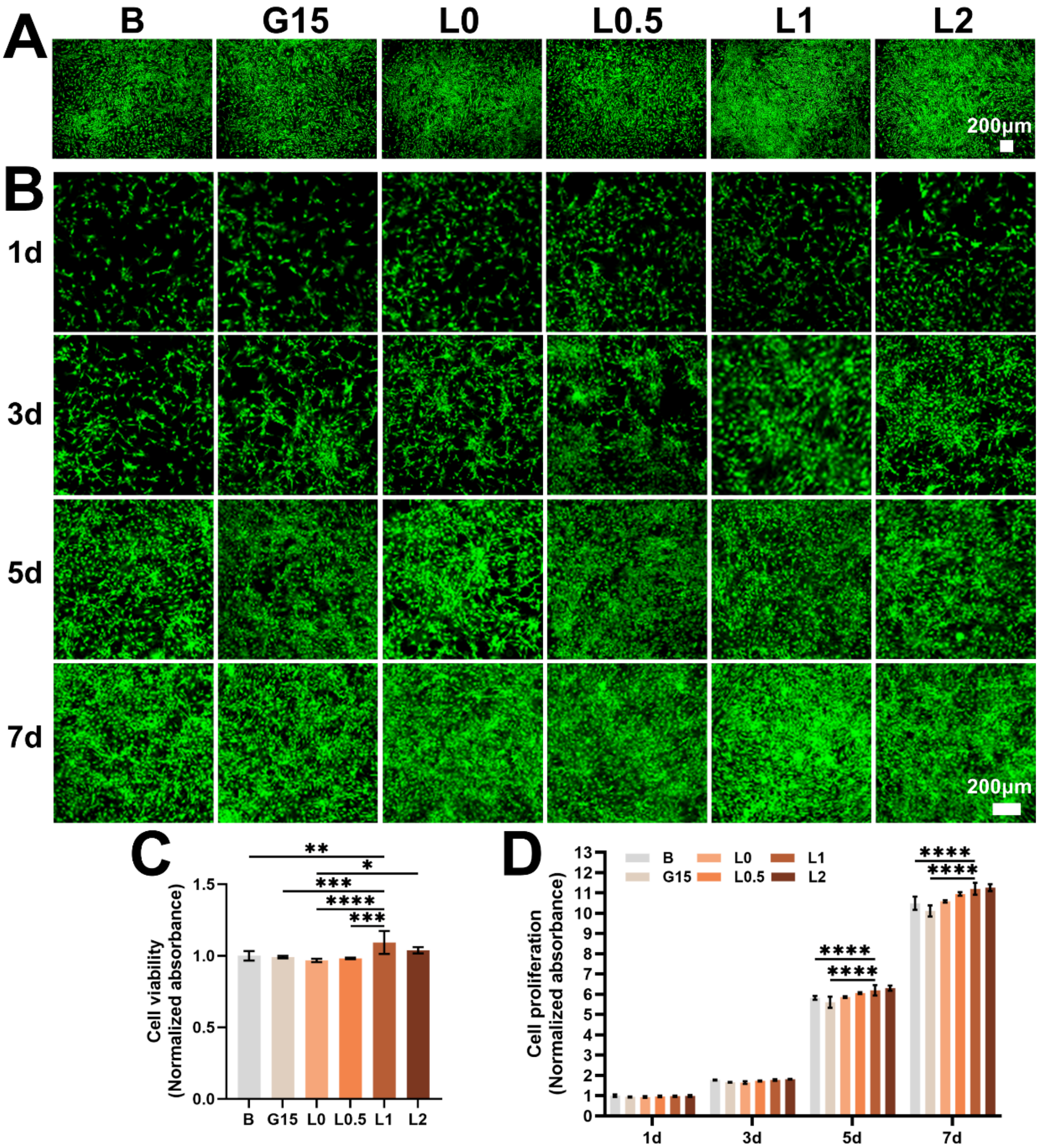

Figure S12. Cytocompatibility of the osteogenic layer hydrogels. (A) Representative fluorescence microscopy images of BMSCs stained with Calcein-AM/PI following 2 days of culture with the osteogenic layer hydrogels. (B) Representative fluorescence microscopy images of BMSCs stained with Calcein-AM/PI following 1, 3, 5, and 7 days of culture with the hydrogels. Quantitative analysis of BMSC viability (C; statistical analysis was performed using one-way ANOVA) and proliferation (D; statistical analysis was performed using two-way ANOVA) determined by the CCK-8 assay. Scale bar = 200 μm; *$P < 0.05$; **$P < 0.01$; ***$P < 0.001$; ****$P < 0.0001$. N = 3.

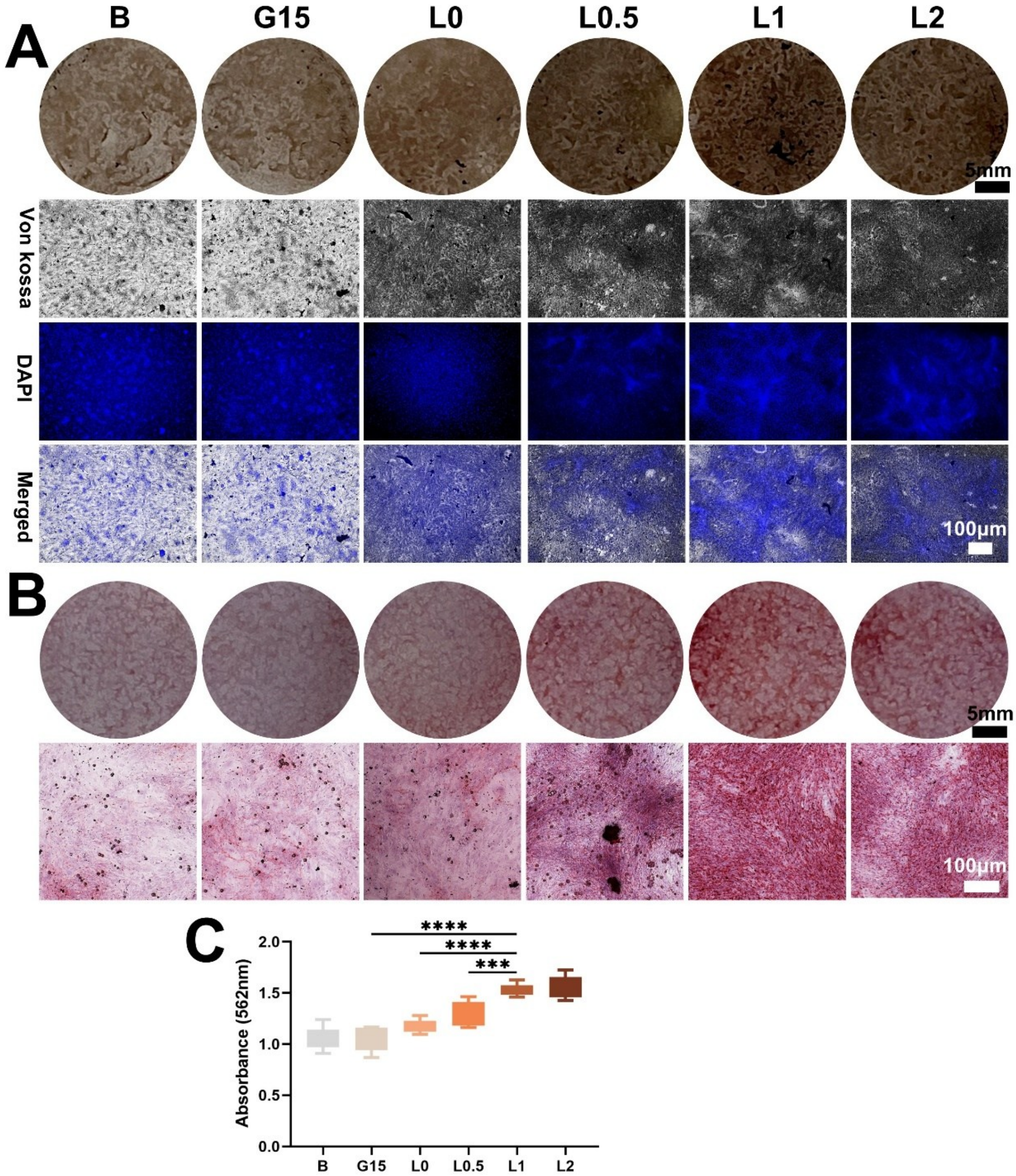

Figure S13. In vitro osteogenic activity of the osteogenic layer hydrogels. (A) Von Kossa staining of BMSCs following 14 days of culture with osteogenic layer hydrogels. (B) Alizarin Red S (ARS) staining of BMSCs following 14 days of culture with osteogenic layer hydrogels. (C) Quantitative analysis of the ARS staining. Statistical analysis in this figure was performed using one-way ANOVA. ***P < 0.001; ****P < 0.0001. N = 3.

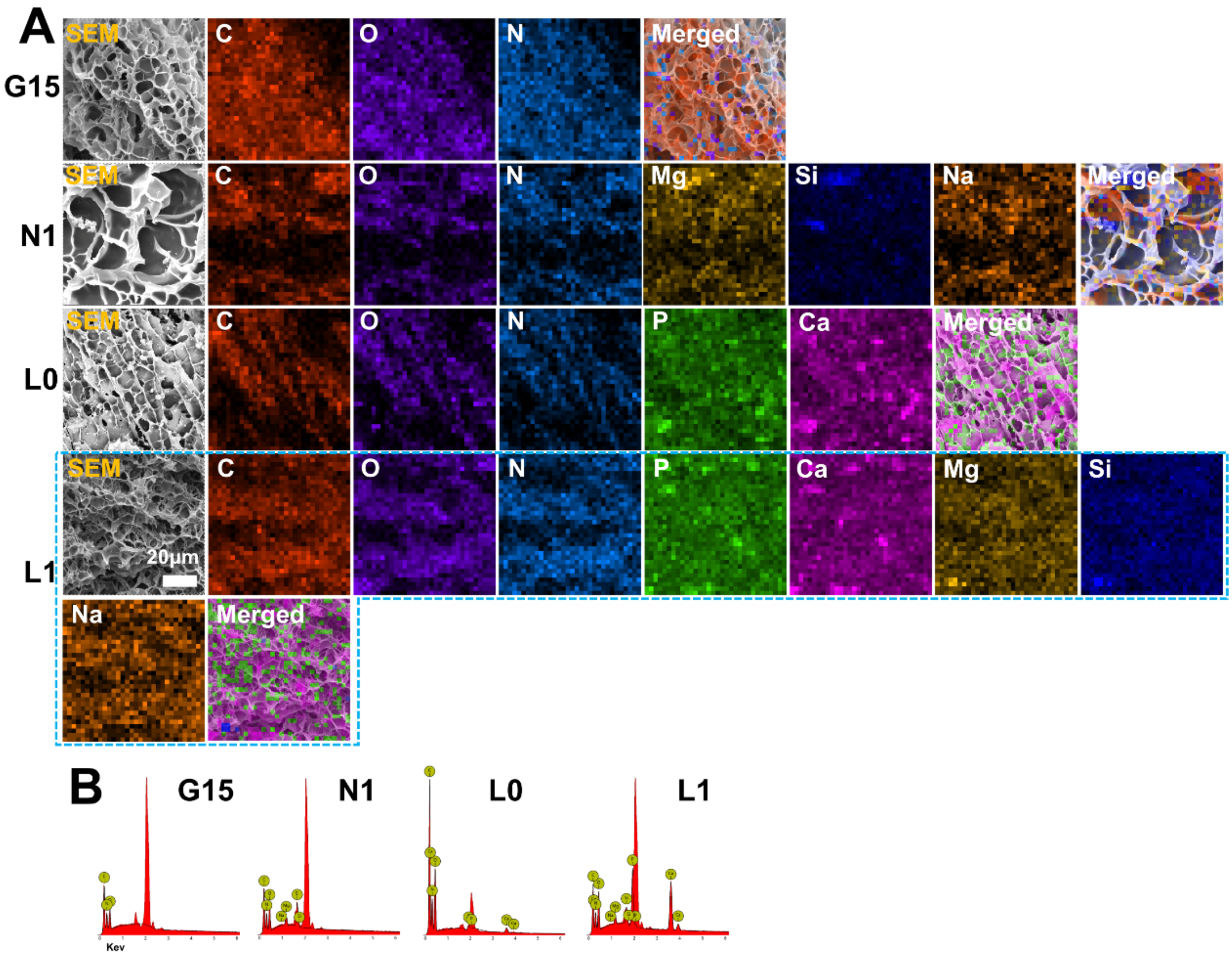

Figure S14. Morphology and elemental composition of the osteogenic layer hydrogels. (A) SEM micrographs and corresponding elemental maps of the osteogenic layer hydrogels. (B) Representative EDS spectra of the osteogenic layer hydrogels.

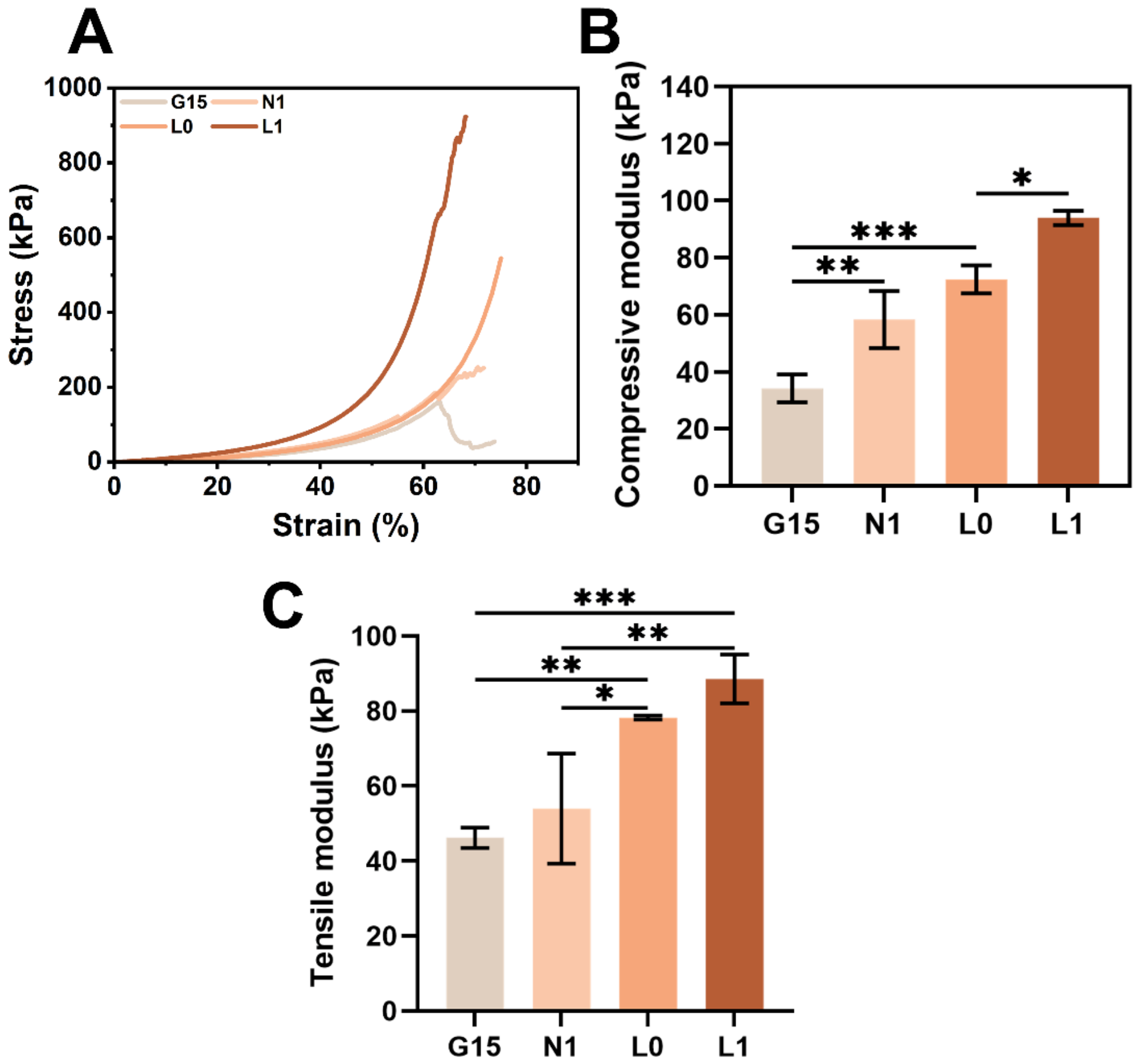

Figure S15. Mechanical properties of the osteogenic layer hydrogels. (A) Representative stress-strain curves of various osteogenic layer hydrogels. (B) Compressive modulus. (C) Tensile modulus. Statistical analysis in this figure was performed using one-way ANOVA. *P < 0.05; **P < 0.01; ***P < 0.001. N = 3.

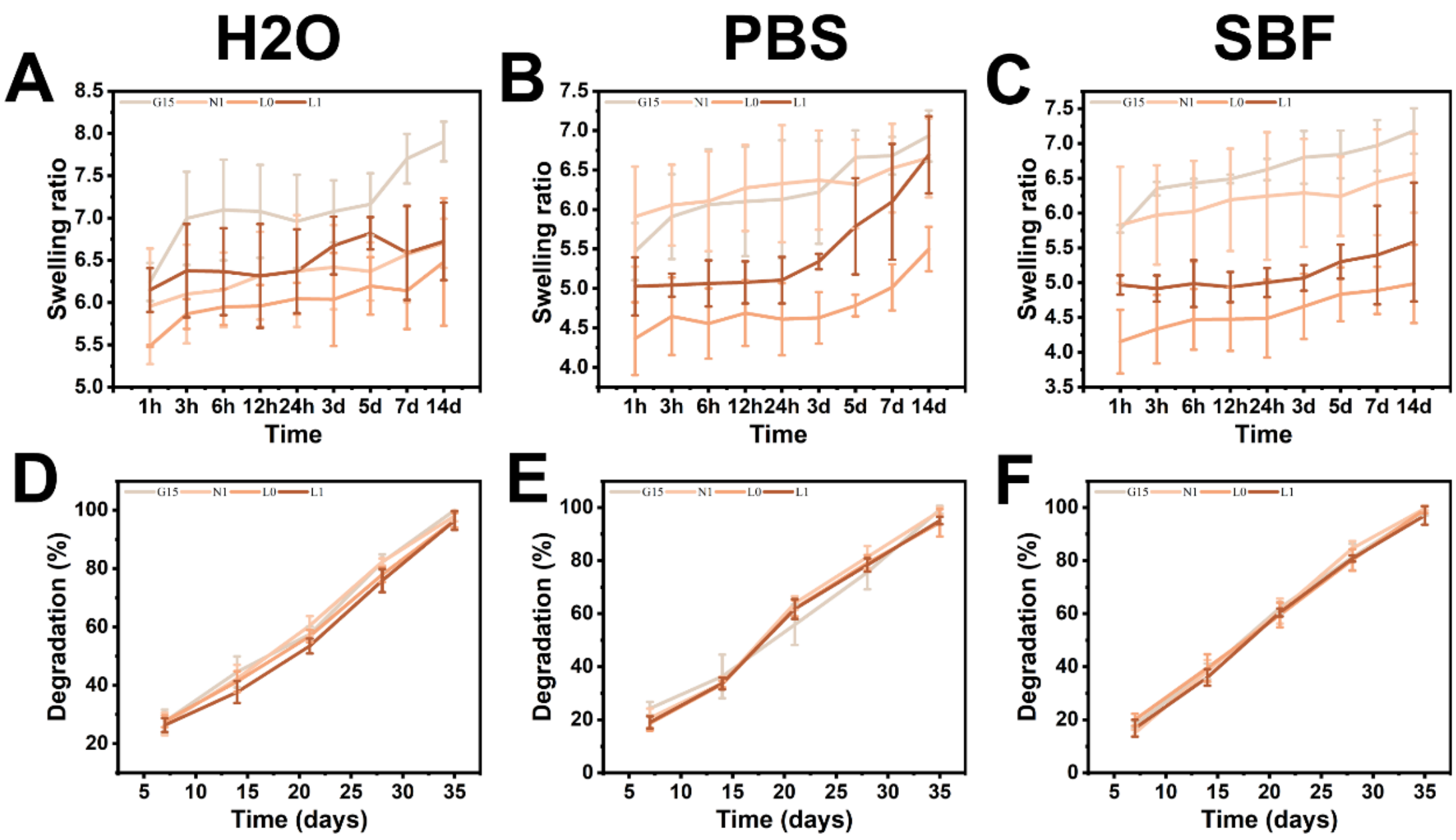

Figure S16. Swelling and degradation kinetics of the osteogenic layer hydrogels. (A–C) Swelling behaviors of the osteogenic layer hydrogels in diH_2O (A), PBS (B), and SBF (C) for 2 weeks. (D–F) Degradation profiles of the osteogenic layer hydrogels in diH_2O (D), PBS (E), and SBF (F) for 5 weeks. N = 3.

A

Unite cell	Split P
Orientation	UVW
Volume	Square
Unite cell size	3×3×3mm
Approx. thickness	0.5mm
Mid-surface offset	0.35mm

B

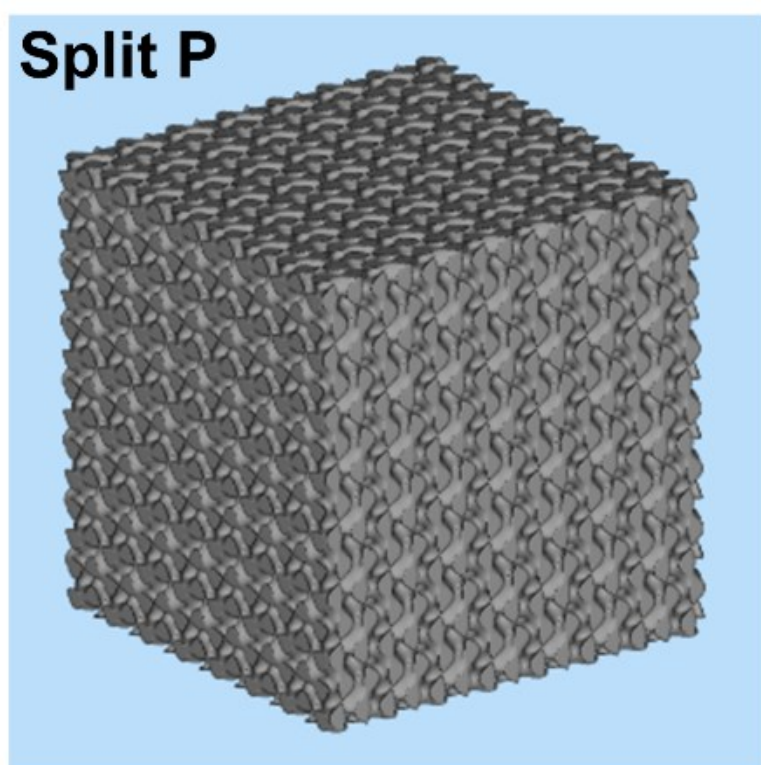

Figure S17. Parent structure construction of the osteogenic layer of the scaffold. (A) Construction parameters of the triply periodic minimal surface (TPMS) Split P structure. (B) Parent model of the TPMS Split P structure.

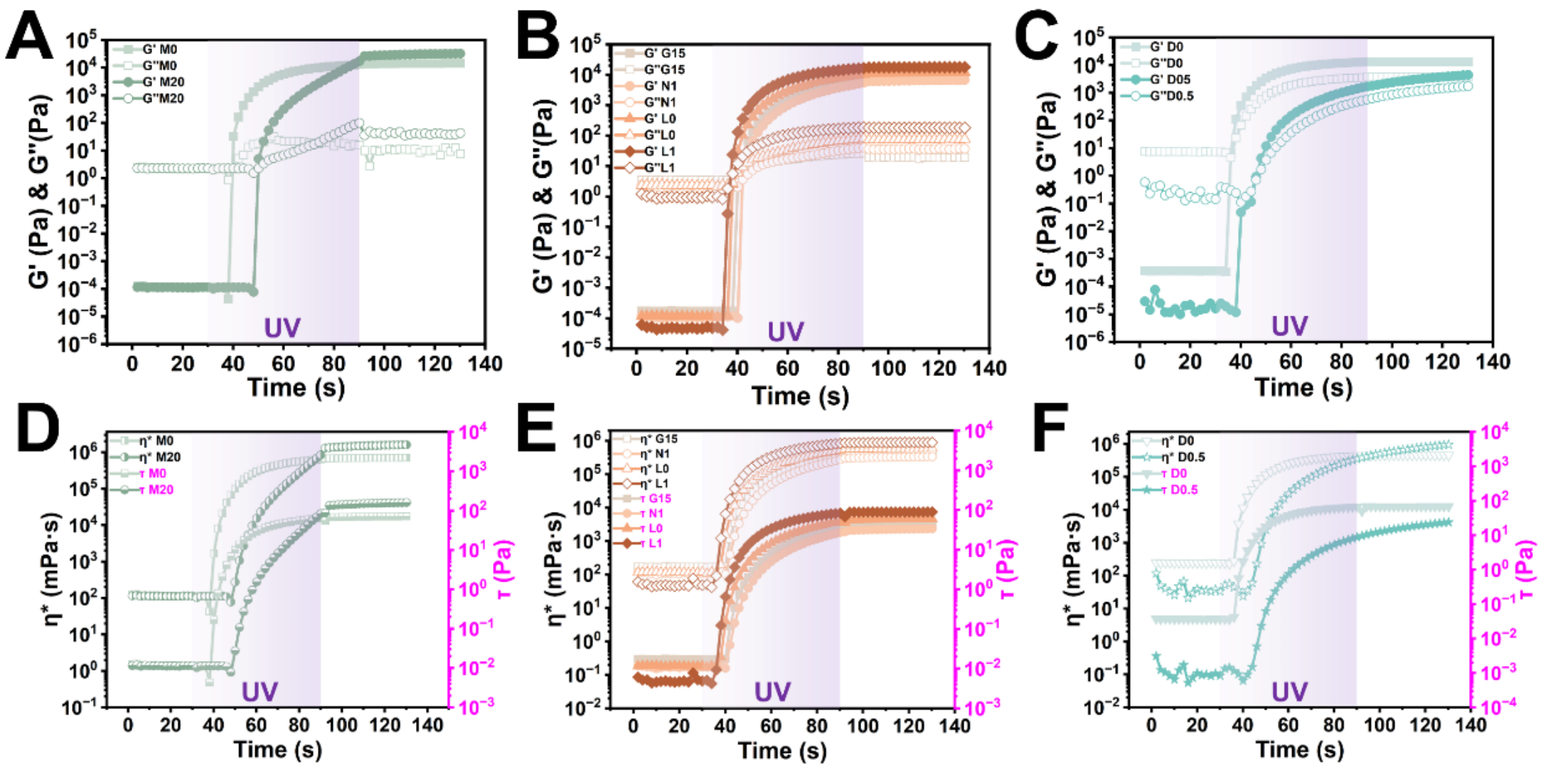

Figure S18. Photosensitivity testing of inks for each layer. (A-C) Changes in viscoelastic properties of the barrier layer (A), the osteogenic layer (B), and the adhesive layer (C) inks under UV exposure. (D-F) Changes in complex viscosity and loss modulus of the barrier layer (D), the osteogenic layer (E), and the adhesive layer (F) inks under UV irradiation.

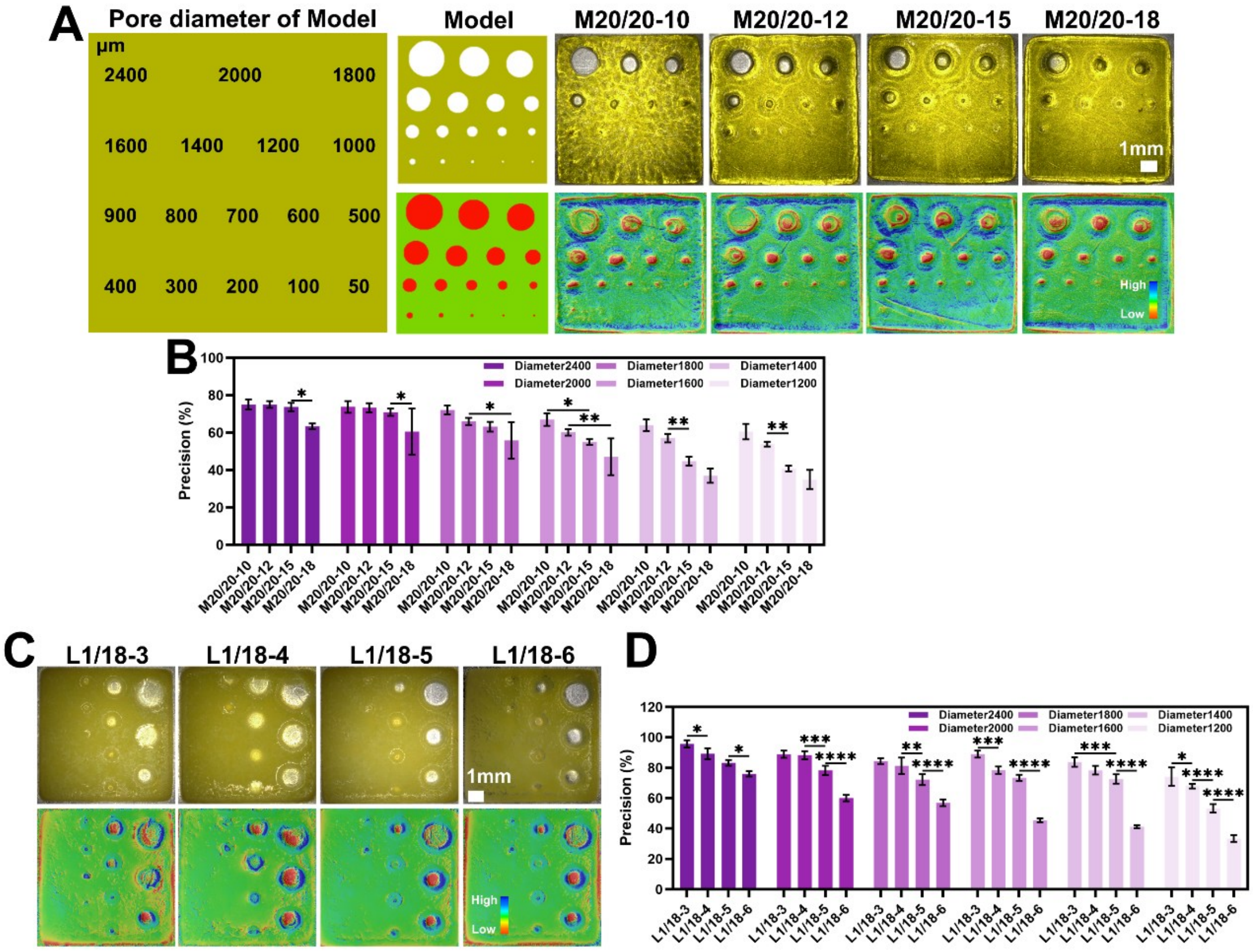

Figure S19. Exploration of printing parameters for barrier layer and osteogenic layer inks. (A) Demonstration of precision test specimens printed with barrier layer ink (M20) under different printing parameters. (B) Quantitative comparison of pore size precision on precision test specimens printed with barrier layer ink (M20) under different printing parameters. (C) Photographs of precision test specimens printed with osteogenic layer ink (L1) under varying parameters. (D) Quantitative comparison of pore size precision on precision test specimens printed with osteogenic layer ink (L1) under different printing parameters. Statistical analysis in this figure was performed using two-way ANOVA. *P < 0.05; **P < 0.01; ***P < 0.001; ****P < 0.0001. N = 3.

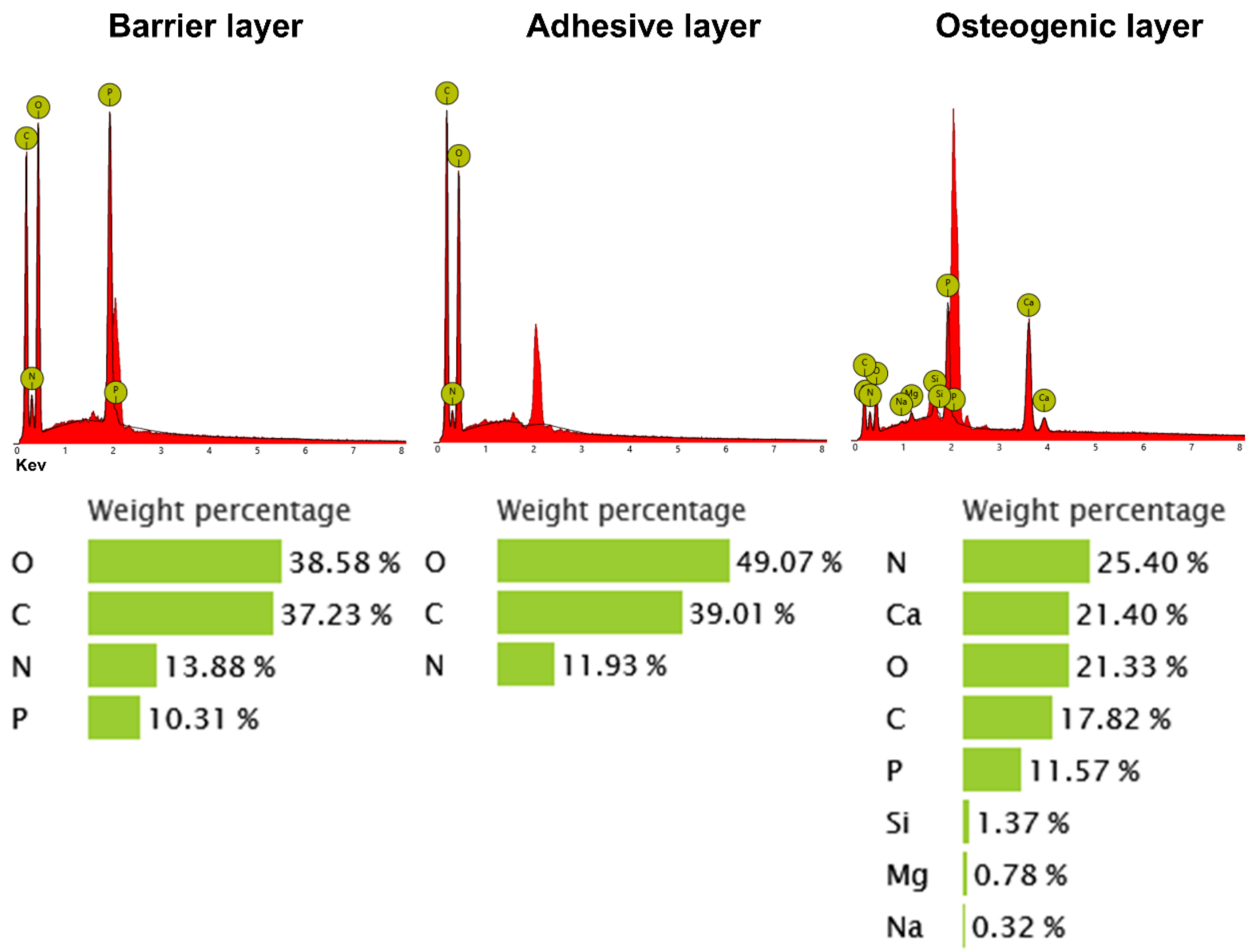

Figure S20. Energy-dispersive X-ray spectroscopy (EDS) analysis of the three functional layers (barrier layer, adhesive layer, and osteogenic layer) of the scaffold. Corresponding weight percentages of detected elements are shown below each spectrum.

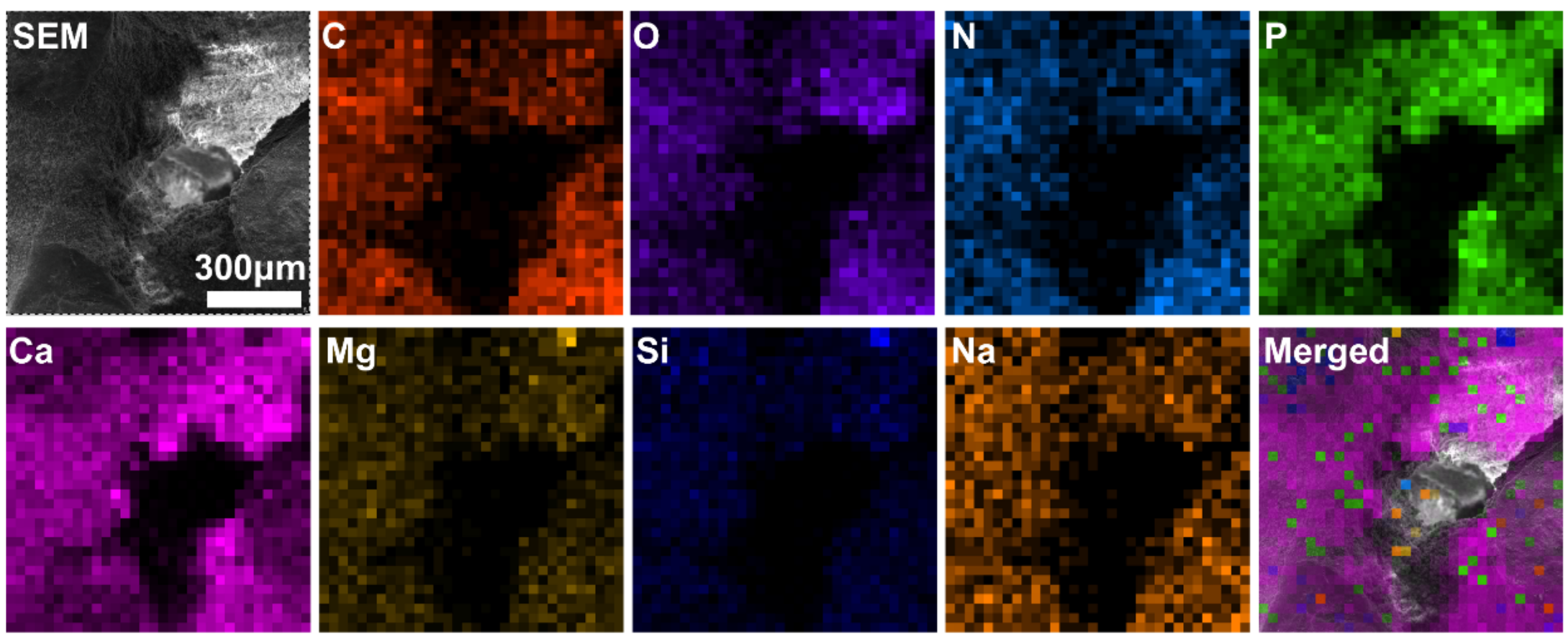

Figure S21. SEM and elemental mapping images of the osteogenic layer of the scaffold.

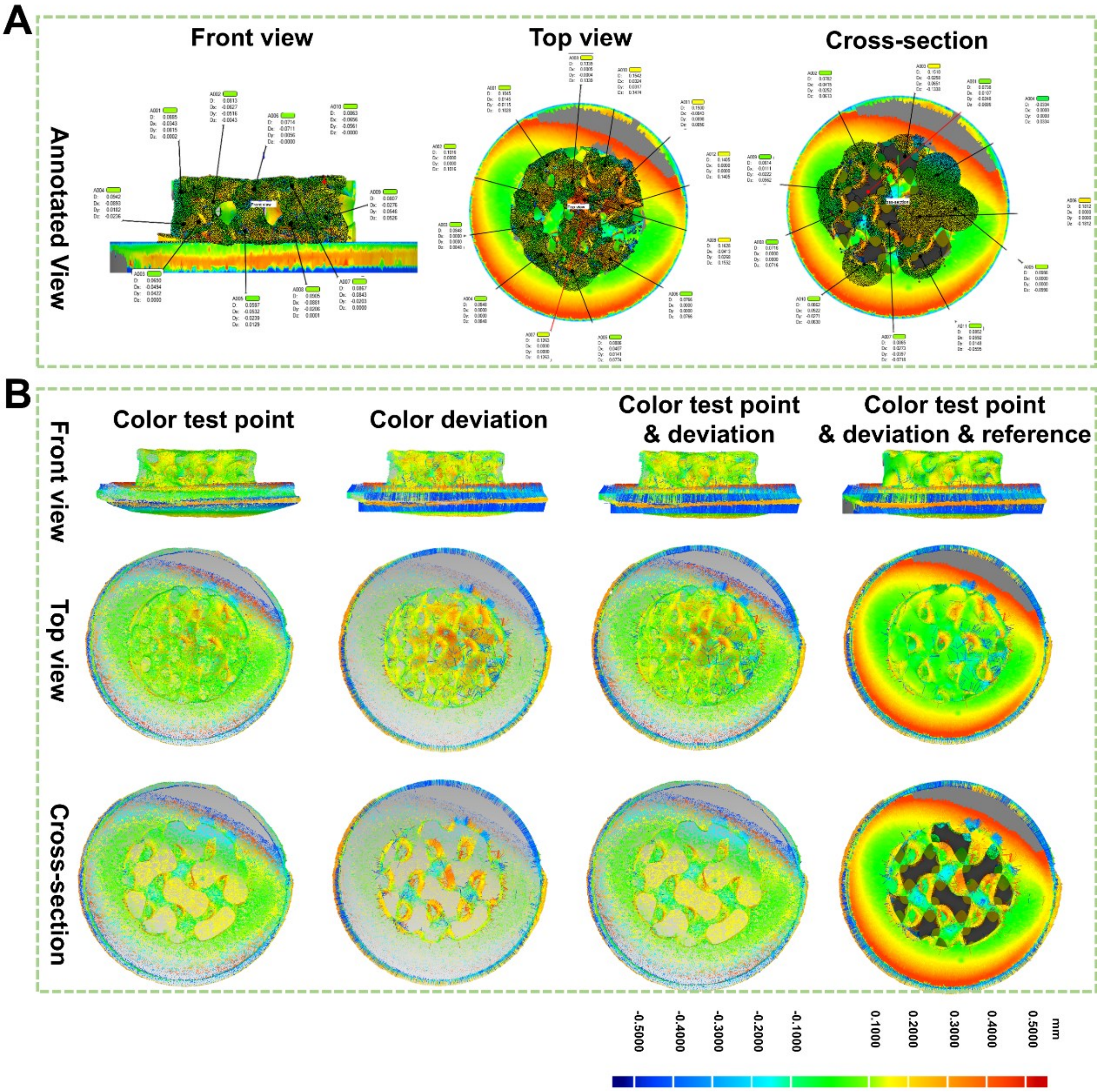

Figure S22. 3D deviation analysis of the osteogenic layer of the scaffold performed using Geomagic Qualify. (A) Cloud maps of dimensional deviations at random locations in different sections of the osteogenic layer. (B) Dimension deviation cloud map of the scaffold osteogenic layer across different planes in multi-view mode.

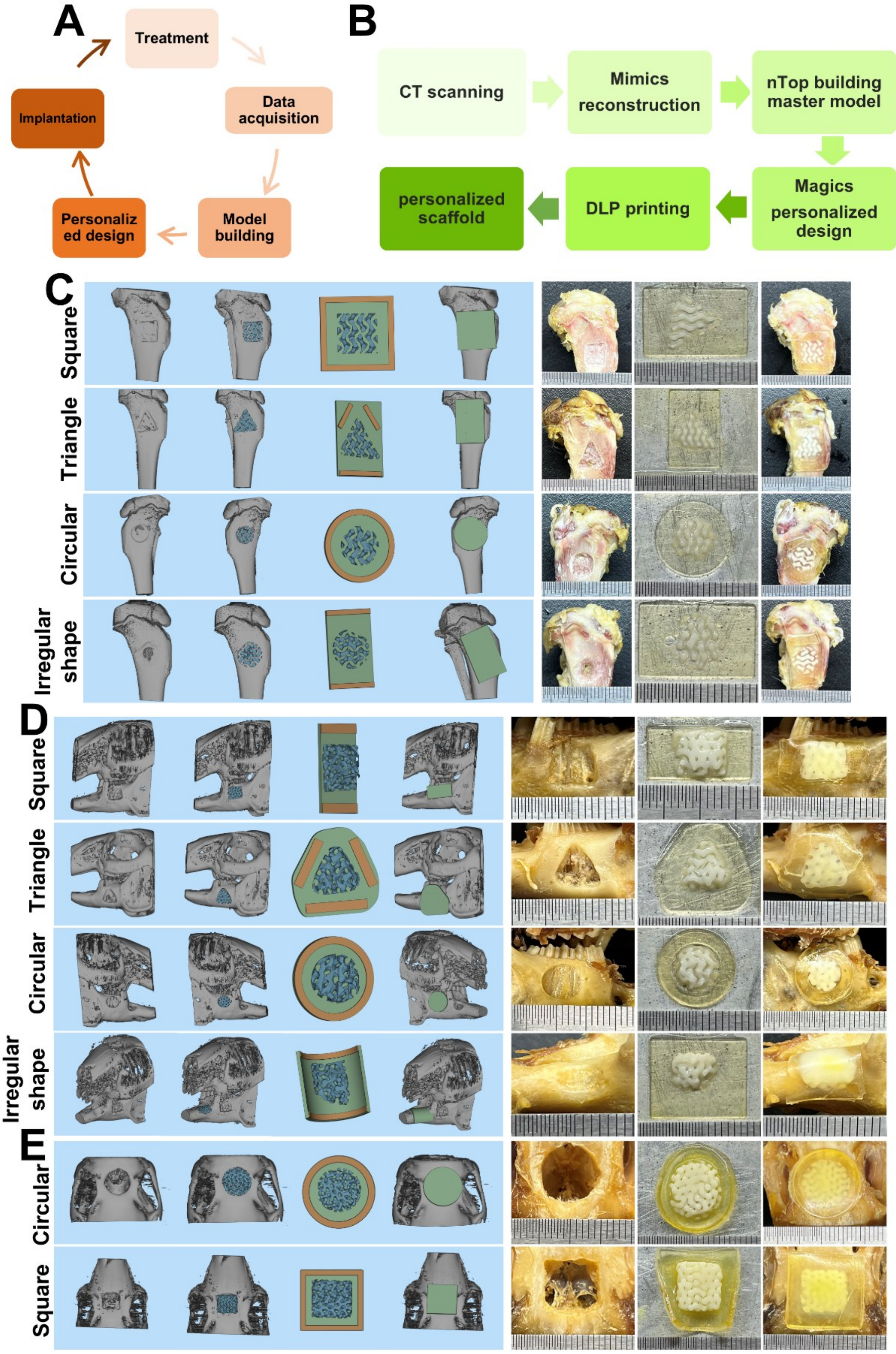

Figure S23. Personalized design and fabrication of the scaffold based on the morphology of bone defects. (A) Overall workflow of personalized treatment. (B) Process from personalized scaffold design to manufacturing for bone defects. (C-E) Different shapes of defects in rabbit tibia (C), mandible (D), and skull (E), as well as the corresponding personalized design and fabrication of scaffolds.

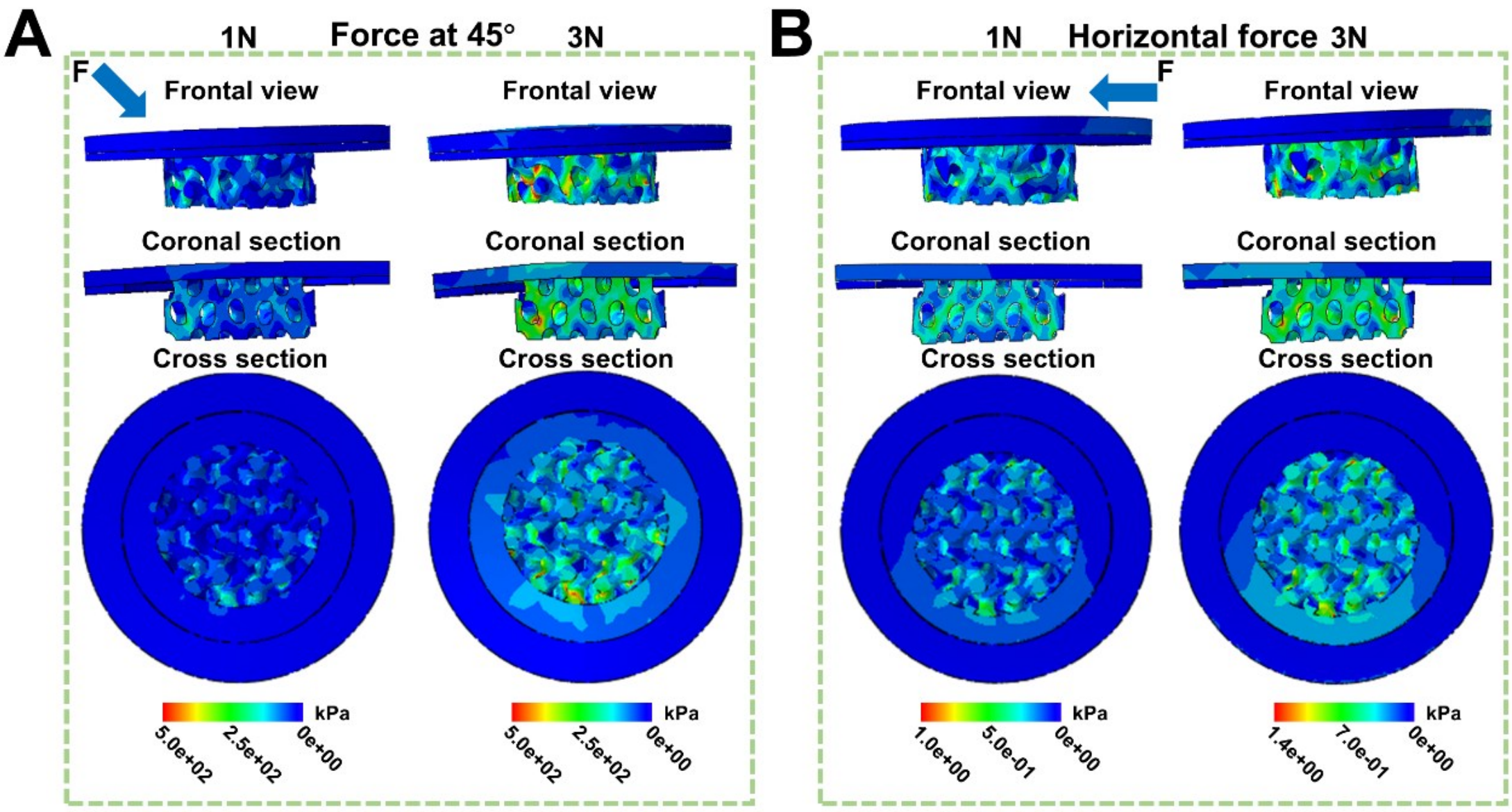

Figure S24. Finite element analysis (FEA) of the scaffold. (A) Stress distribution of the scaffold under different pressures (1N and 3N) at a 45° angle. (B) Stress distribution of the scaffold under different horizontal force (1N and 3N).

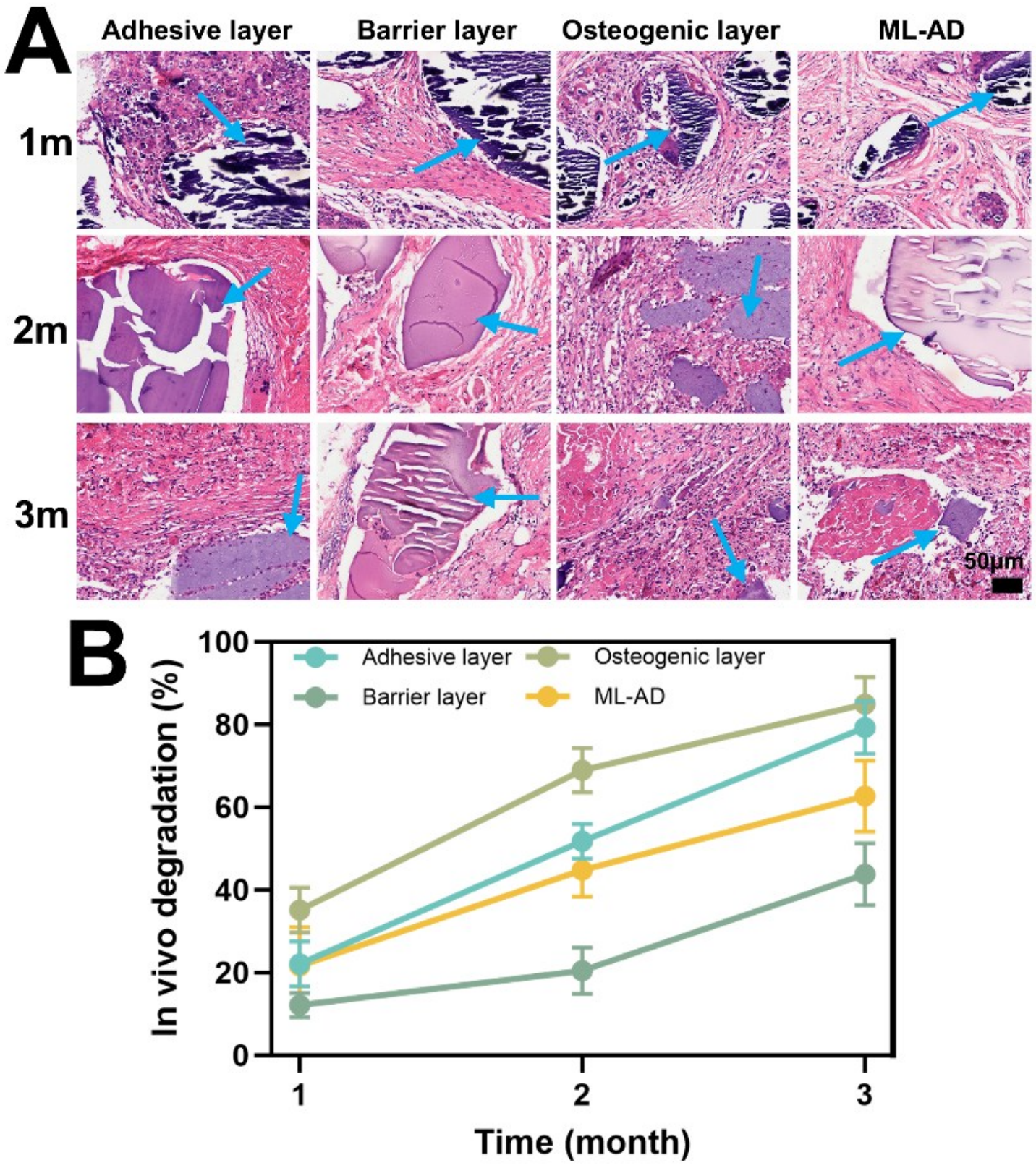

Figure S25. Subcutaneous degradation of the scaffold and each individual layer in rabbits. (A) HE staining at 1, 2, and 3 months post-implantation of scaffolds and individual layers. (B) In vivo degradation kinetics of the scaffold and each individual layer in rabbits at different time points after subcutaneous implantation. N = 4.

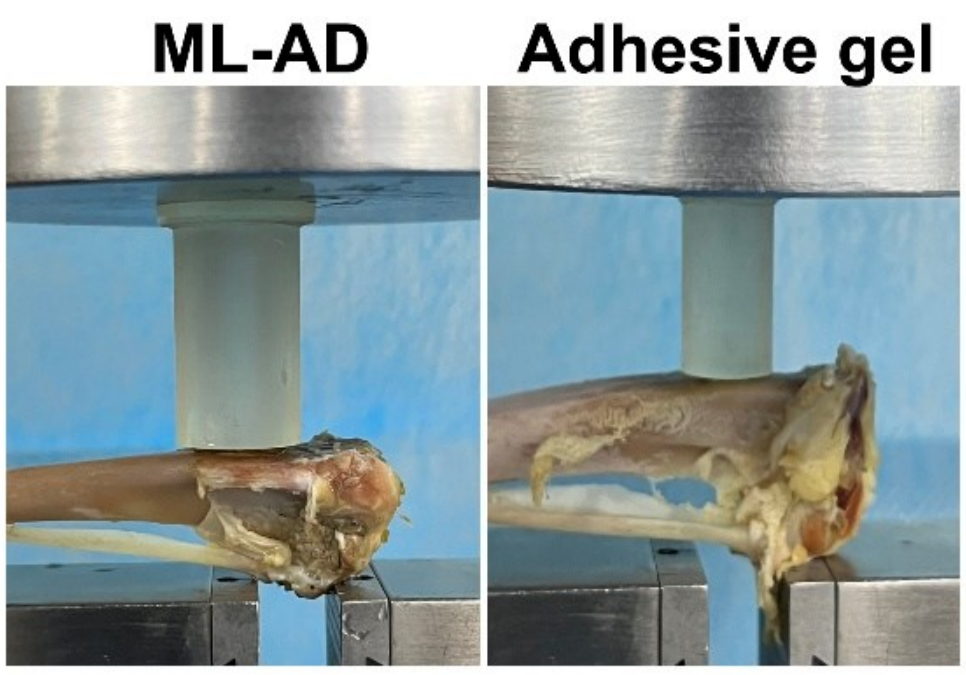

Figure S26. Pressure force magnitude and pressure duration applied to the scaffold and adhesive bonding during bone bonding, regulated by a universal testing machine.

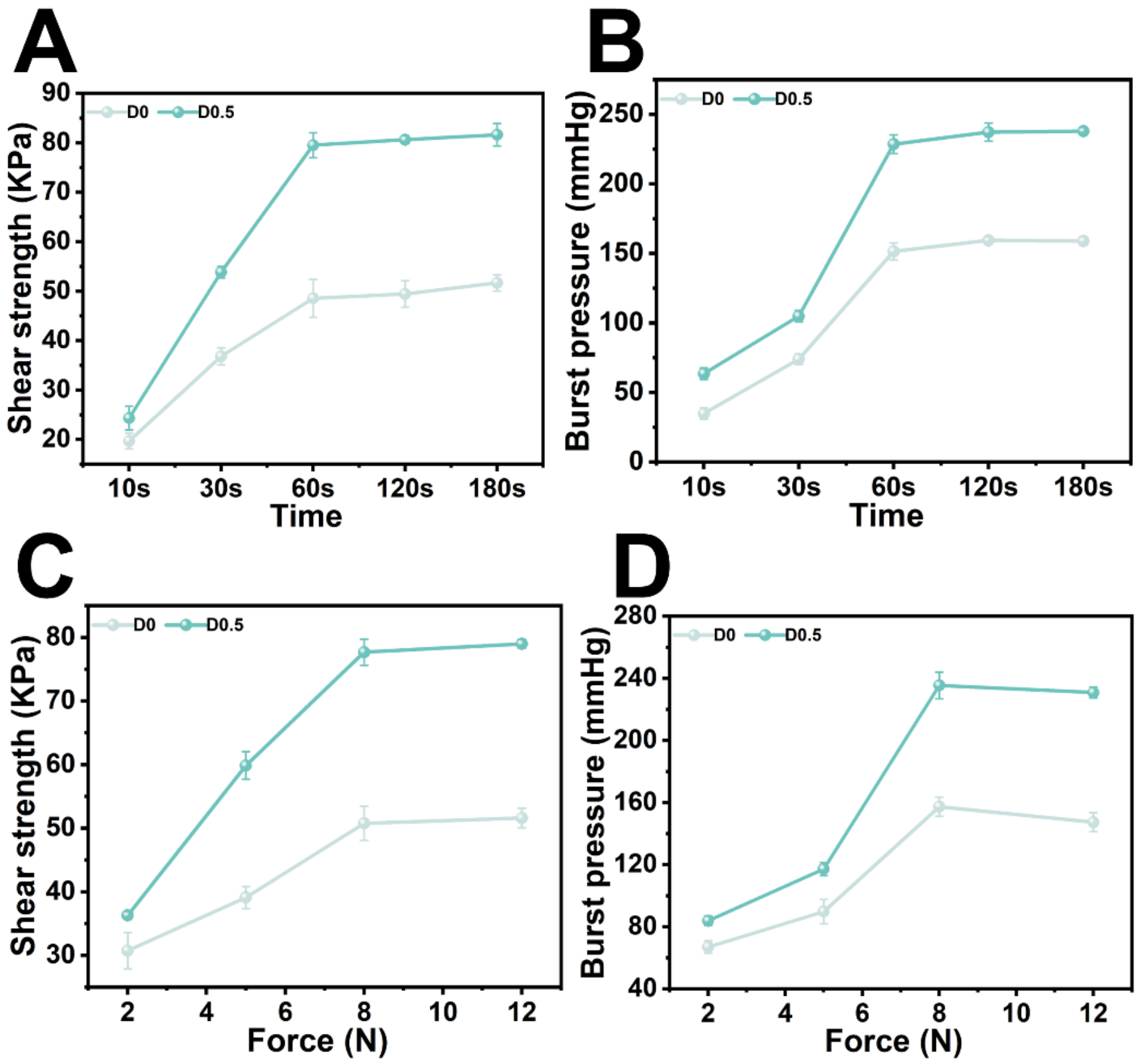

Figure S27. Bone bonding performance of the adhesive layer gels under different pressure forces and compression times. (A) Shear strength of bone bonding of the adhesive under a constant compressive force (8 N) and varying pressure times. (B) Burst pressure of bone bonding of the adhesive gels under a constant compressive force (8 N) and different compression times. (C) Shear strength of bone bonding of the adhesive gels at the same pressing time (60 s) under different pressing forces. (D) Burst pressure of bone bonding of the adhesives under a constant compression time (60 s) and different compressive forces. N=3.

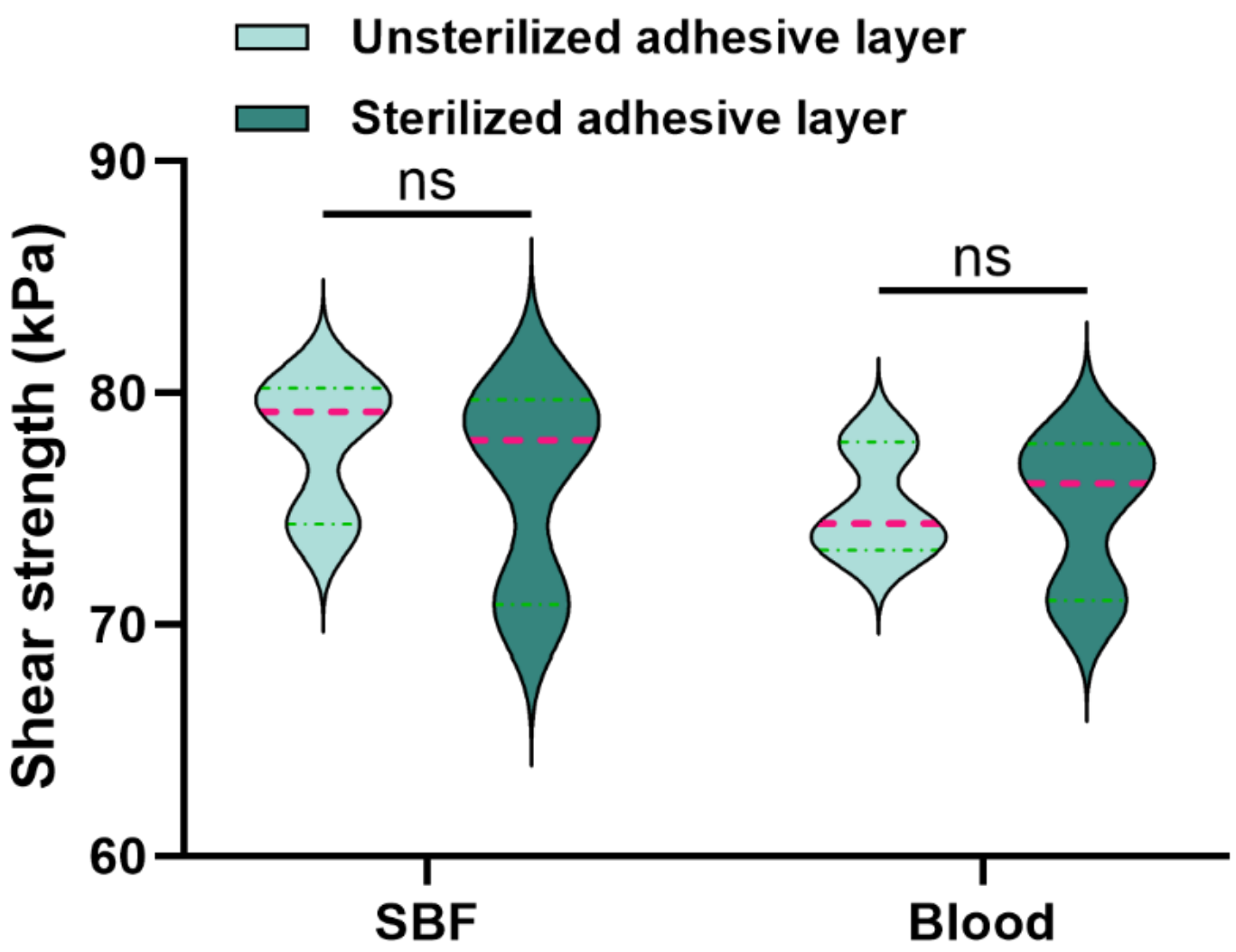

Figure S28. Comparison of bone adhesion performance of the adhesive layer before and after sterilization in the presence of simulated body fluid (SBF) and blood. ns indicates no significant difference (P > 0.05). Statistical analysis in this figure was performed using one-way ANOVA. “ns” indicates no statistical significance (P>0.05). N=3.

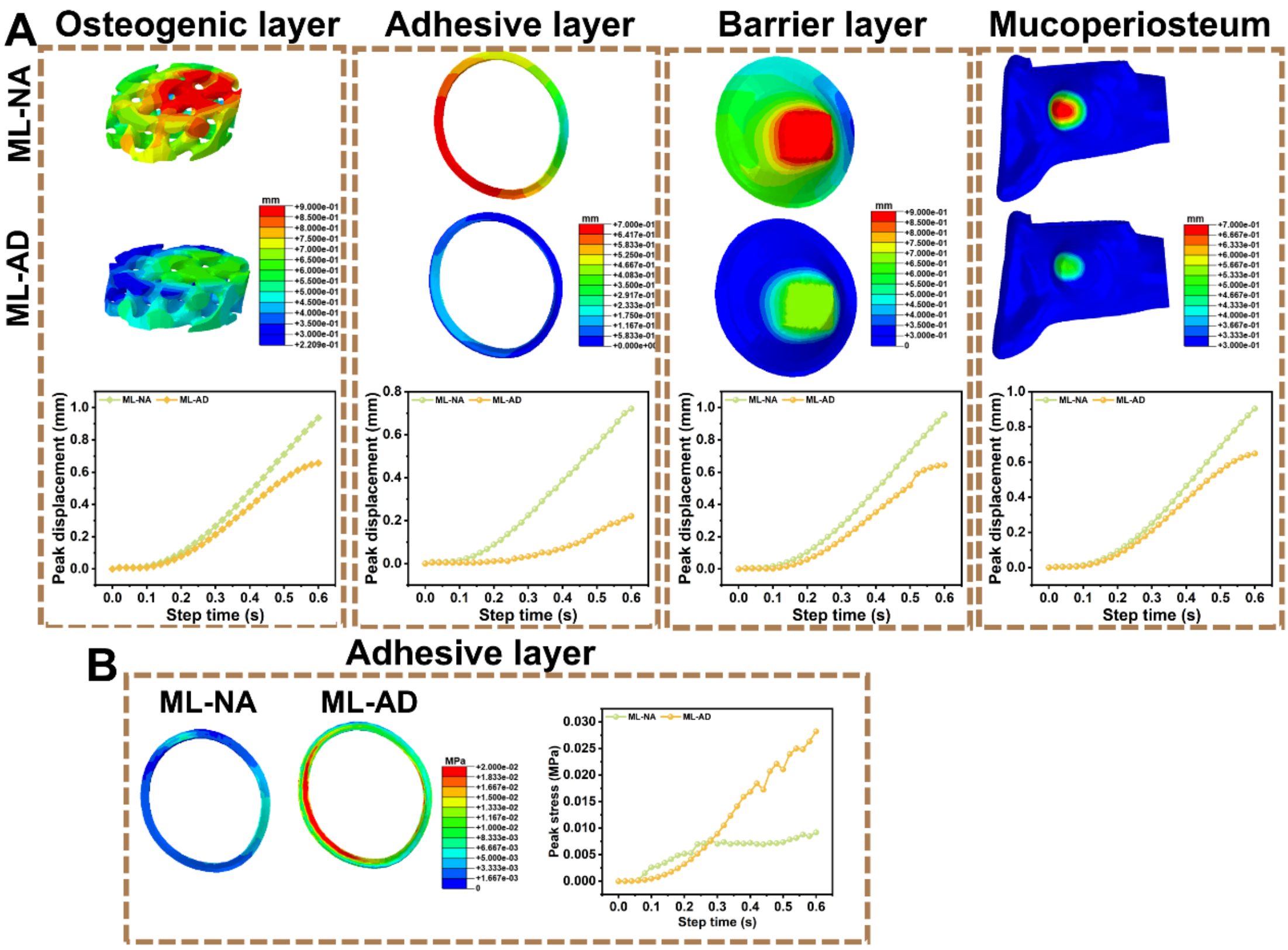

Figure S29. FEA of the scaffold bonded to mandibular defects under in vivo simulation. (A) Displacement distribution cloud maps and step time-peak displacement curves of the osteogenic layer, adhesive layer, barrier layer of the scaffold, and the periosteum at 0.6 s of dynamic force loading. (B) Stress distribution cloud map and step time-peak force curve of the adhesive layer of the scaffold at 0.6 s of dynamic force loading.

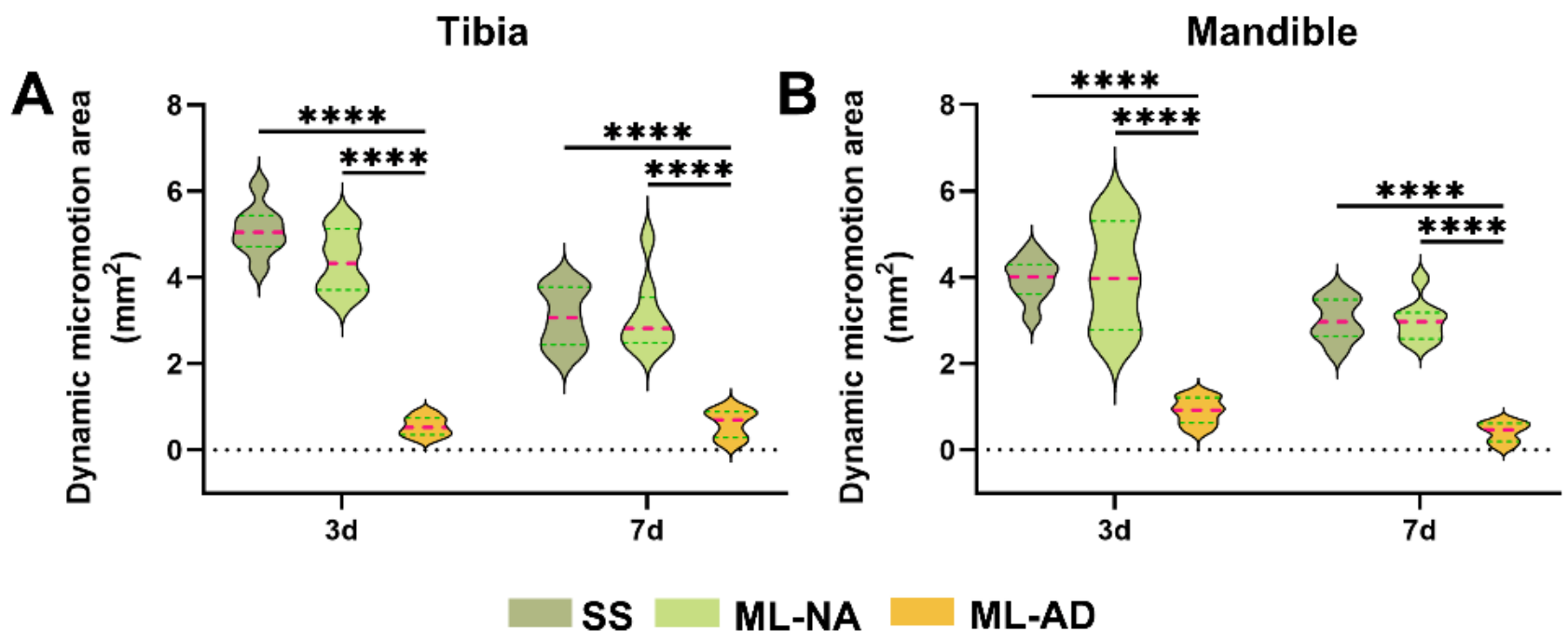

Figure S30. Ultrasound-based assessment of scaffold micromotion at 3 and 7 days following implantation of SS, ML-NA and ML-AD scaffolds in rabbit tibia (A) and mandible (B). ****P < 0.0001. N = 3. Statistical analyses in this figure were performed via two-way ANOVA.

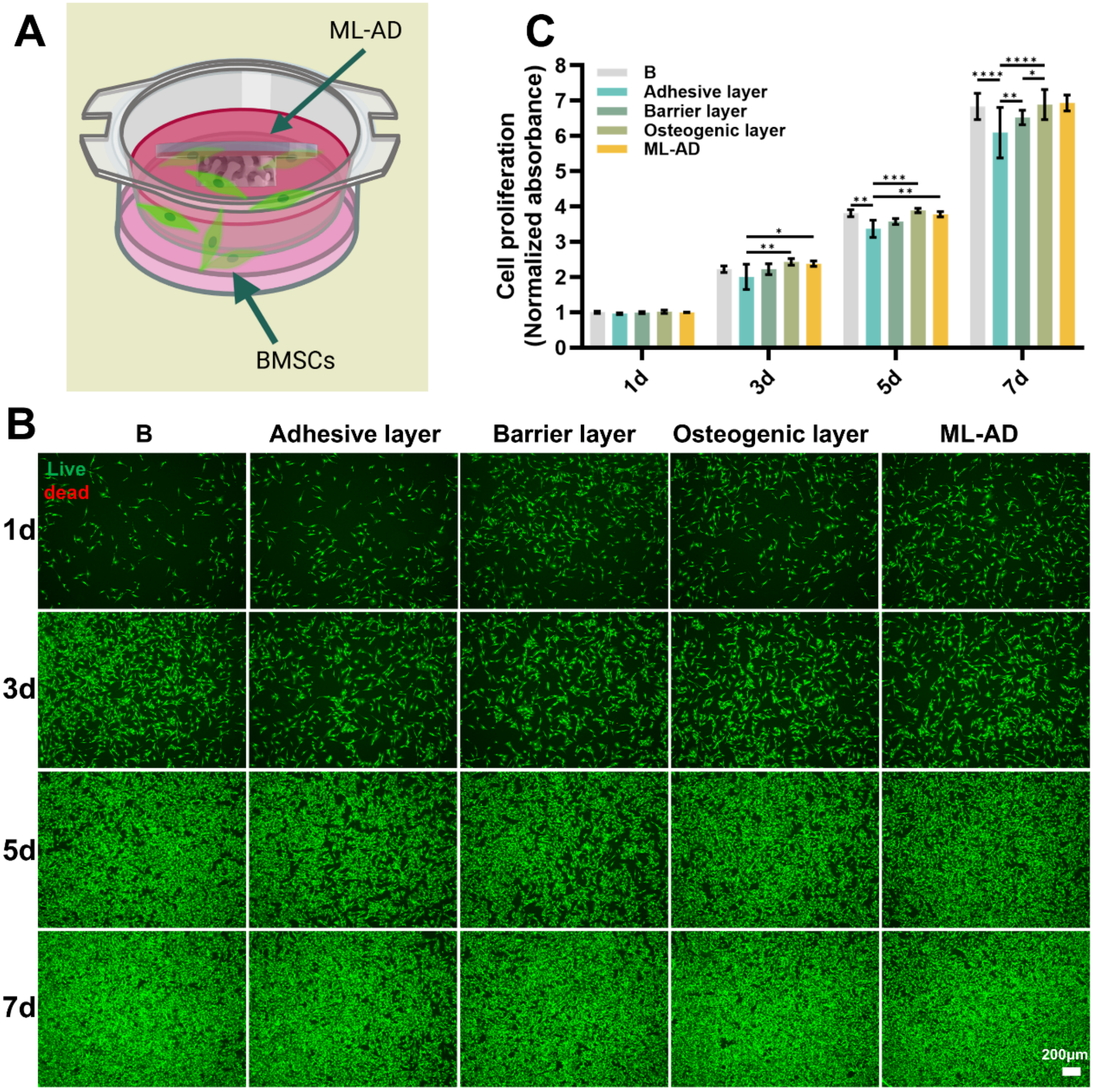

Figure S31. Cytocompatibility of the scaffold. (A) Schematic diagram of BMSCs cultured with the scaffold. (B) Representative Calcein-AM/PI staining images of BMSCs following 1, 3, 5, and 7 days of culture with the scaffold. (C) Quantitative analysis via CCK-8 assay for BMSCs cultured with the scaffold at 1, 3, 5, and 7 days. Statistical analyses in this figure were performed via two-way ANOVA. *P < 0.05; **P < 0.01; ***P < 0.001; ****P < 0.0001. N = 3.

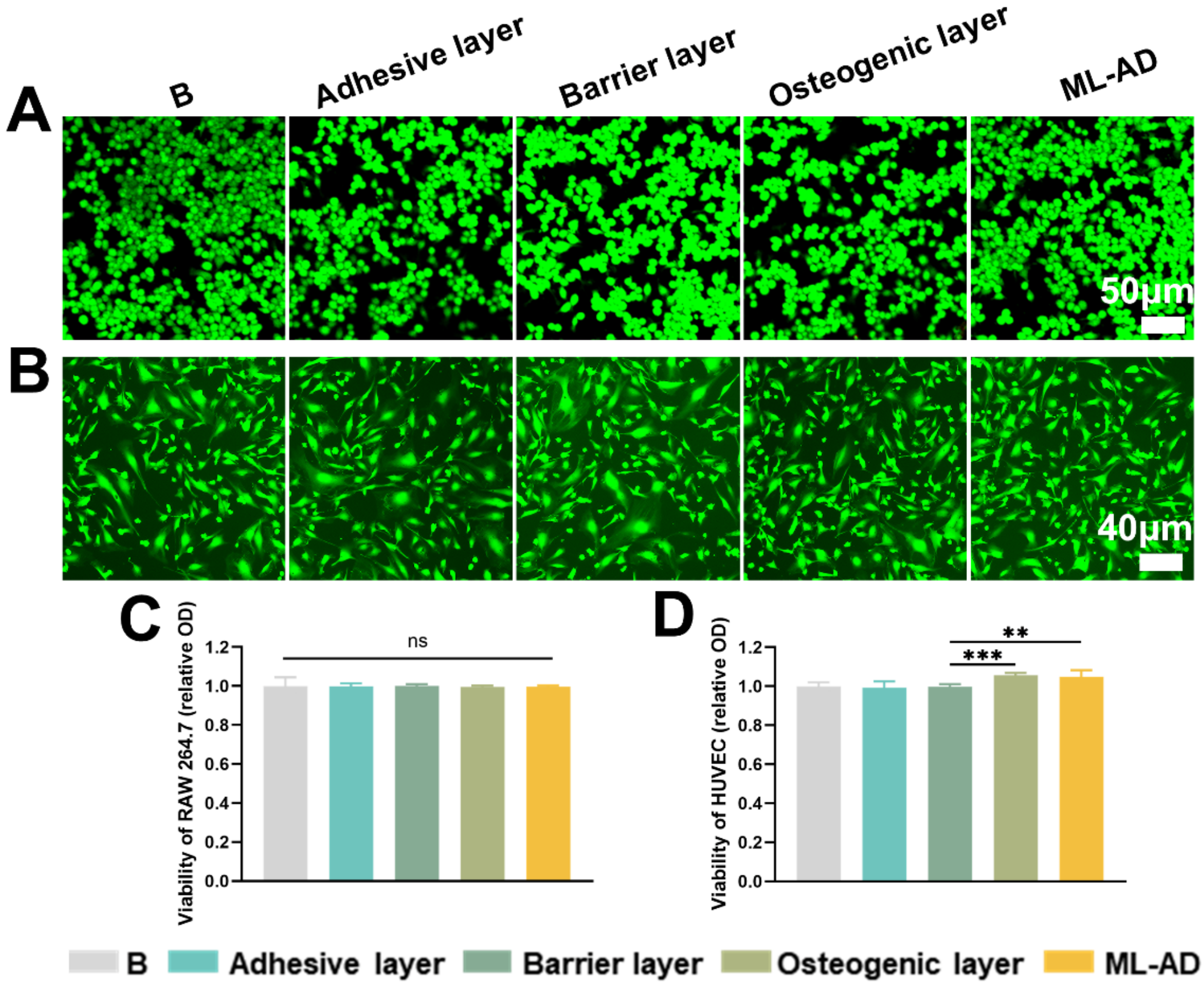

Figure S32. Cytotoxicity evaluation of the scaffold. (A, B) Representative Calcein-AM/PI staining images of RAW264.7 macrophages (A) and HUVECs (B) following 2 days of culture with the scaffold. (C, D) Quantitative analysis via CCK8 assay after 2 days of RAW264.7 macrophages (C) and HUVECs (D) cultured with the scaffold. "ns" denotes not statistically significant ($P > 0.05$); Statistical analyses in this figure were performed via one-way ANOVA. $**P < 0.01$; $***P < 0.001$. N = 3.

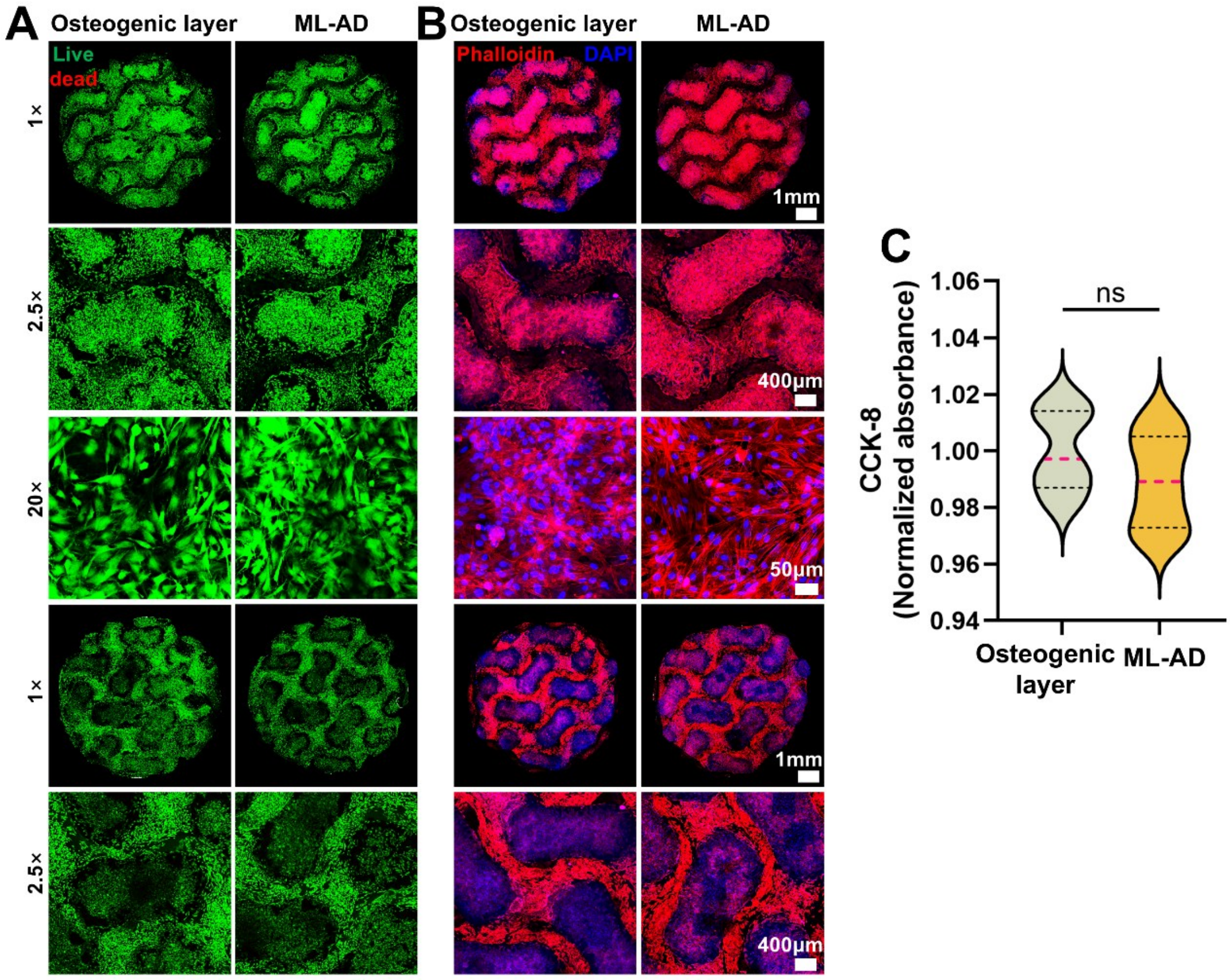

Figure S33. Growth of BMSCs on the scaffold and its osteogenic layer. (A) Representative Calcein-AM/PI staining images of BMSCs following 2 days of culture on the scaffold and its osteogenic layer. (B) Representative phalloidin staining images of BMSCs following 2 days of culture on the scaffold and its osteogenic layer. (C) Quantitative analysis via CCK8 assay after 2 days of BMSC culture on the scaffold and its osteogenic layer. "ns" denotes not statistically significant ($P > 0.05$); Statistical analyses in this figure were performed via unpaired Student's t-test. N = 3.

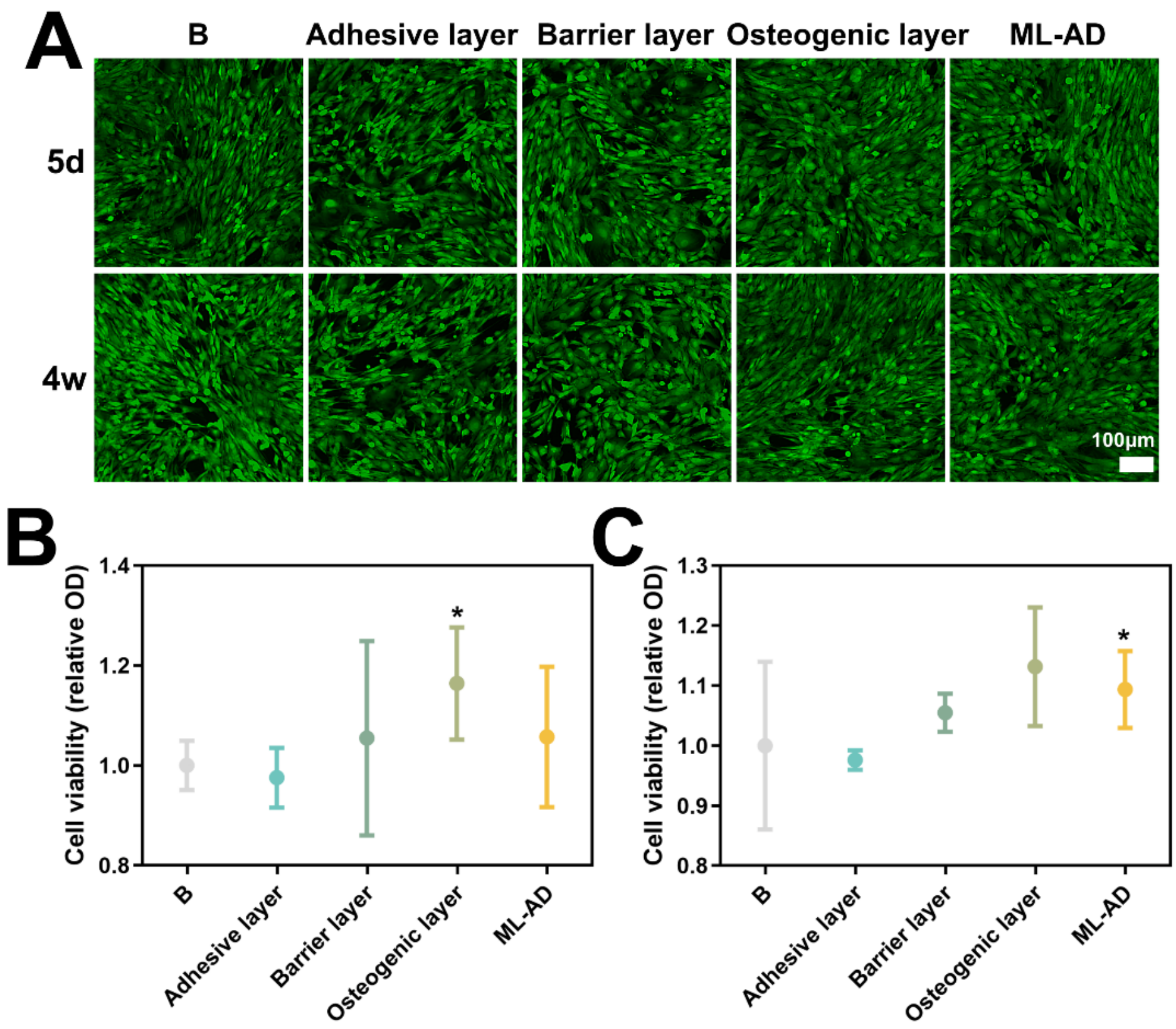

Figure S34. Cytocompatibility of degradation products from the scaffold and each individual layer. (A) Representative Calcein-AM/PI staining images of BMSCs following 2 days of culture with degradation extracts of the scaffold and each individual layer (degraded for 5 days and 4 weeks). (B) Quantitative analysis via CCK8 assay after 2 days of BMSC culture with scaffolds and single-layer degradation extracts (degradation for 5 days). (C) Quantitative analysis of BMSCs following 2 days of culture with 4-week degradation extracts of the scaffold and each individual layer, determined by the CCK-8 assay. Statistical analyses in this figure were performed via one-way ANOVA. * Denotes $P < 0.05$ compared with the adhesive layer. N = 3.

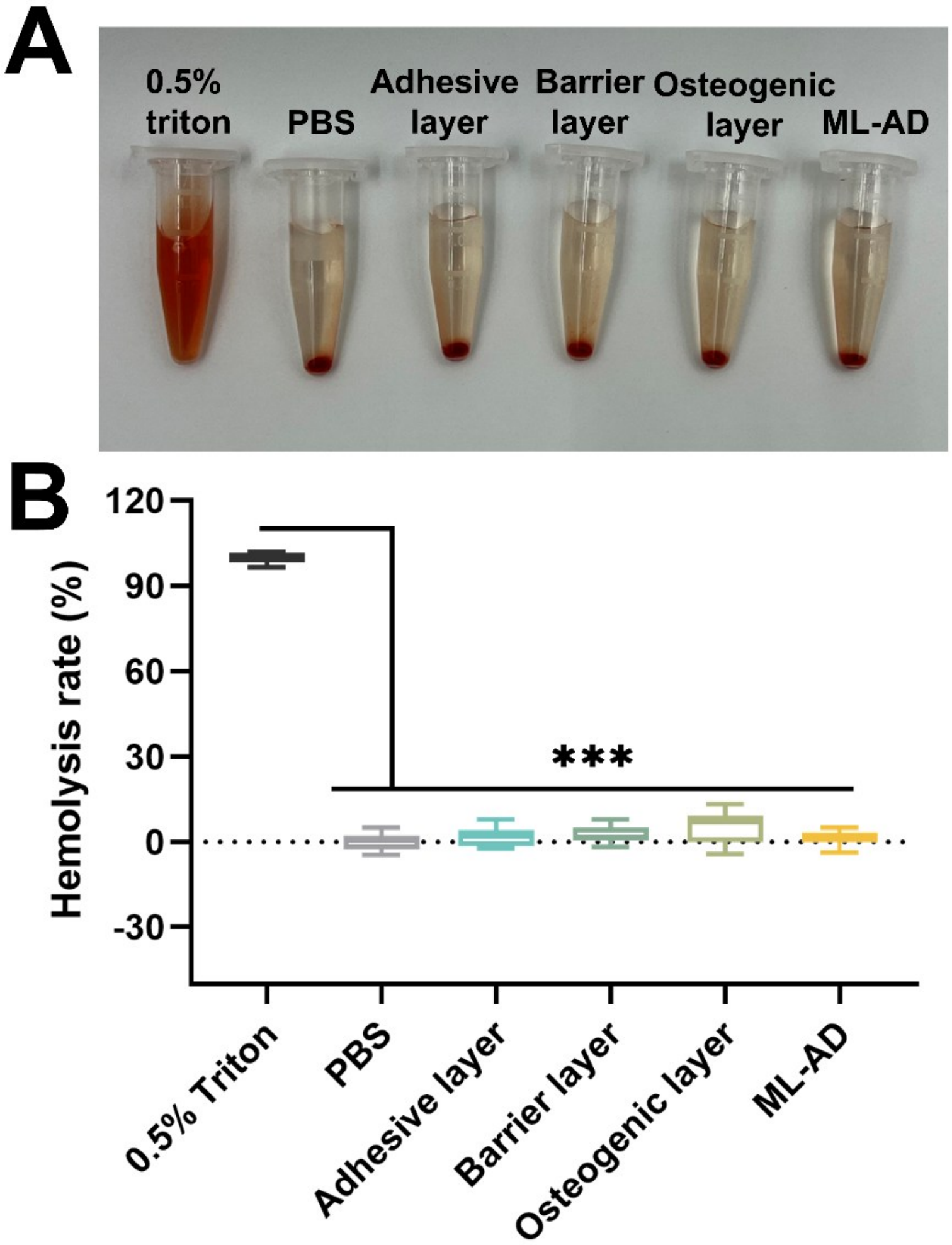

Figure S35. Hemocompatibility of the scaffold. (A) Macroscopic observations of rat red blood cells (RBCs) after incubation with the scaffold and each individual layer followed by centrifugation. (B) Corresponding quantitative hemolysis analysis. Statistical analyses in this figure were performed via one-way ANOVA. ***P < 0.001. N = 3.

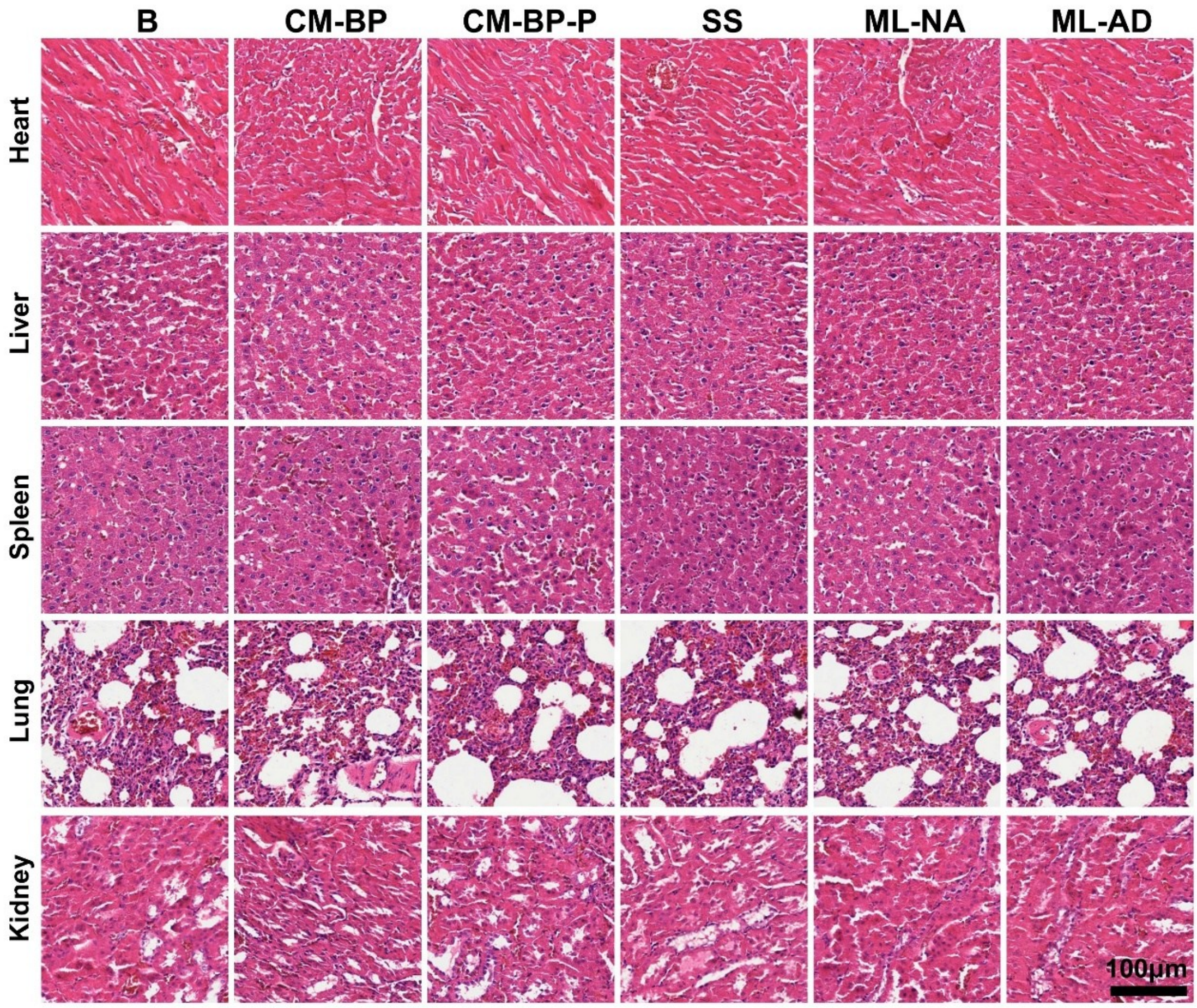

Figure S36. HE staining of heart, liver, spleen, lung, and kidney tissues in each experimental group at 1 month post-operation in rabbits.

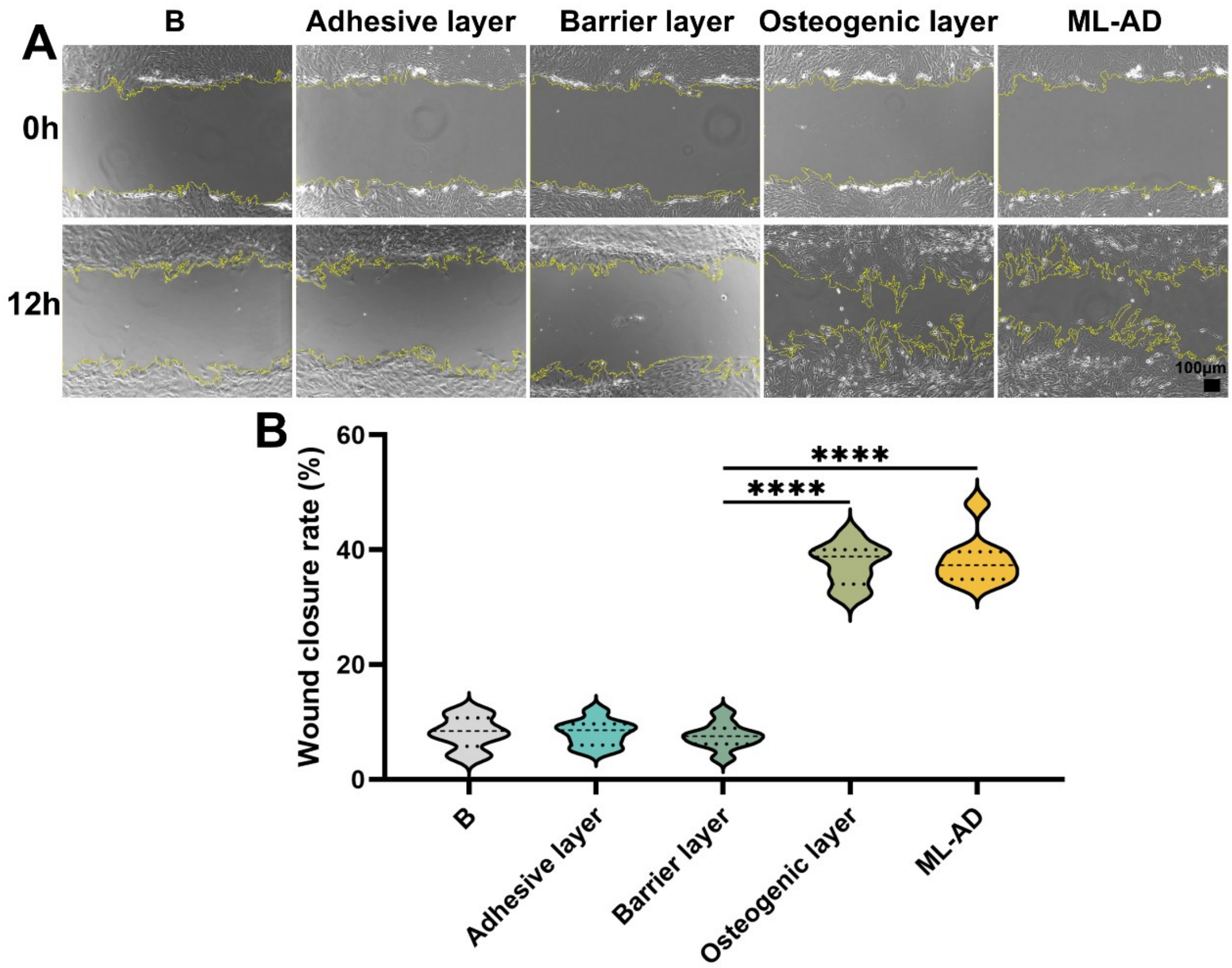

Figure S37. Scratch assay results of BMSCs treated with the extracts of the scaffold and each individual layer for 12 h. (A) Representative images of wound healing in the scratch assay. (B) Quantitative analysis of wound healing determined by the scratch assay. Statistical analyses in this figure were performed via one-way ANOVA. ****$P < 0.0001$. $N = 3$.

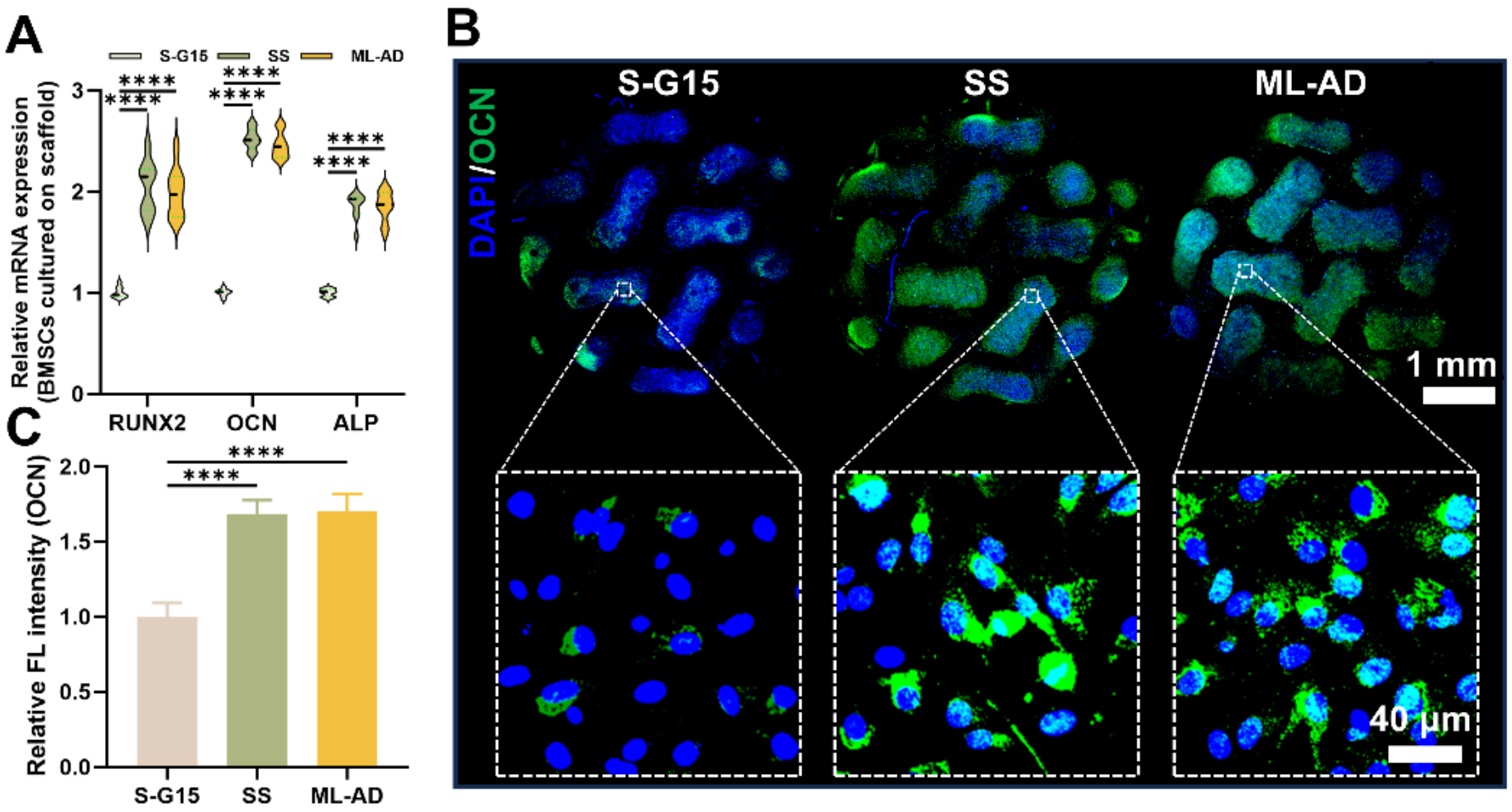

Figure S38. Osteogenic differentiation analysis of BMSCs cultured on the scaffolds for 7 days. (A) Expression analysis of osteogenesis-related genes via RT-qPCR; statistical analyses in this figure were performed via two-way ANOVA. (B) Representative immunofluorescence images of the expression of osteogenesis-related proteins osteocalcin (OCN). (C) Quantitative analysis of the immunofluorescence staining; statistical analyses in this figure were performed via one-way ANOVA. ****P < 0.0001. N = 3.

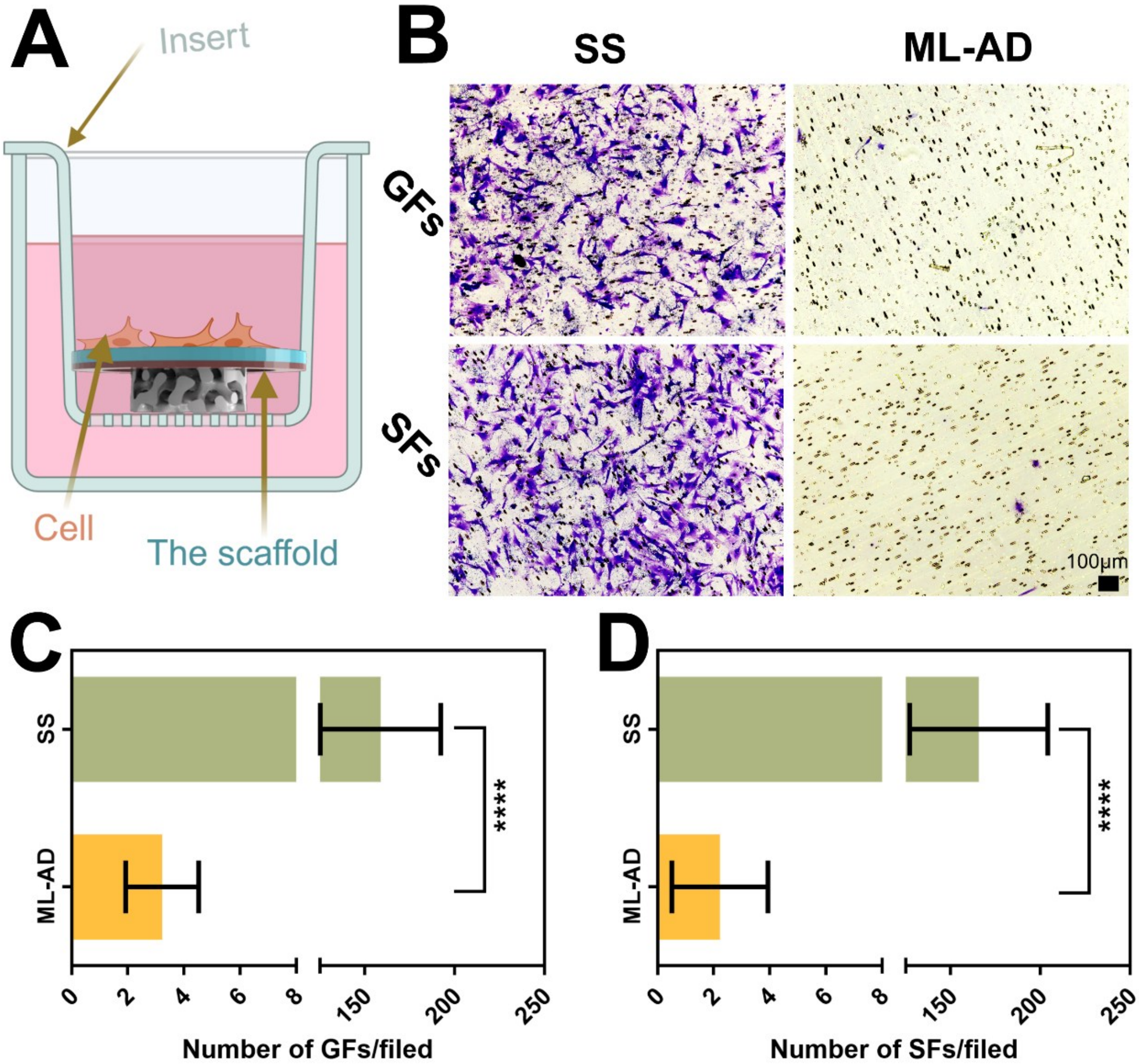

Figure S39. In vitro barrier function test of the scaffold. (A) Schematic diagram of the scaffold placed in the insert. (B) Representative micrographs of insert membrane-transmigrated cells (gingival fibroblasts (GFs) and skin fibroblasts (SFs)) in the lower part of the insert for ML-AD and SS groups. (C) Quantitative analysis of insert membrane-transmigrated GFs. (D) Quantitative analysis of insert membrane-transmigrated SFs. Unpaired Student's t-test was used for statistical analysis in this figure. ****P < 0.0001. N = 3.

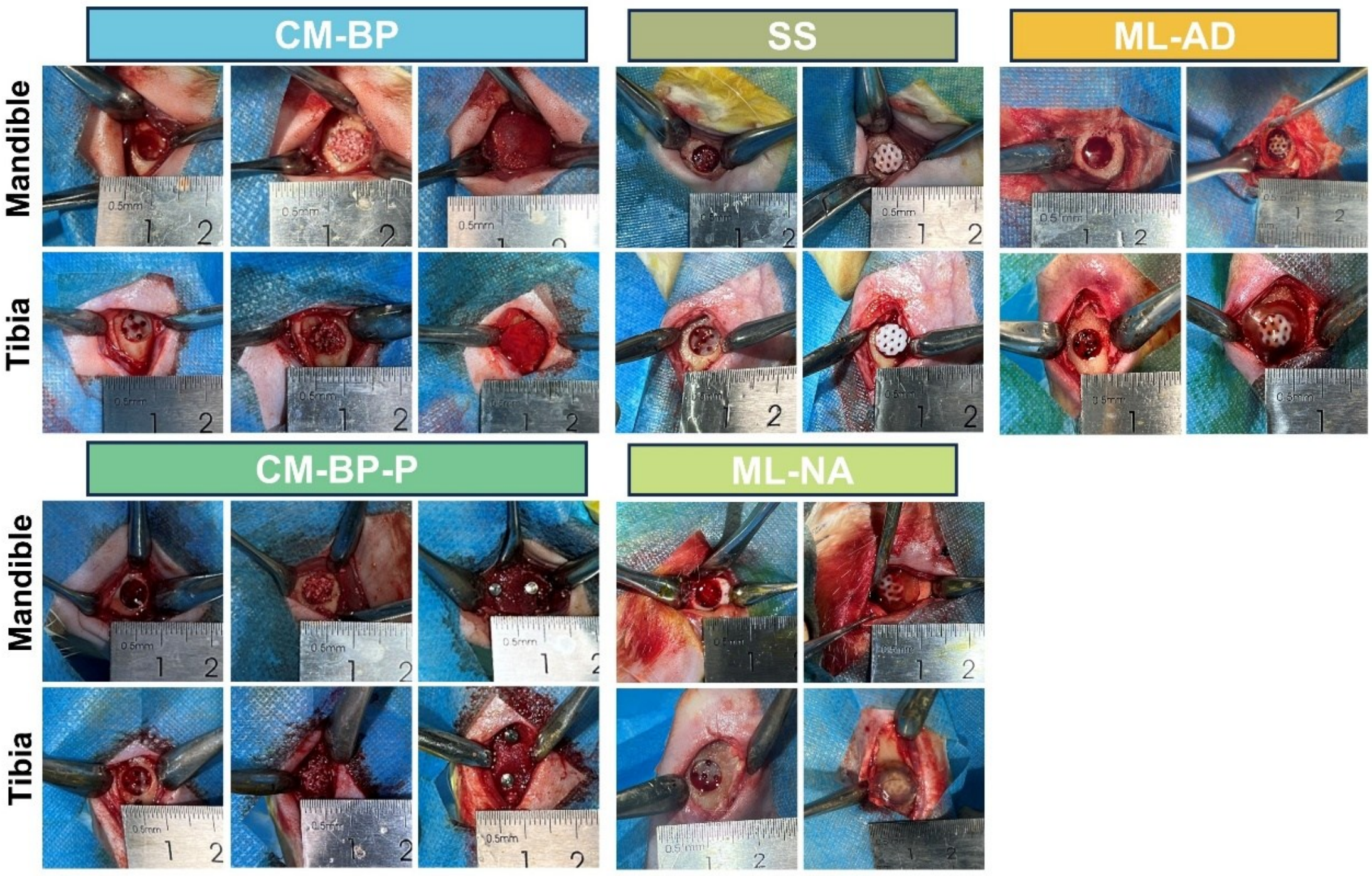

Figure S40. Establishment of rabbit mandible and tibia bone augmentation models and demonstration of the material implantation in each experimental group.

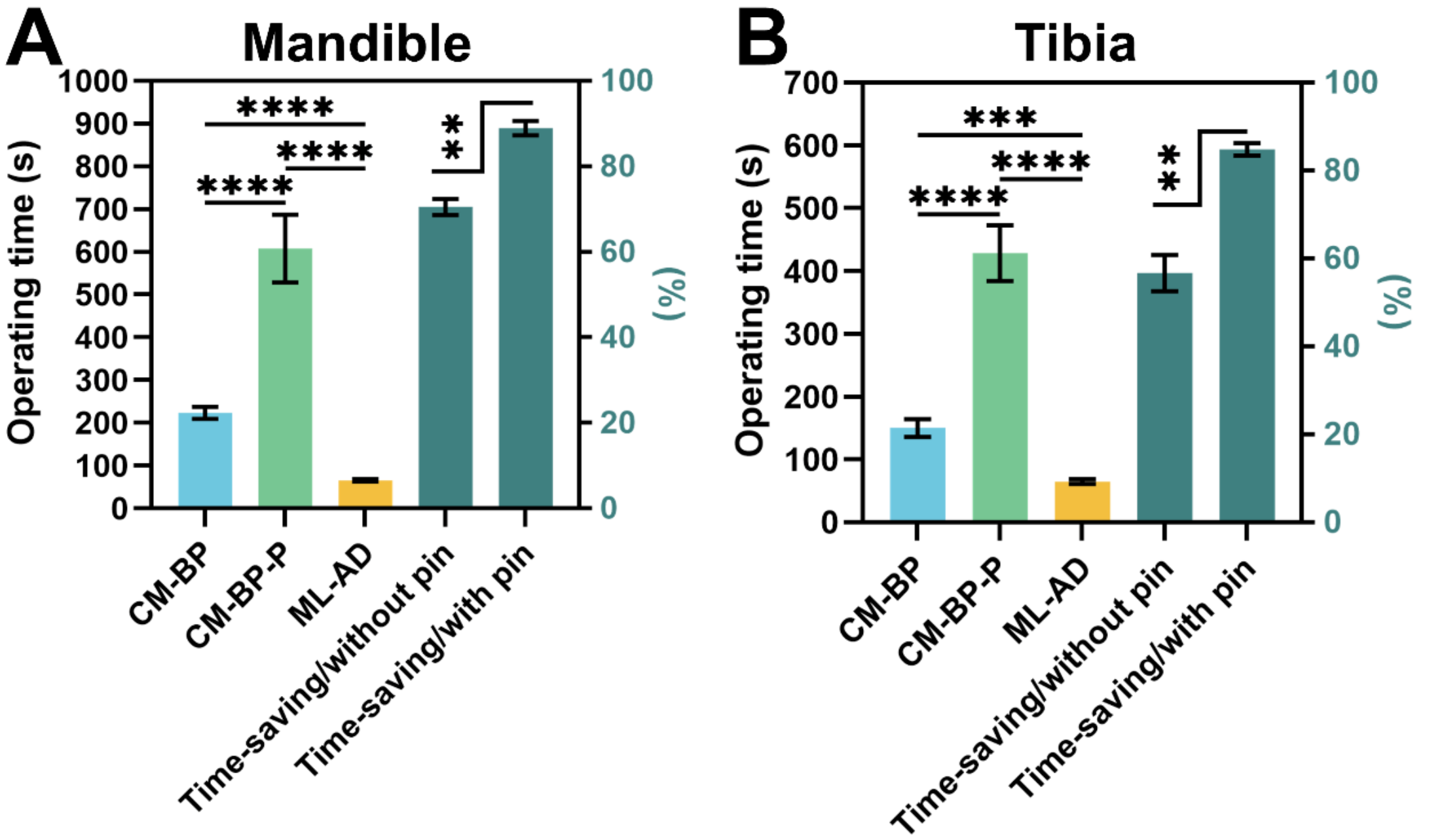

Figure S41. Implantation operation time of scaffolds and materials used in clinical treatment methods for rabbit mandible and tibia bone augmentation models. (A) Implantation operation time of clinical treatment materials (CM-BP and CM-BP-P) and the proposed scaffold (ML-AD) for mandibular bone augmentation, as well as the time saving rate of ML-AD relative to the clinical treatment materials. (B) Operation time for tibial augmentation using clinical materials (CM-BP and CM-BP-P) versus the proposed ML-AD scaffold, together with the corresponding time-saving percentage of ML-AD. Statistical analyses in this figure were performed via one-way ANOVA. **P < 0.01; ****P < 0.0001. N = 4.

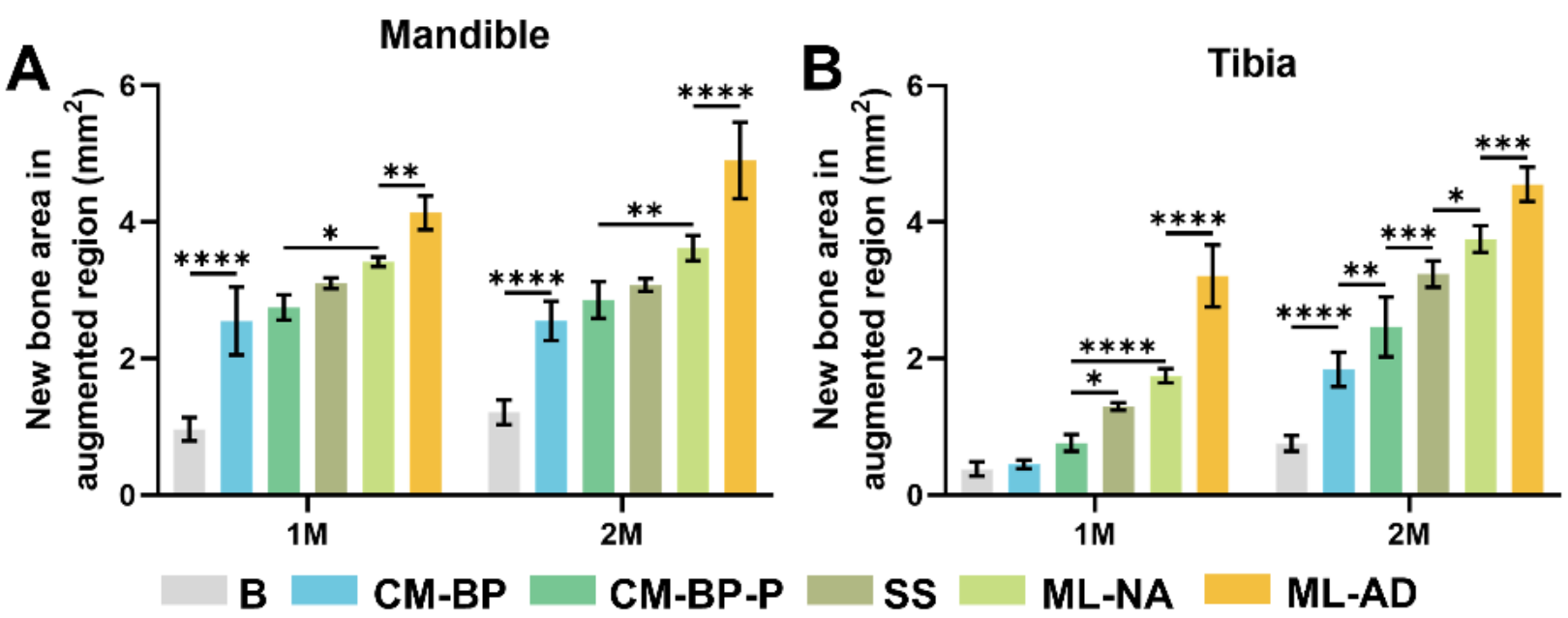

Figure S42. Quantitative analysis of new bone formation in the bone augmentation area based on Goldner staining at 1 and 2 months post-surgery. (A) Mandible and (B) Tibia. Statistical analyses in this figure were performed via two-way ANOVA. *P < 0.05; **P < 0.01; ***P < 0.001; ****P < 0.0001. N = 4.

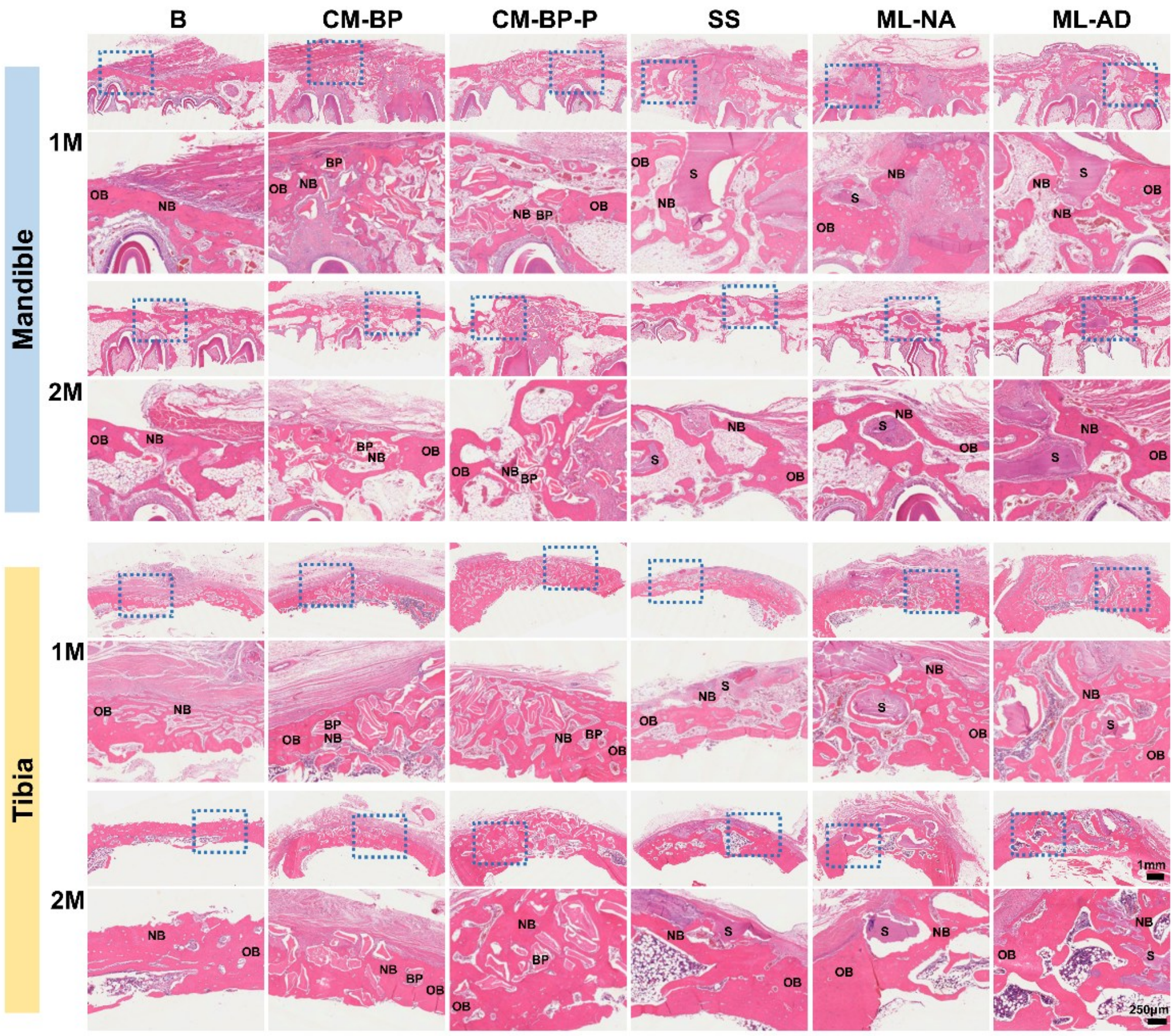

Figure S43. Representative high-magnification HE-stained images (**from Figure 6**) showing the integration between newly formed bone and host bone. OB: old bone; NB: new bone; BP: bone powder; S: scaffold.

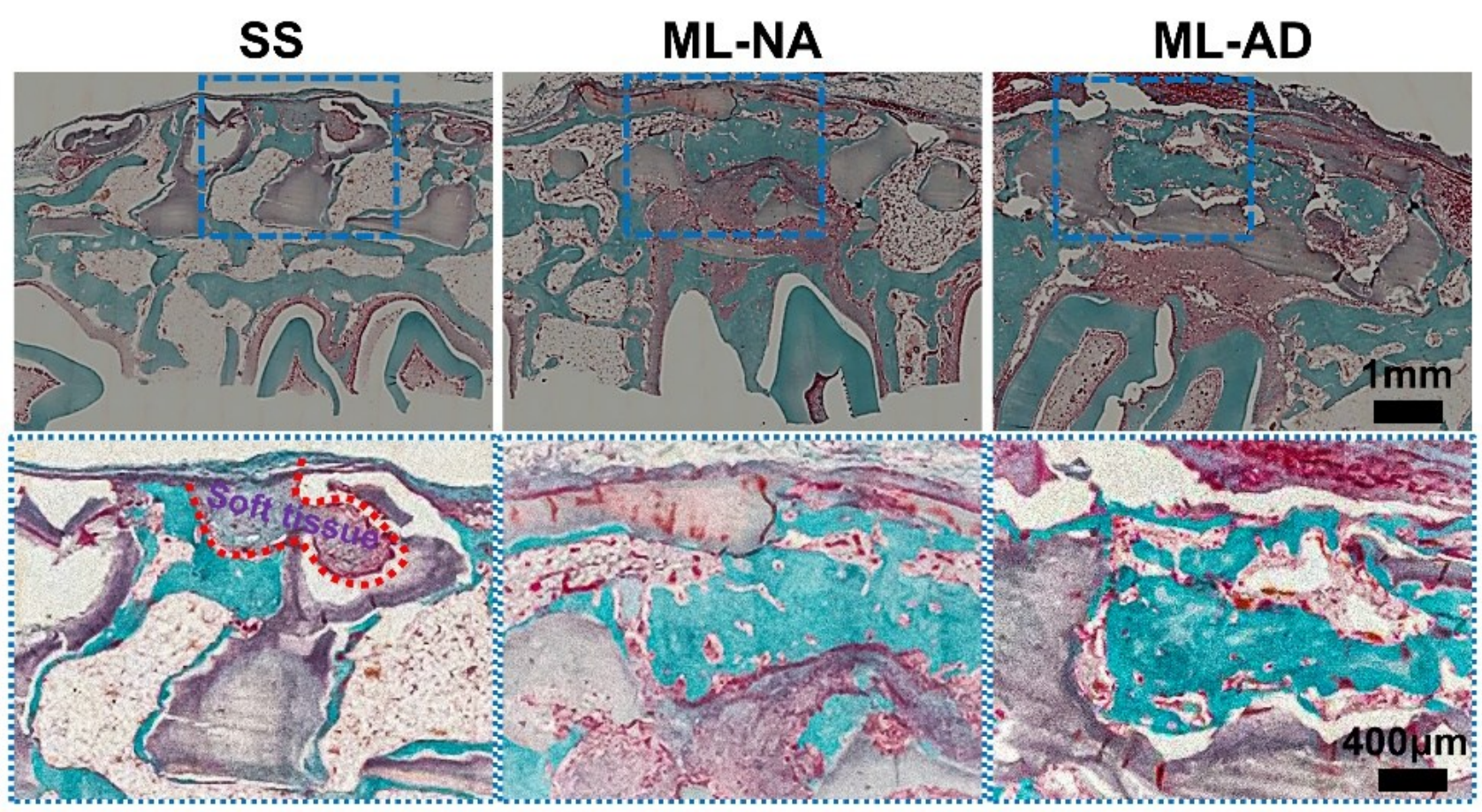

Figure S44. Goldner-stained sections at 1 month post-operatively showed evident soft tissue ingrowth into the scaffold interior in the SS group.

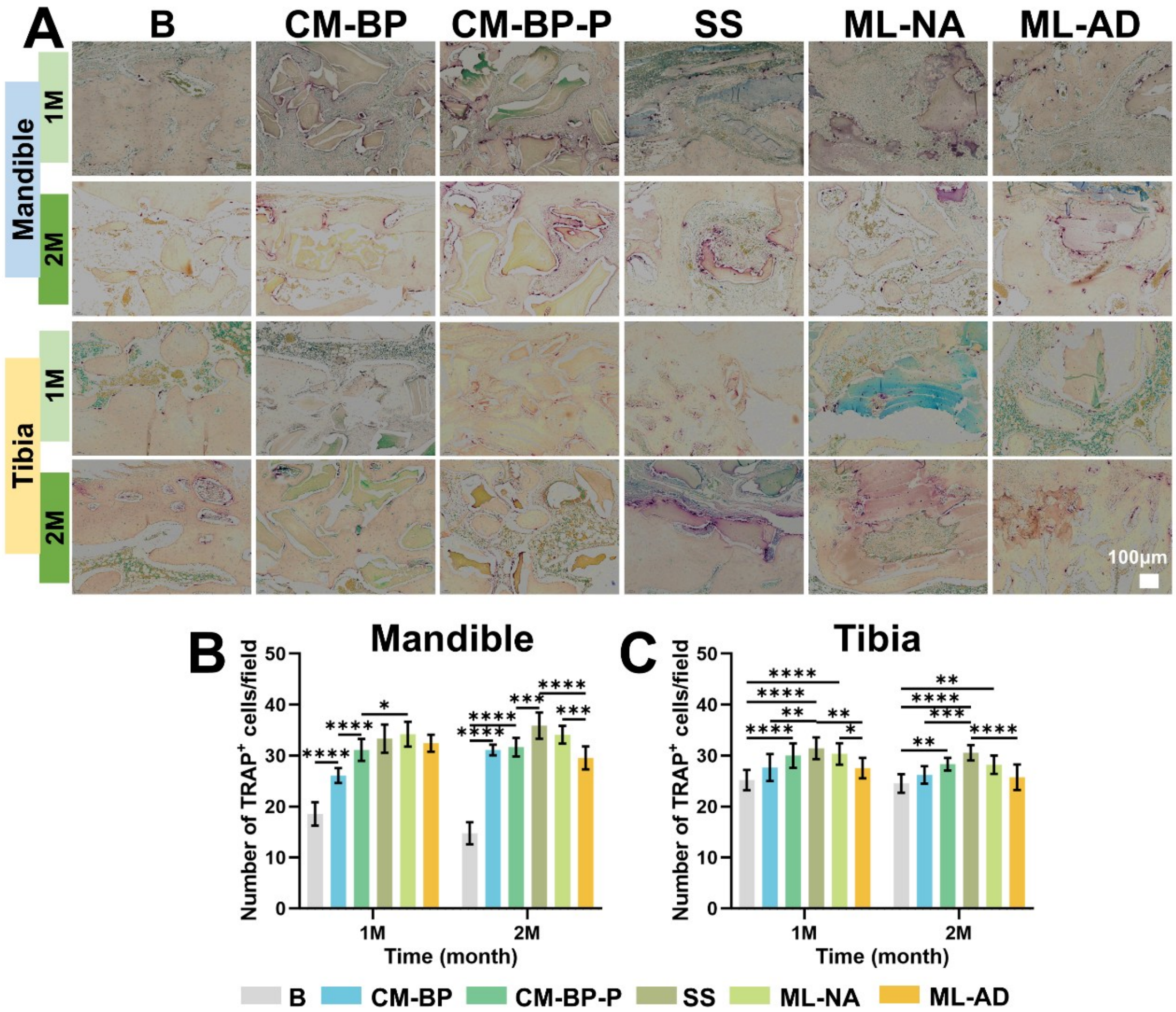

Figure S45. TRAP staining and quantitative analysis in rabbit mandible and tibia at 1 and 2 months post-operation. (A) Representative TRAP staining images of the bone augmentation regions in rabbit mandible and tibia, corresponding to 1 month and 2 months post-operation. (B, C) Quantitative analysis of the number of TRAP-positive ($TRAP^+$) cells per field in the bone augmentation regions of rabbit mandible (B) and tibia (C) at 1 and 2 months post-operation. Statistical analyses in this figure were performed via two-way ANOVA. *$P < 0.05$; **$P < 0.01$; ***$P < 0.001$; ****$P < 0.0001$. N = 4.

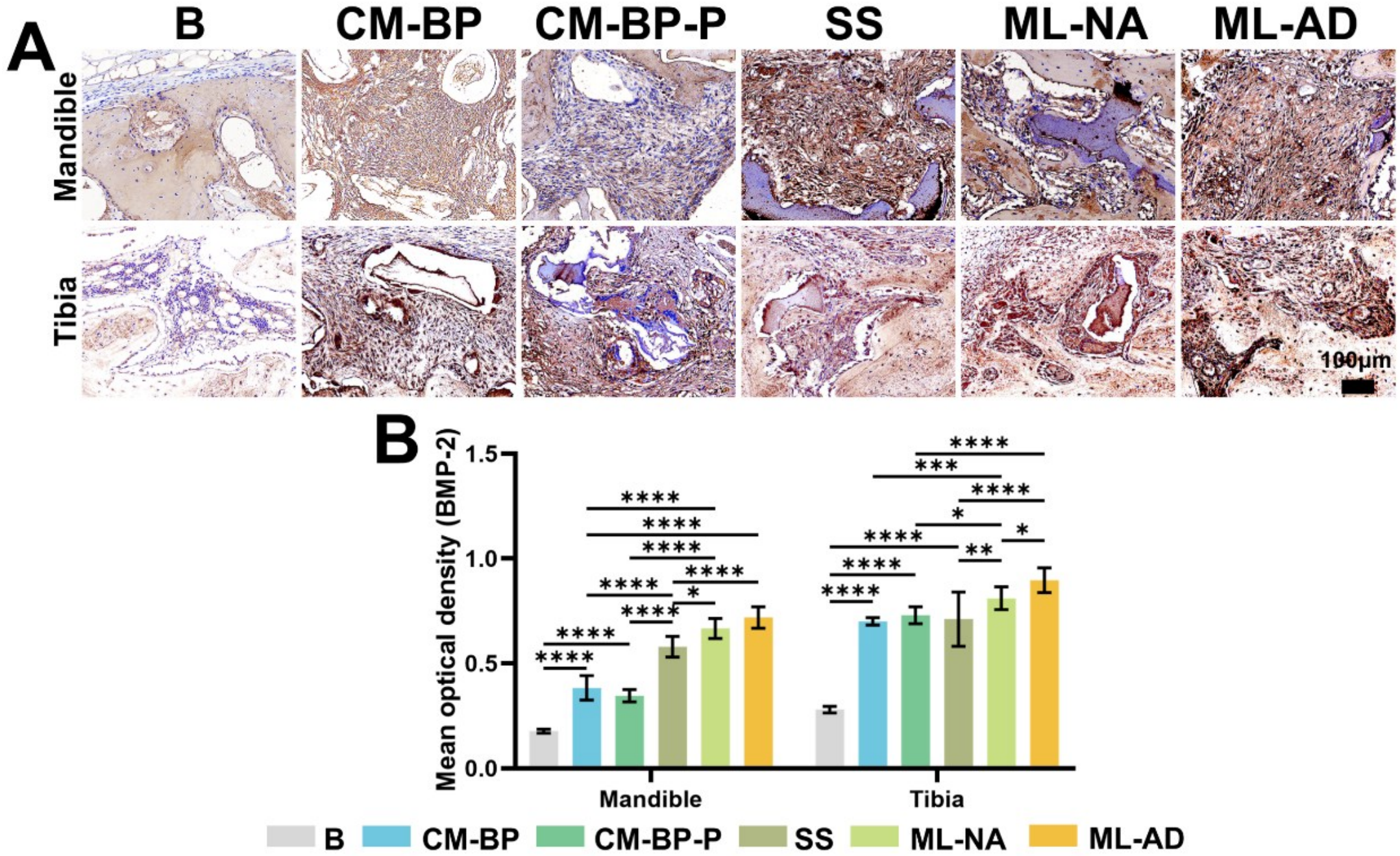

Figure S46. Immunohistochemical (IHC) staining of BMP-2. (A) Representative IHC staining images of BMP-2 in the bone augmentation regions of rabbit mandible and tibia at 2 months post-operation. (B) Corresponding quantitative analysis of BMP-2 IHC staining. Statistical analyses in this figure were performed via two-way ANOVA. *P < 0.05; **P < 0.01; ***P < 0.001; ****P < 0.0001. N = 4.

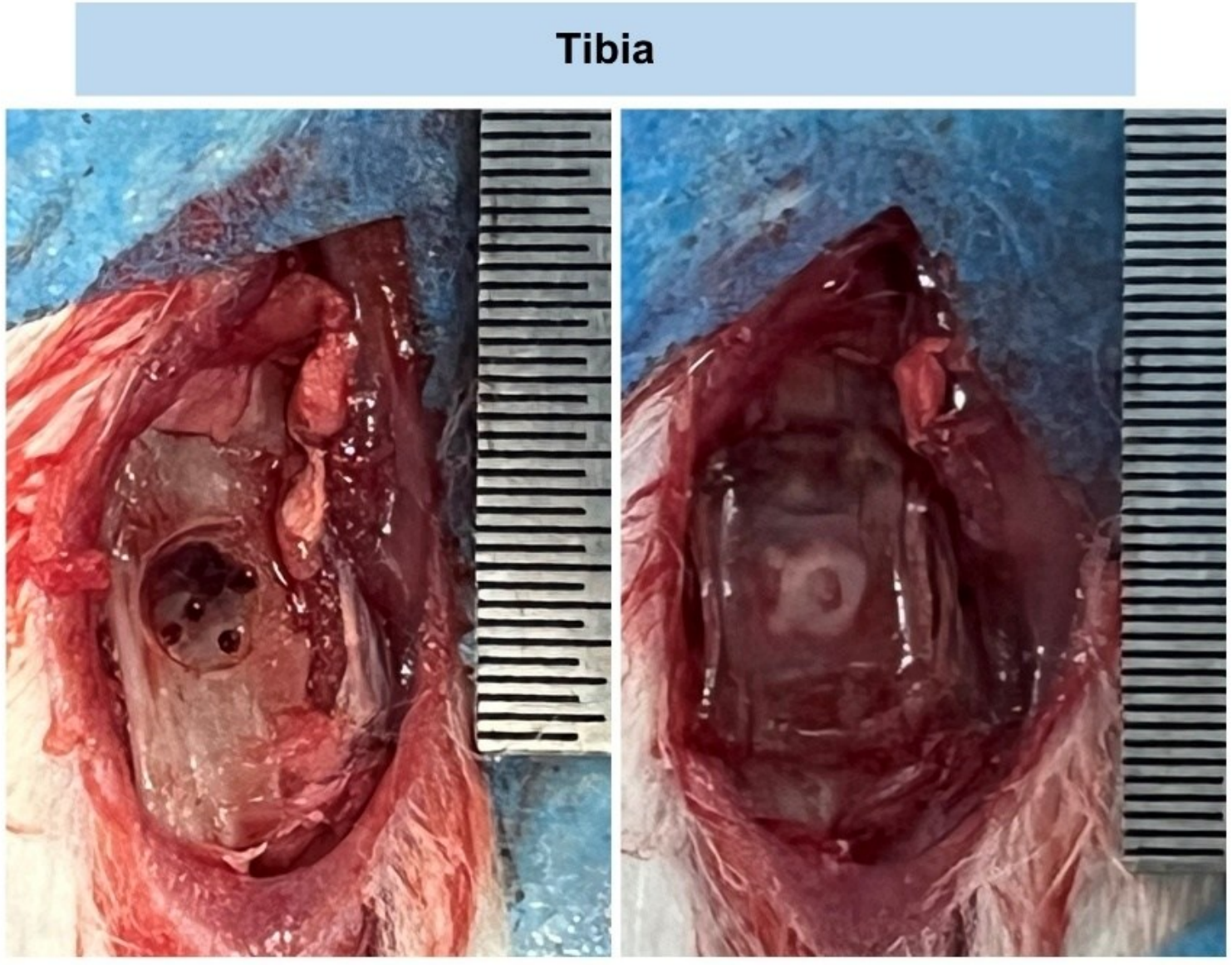

Figure S47. Establishment of tibial bone augmentation model in Wistar rats and demonstration of the scaffold implantation.

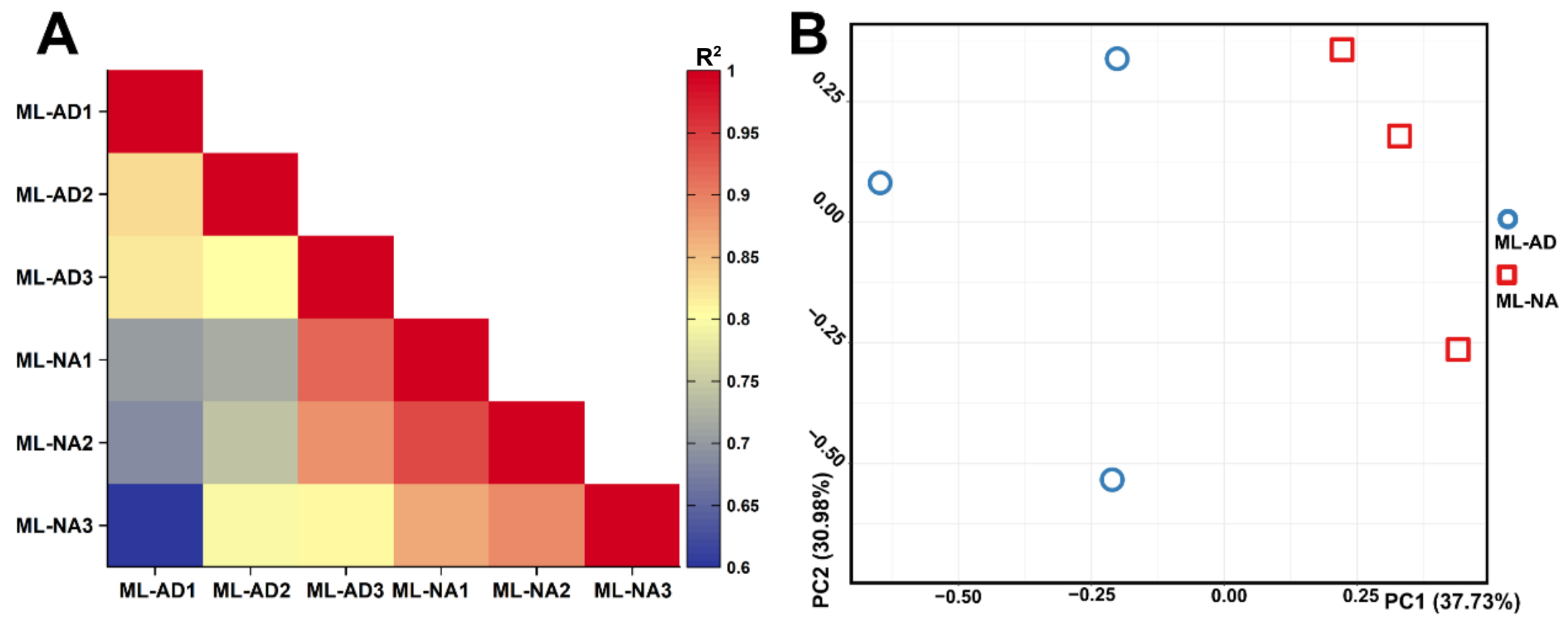

Figure S48. Sample-to-sample correlation analysis of RNA sequencing (RNA-seq) data from rats at 7 days post-operation. (A) Correlation heatmap of FPKM expression levels among samples; color intensity indicates the correlation coefficient (higher intensity represents stronger correlation). (B) Principal component analysis (PCA) plot of FPKM expression levels; each dot represents an individual sample, and group information is indicated by colors and shapes, as per the figure annotation.

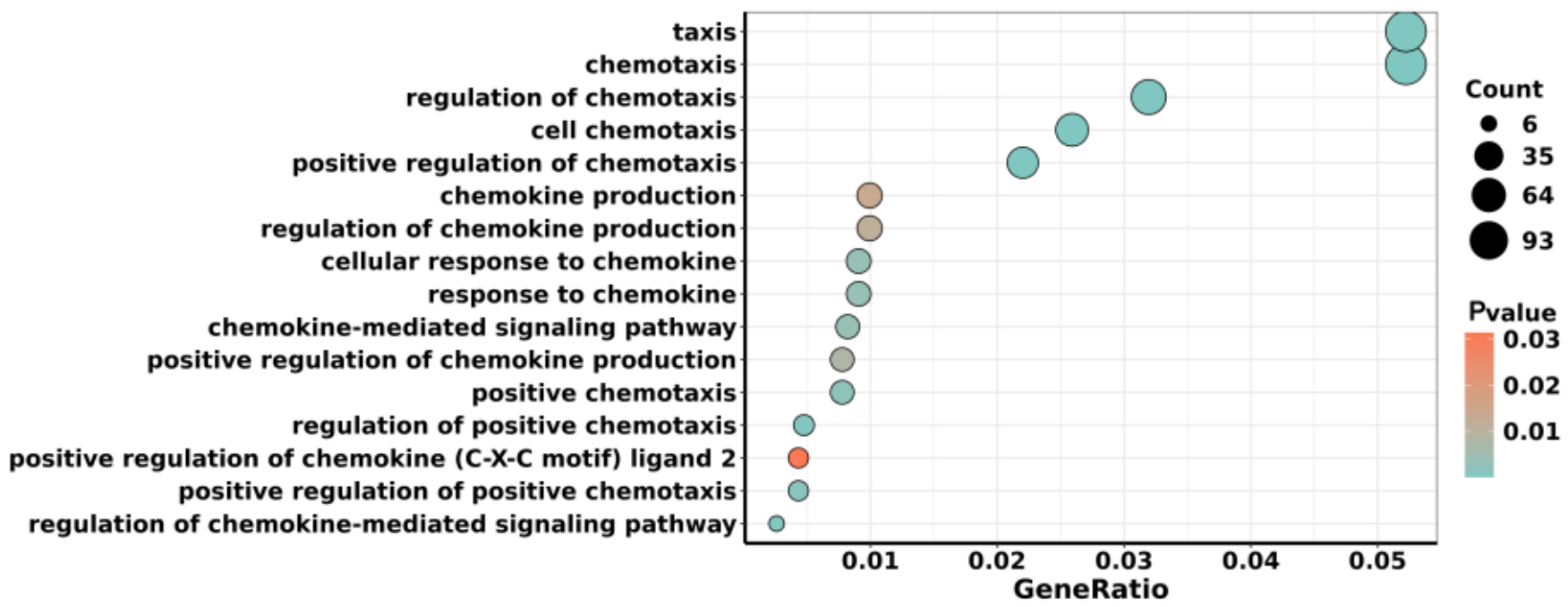

Figure S49. Bubble chart of GO-BP enrichment analysis for DEGs associated with chemotaxis response between ML-AD and ML-NA.

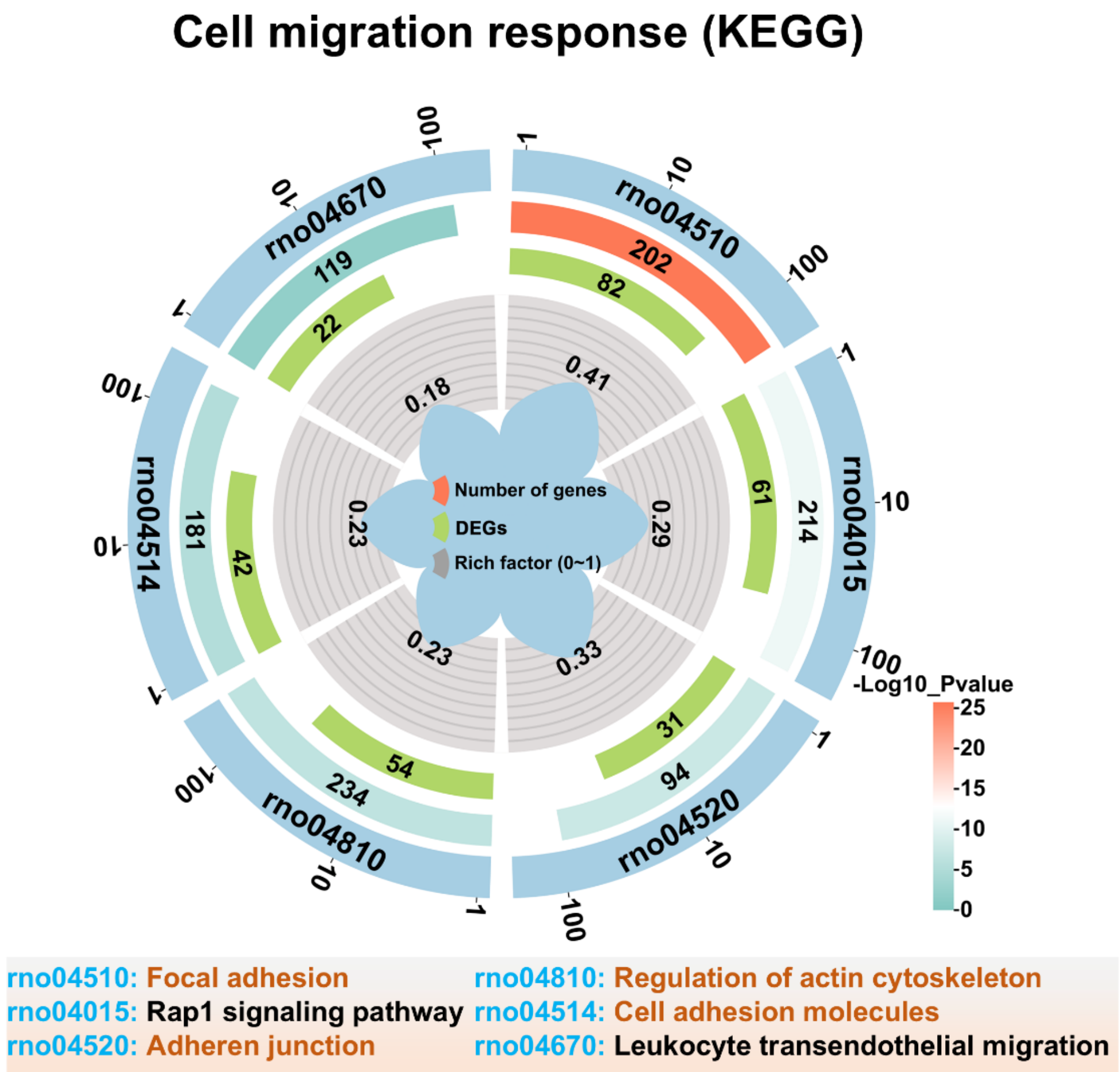

Figure S50. KEGG enrichment circle plot of DEGs associated with cell migration response from RNA-seq data of rats at 7 days post-operation.

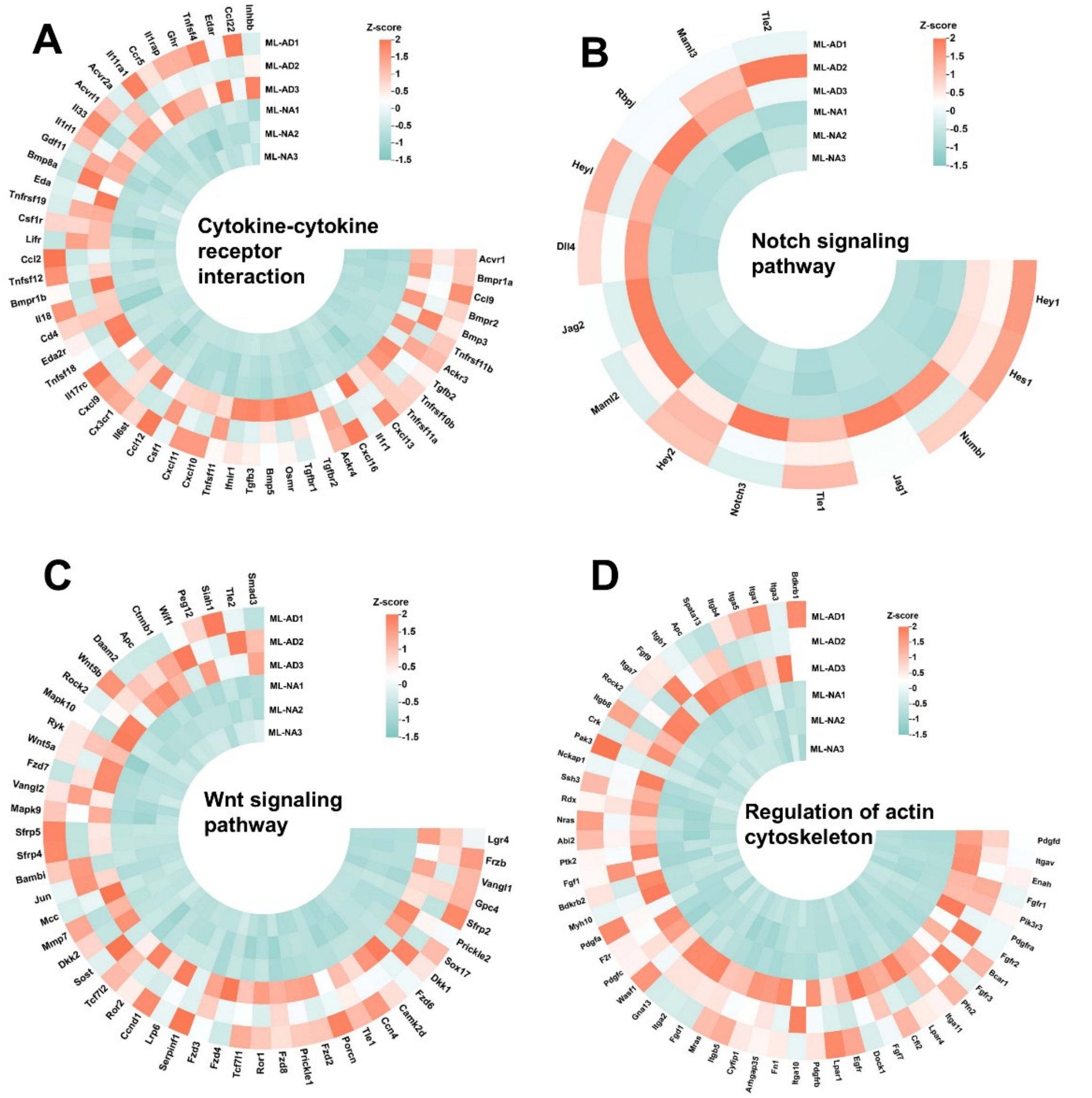

Figure S51. Circular heatmaps of DEGs in KEGG pathways associated with inflammation response, angiogenesis response, osteogenesis response, and cell migration response between ML-NA and ML-AD groups. (A) Circular heatmap of significantly DEGs in the inflammation-related Cytokine-Cytokine Receptor Interaction pathway. (B) Circular heatmap of significantly DEGs in the angiogenesis-related Notch Signaling Pathway. (C) Circular heatmap of significantly DEGs in the osteogenesis-related Wnt Signaling Pathway. (D) Circular heatmap of significantly DEGs in the cell migration-related Regulation of Actin Cytoskeleton pathway.

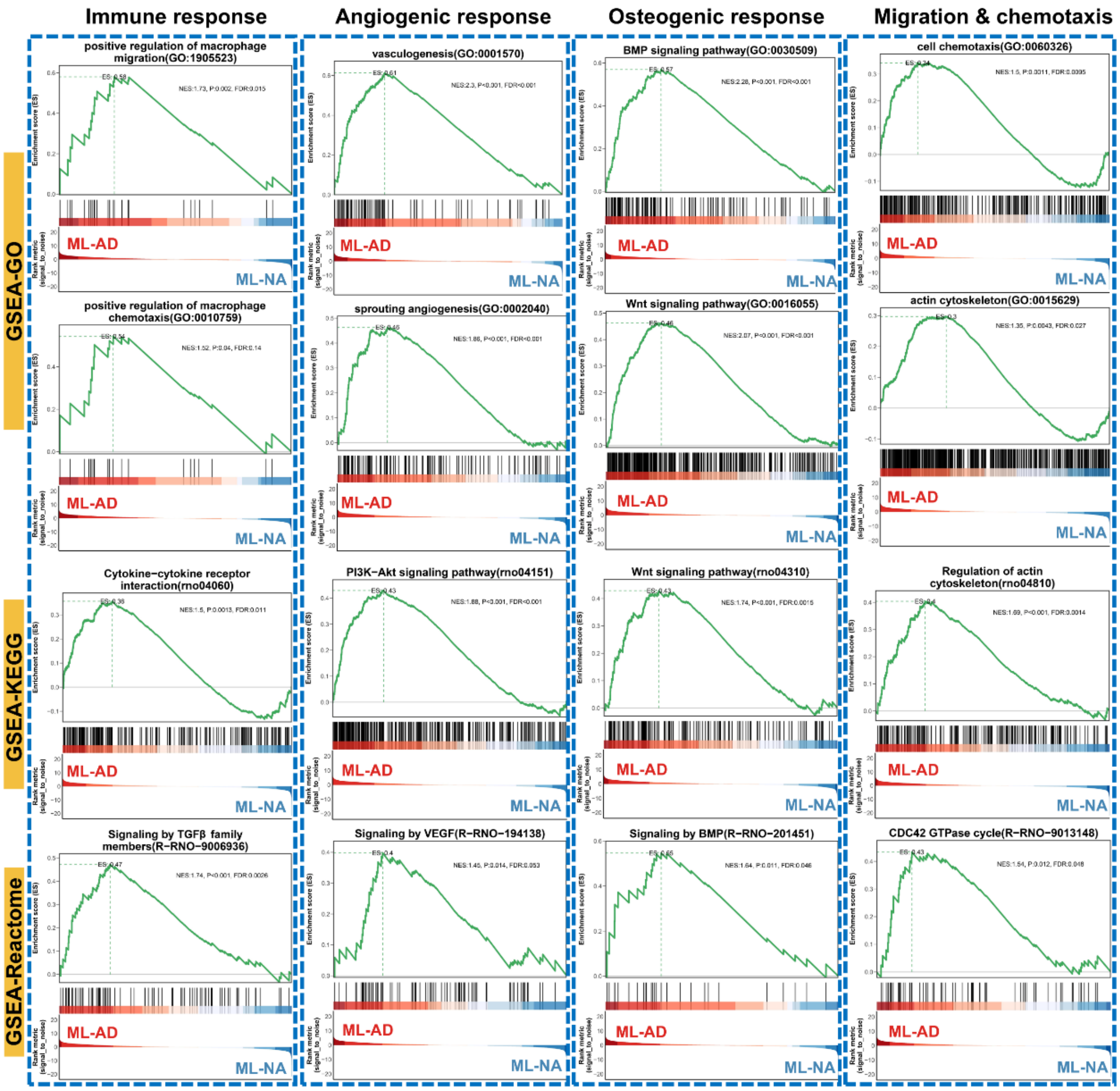

Figure S52. Gene Set Enrichment Analysis (GSEA) based on RNA-sequencing at 7 days postoperatively in rats, including inflammatory response, angiogenic response, osteogenic response, and cell migration response.

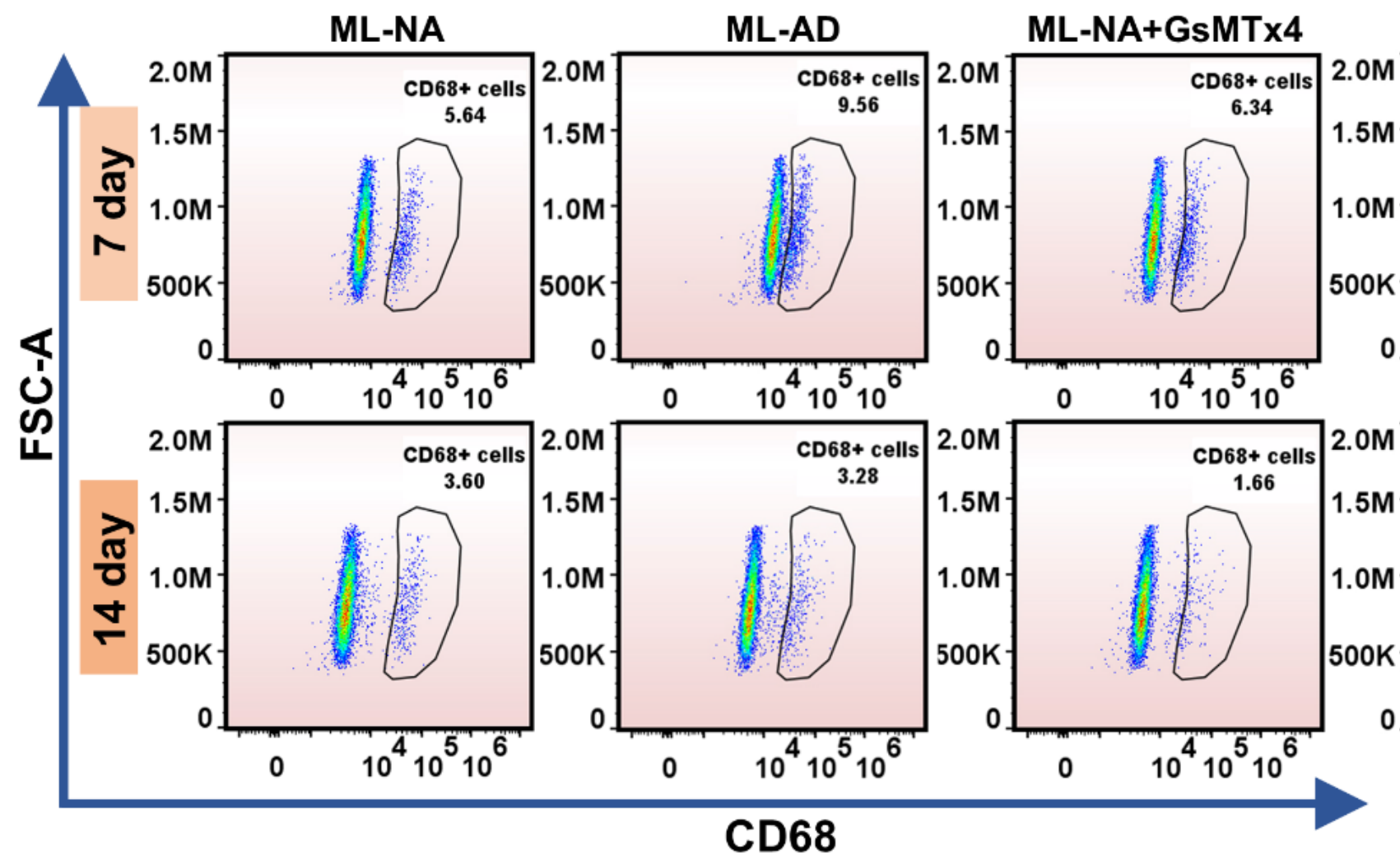

Figure S53. Flow cytometry analysis of CD68^{+} cells proportion within the scaffold at 7 and 14 days postoperatively.

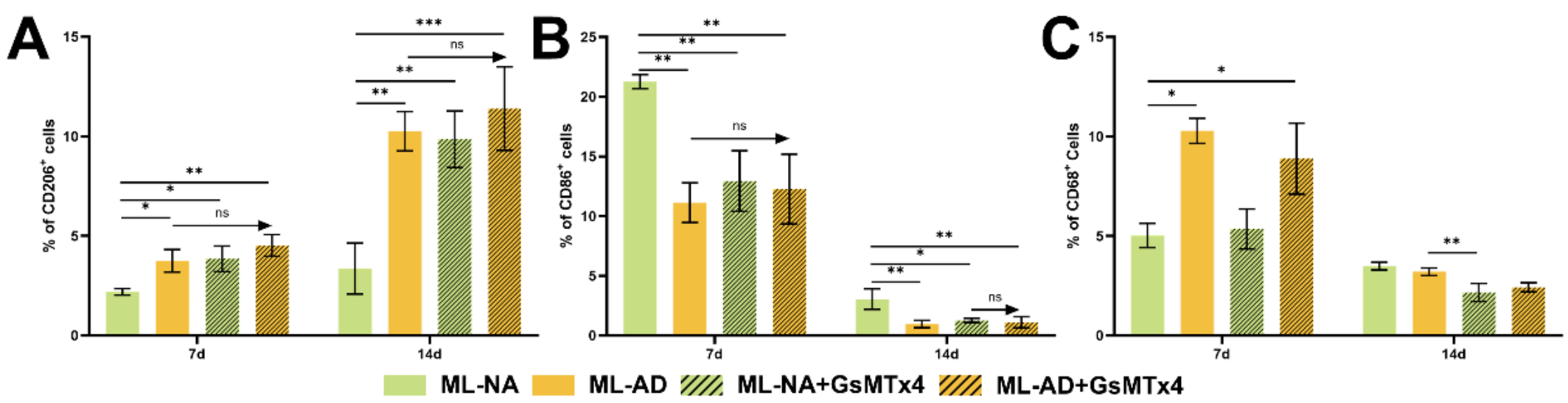

Figure S54. Flow cytometry quantitative analysis of macrophages within the scaffold at 7 and 14 days postoperatively. (A) Quantitative assessment of CD68^{+} cells proportion. (B) M1-like macrophage proportion (CD68^{+}CD86^{+} cells). (C) M2-like macrophage proportion (CD68^{+}CD206^{+} cells). Statistical analyses in this figure were performed via one-way ANOVA. *P < 0.05; **P < 0.01; ***P < 0.001. N = 3.

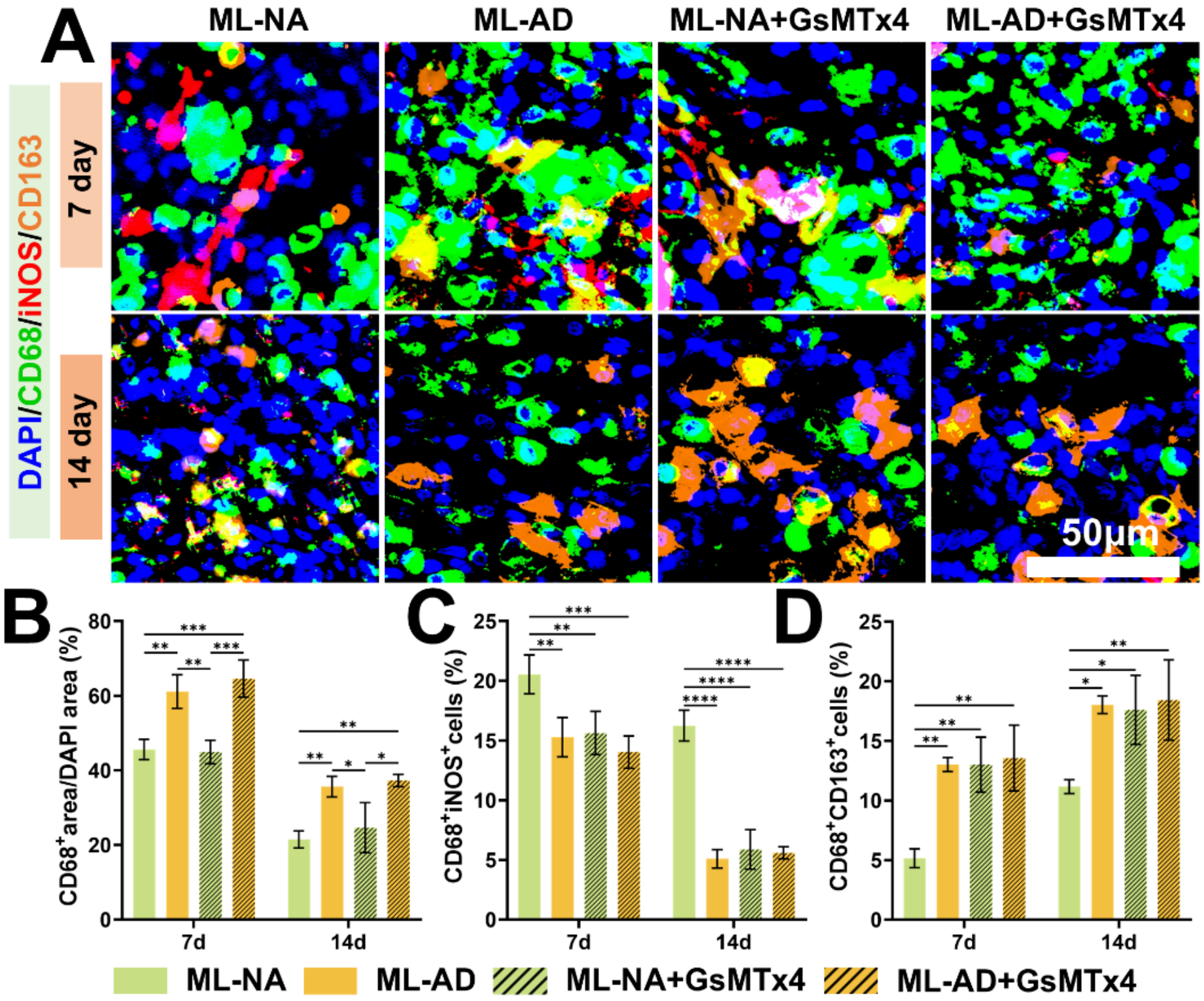

Figure S55. Immunofluorescence staining of the osteogenic region in rats at 7 and 14 days postoperatively. (A) Representative immunofluorescence staining images (CD68, CD163, and iNOS). (B-D) Semi-quantitative analyses including $CD68^+$ area, $CD68^+iNOS^+$ double-positive cells, and $CD68^+CD163^+$ double-positive cells. Statistical analyses in this figure were performed via two-way ANOVA. *P < 0.05; **P < 0.01; ***P < 0.001; ****P < 0.0001. N = 3.

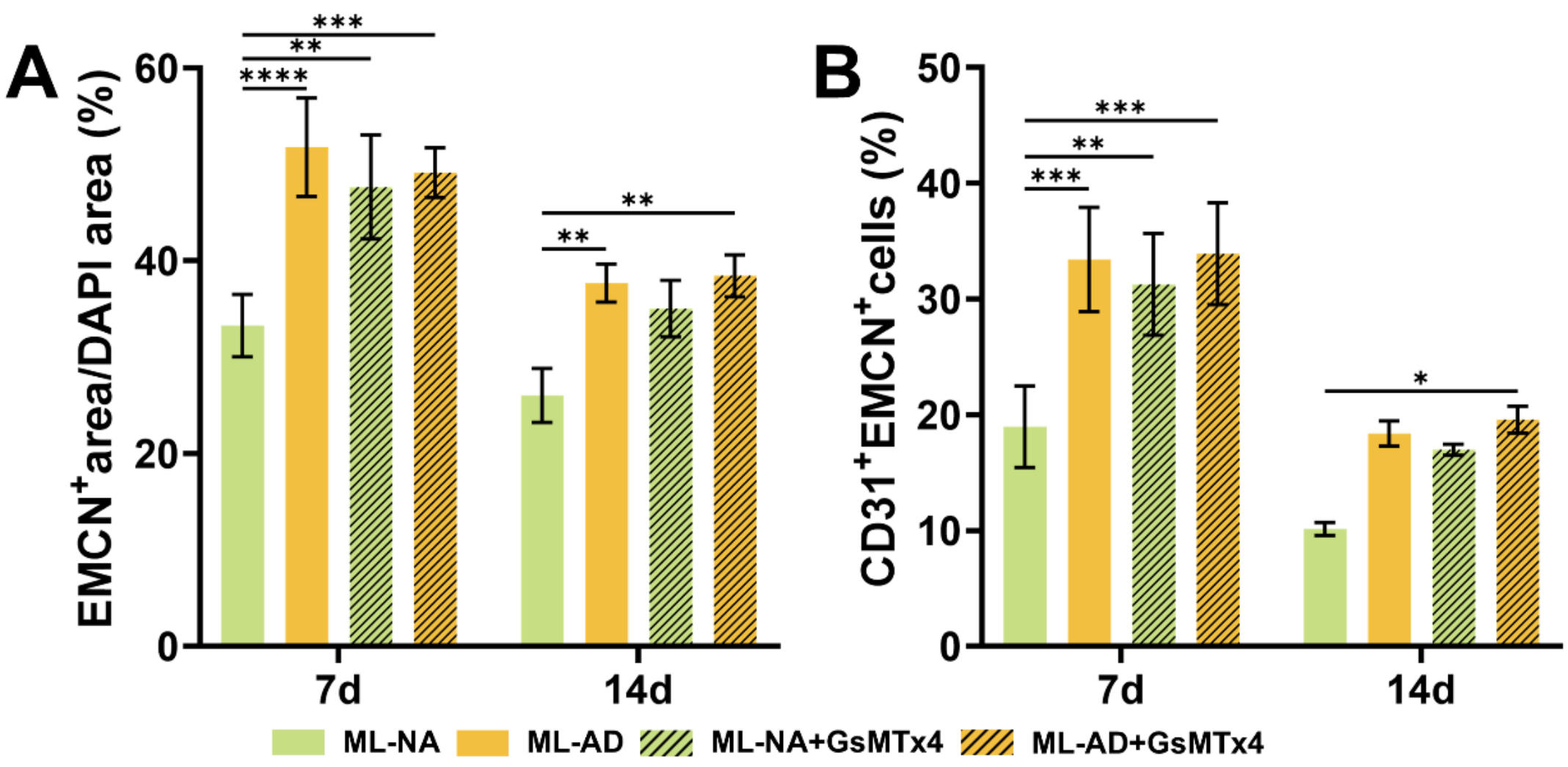

Figure S56. Semi-quantitative analysis of immunofluorescence staining for EMCN^{+} area (A) and CD31^{+}EMCN^{+} double-positive cells (B) in the osteogenic zone of rats at 7 and 14 days postoperatively. Statistical analyses in this figure were performed via two-way ANOVA. *P < 0.05; **P < 0.01; ***P < 0.001; ****P < 0.0001. N = 3.

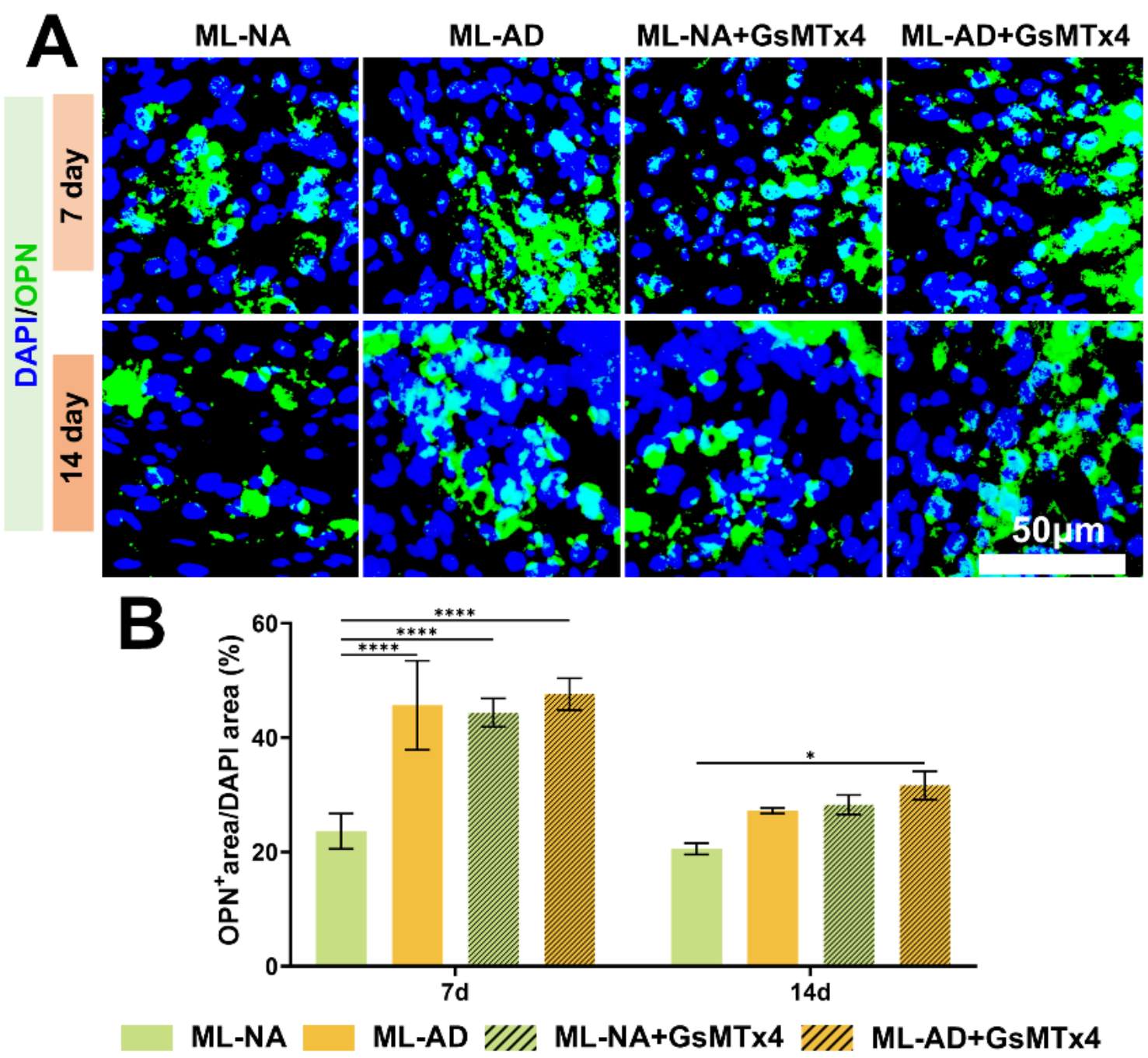

Figure S57. Immunofluorescence staining of the osteogenic region in rats at 7 and 14 days postoperatively. (A) Representative immunofluorescence staining images (OPN). (B) Semi-quantitative analysis of the OPN^{+} area. Statistical analyses in this figure were performed via two-way ANOVA. *P < 0.05; ****P < 0.0001. N = 3.

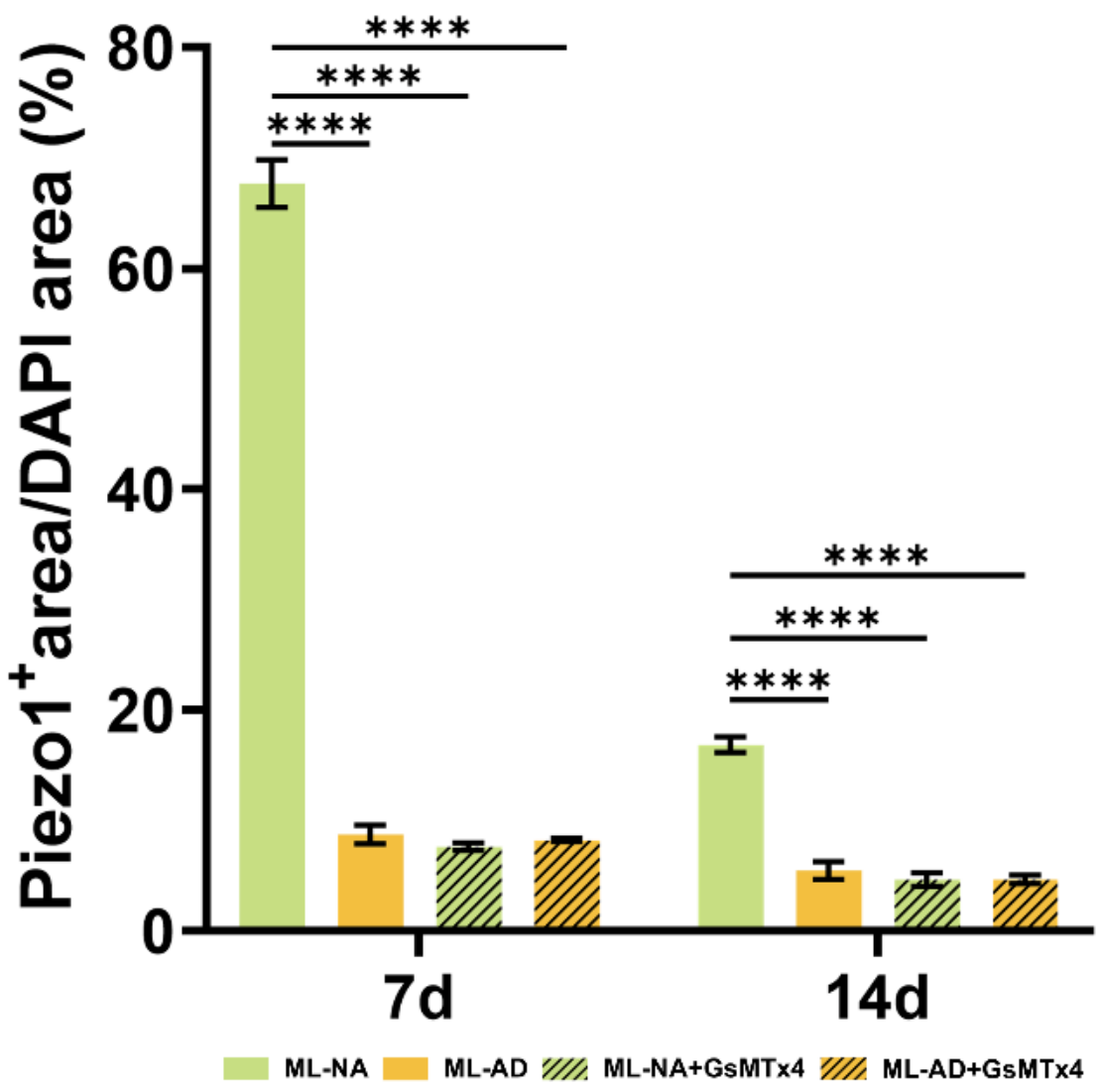

Figure S58. Semi-quantitative analysis of immunofluorescence staining for the Piezo1^{+} area in the osteogenic region of rats at 7 and 14 days postoperatively. Statistical analyses in this figure were performed via two-way ANOVA. ****P < 0.0001. N = 3.

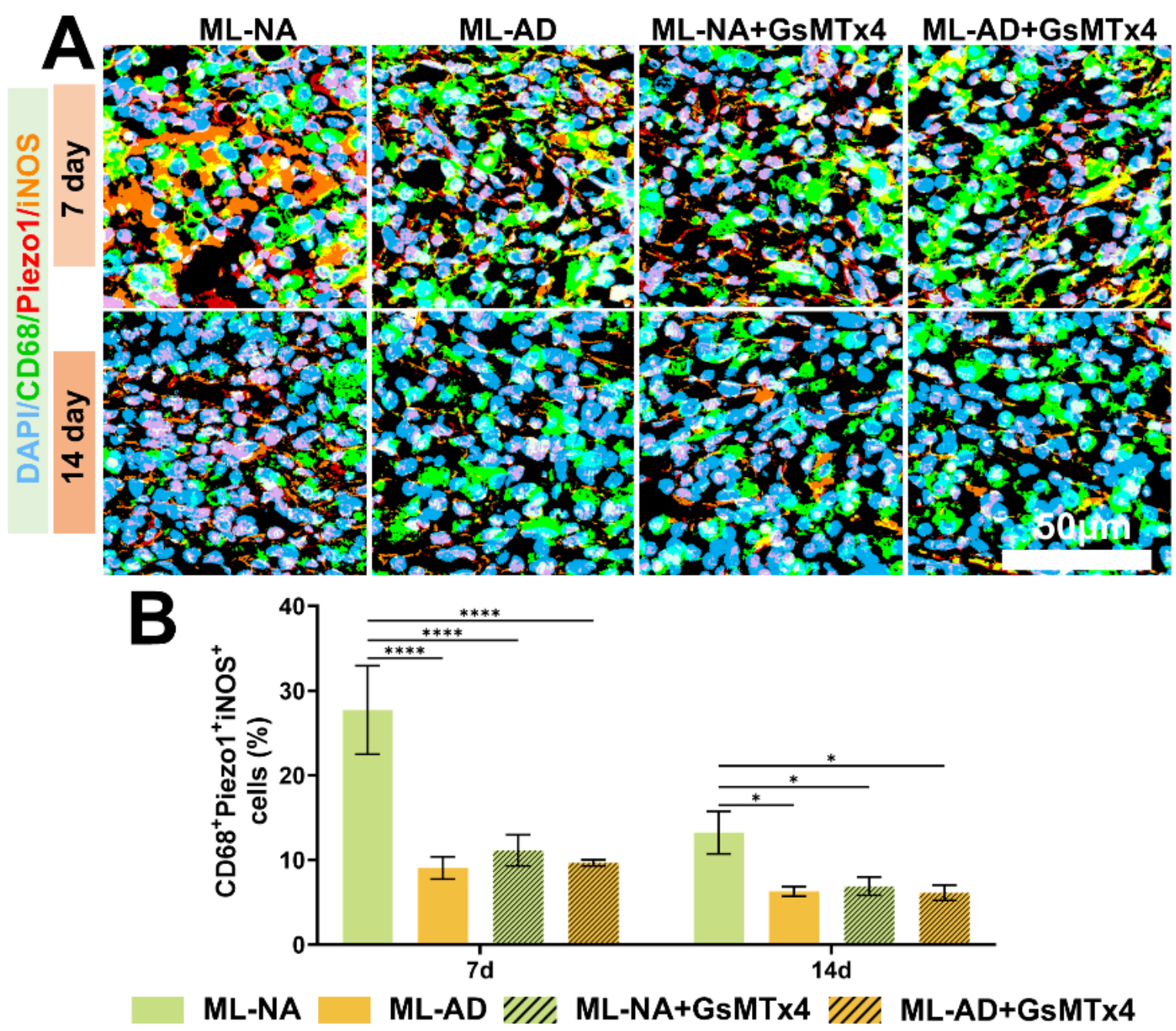

Figure S59. Immunofluorescence staining of the osteogenic region in rats at 7 and 14 days postoperatively. (A) Representative immunofluorescence staining images (CD68, Piezo1, and iNOS). (B) Semi-quantitative analysis of CD68^{+}Piezo1^{+}iNOS^{+} triple-positive cells. Statistical analyses in this figure were performed via two-way ANOVA. *P < 0.05; ****P < 0.0001. N = 3.

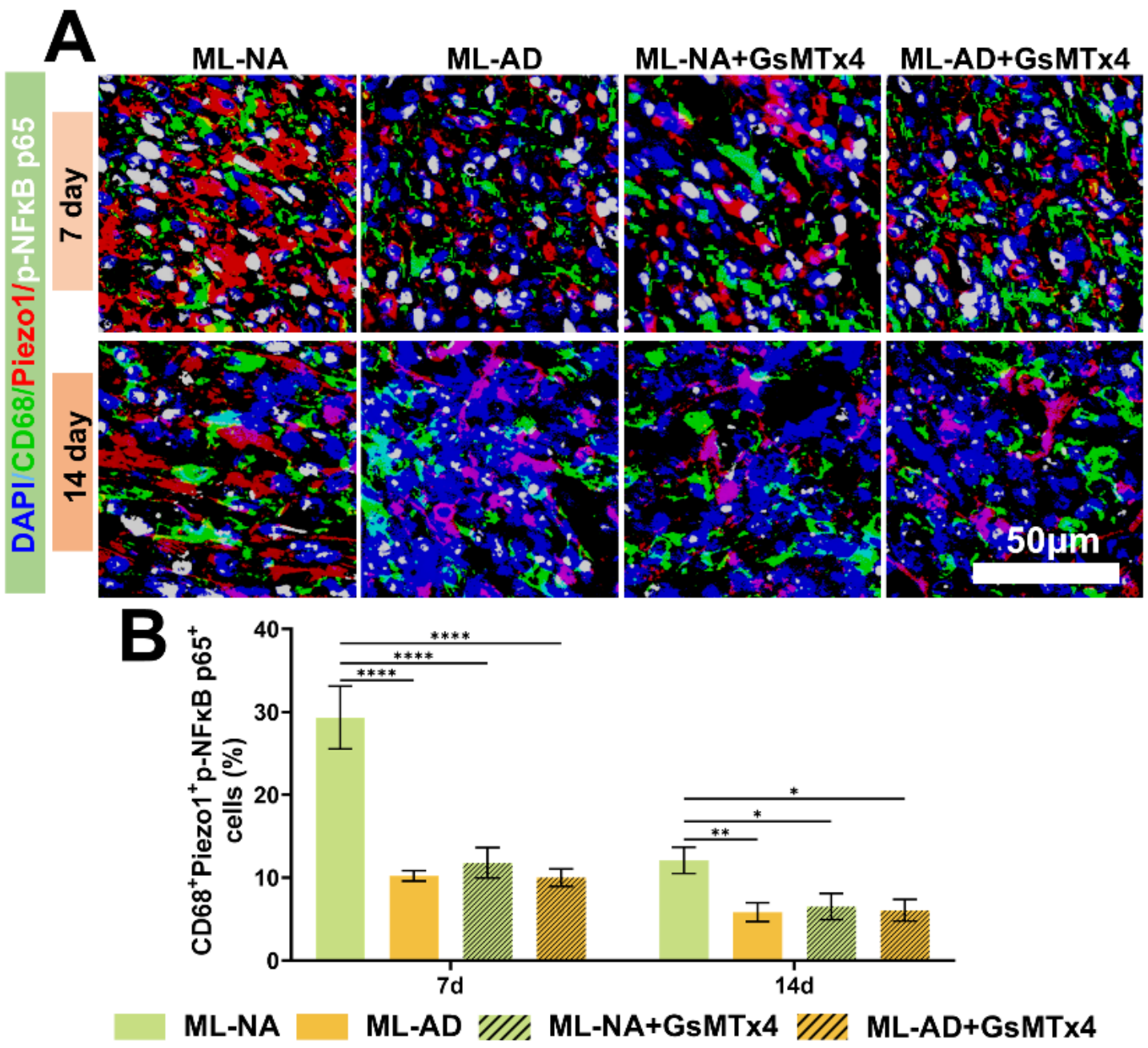

Figure S60. Immunofluorescence staining of the osteogenic region in rats at 7 and 14 days postoperatively. (A) Representative immunofluorescence staining images (CD68, Piezo1, and p-NFκB p65). (B) Semi-quantitative analysis of $CD68^+Piezo1^+$p-NFκB $p65^+$ triple-positive cells. Statistical analyses in this figure were performed via two-way ANOVA. $^*P < 0.05$; $^{**}P < 0.01$; $^{****}P < 0.0001$. N = 3.

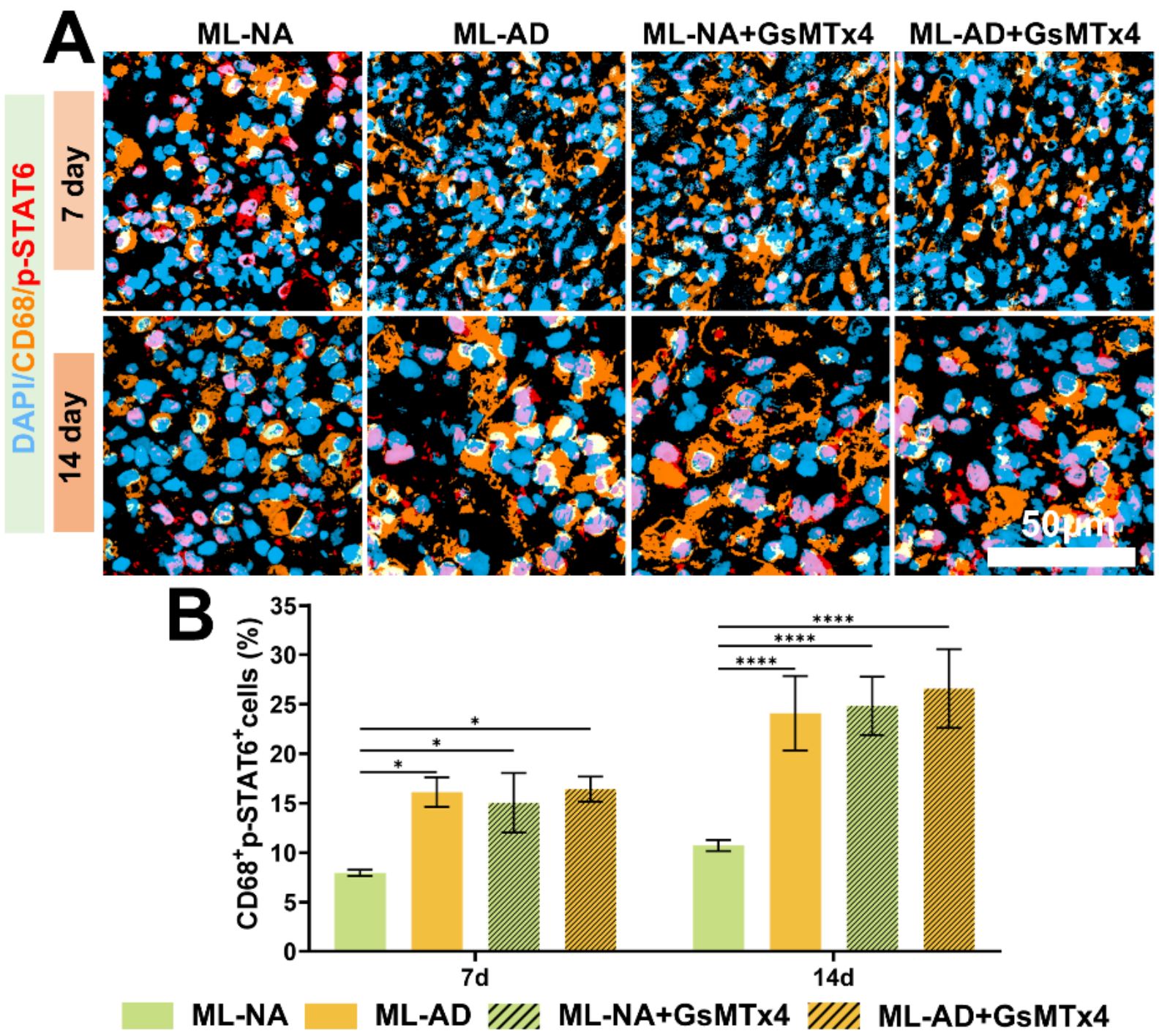

Figure S61. Immunofluorescence staining of the osteogenic region in rats at 7 and 14 days postoperatively. (A) Representative immunofluorescence staining images (CD68 and p-STAT6). (B) Semi-quantitative analysis of $CD68^+$p-$STAT6^+$ double-positive cells. Statistical analyses in this figure were performed via two-way ANOVA. $^*P < 0.05$; $^{****}P < 0.0001$. N = 3.

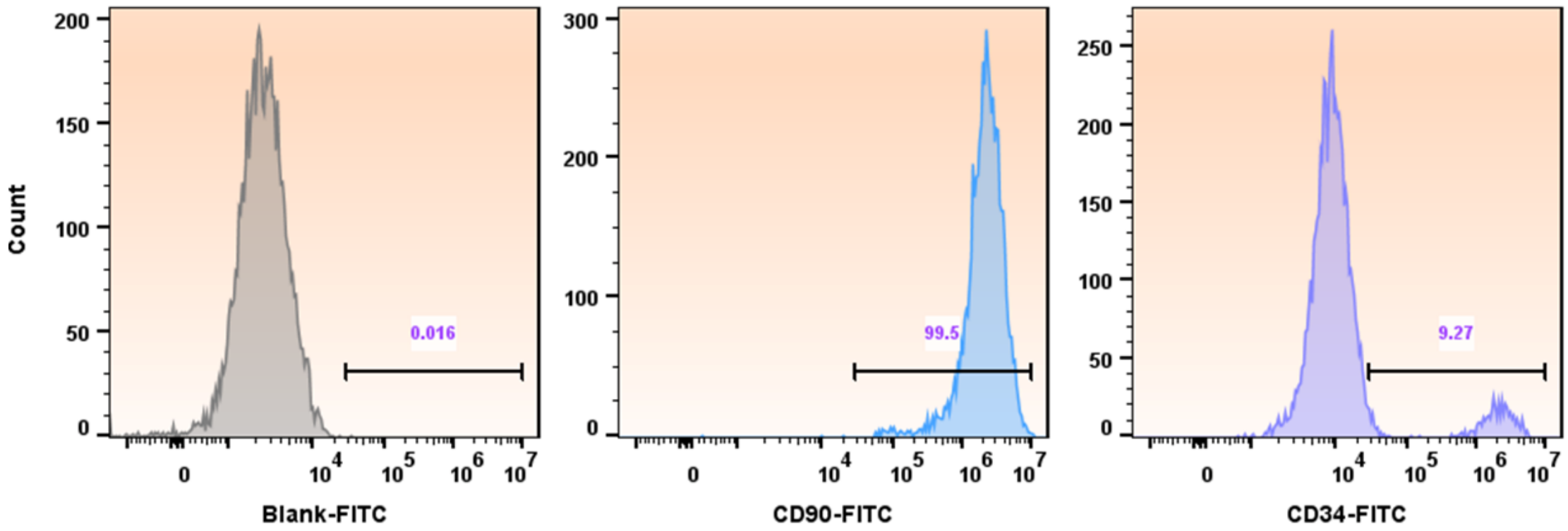

Figure S62. Rat BMSCs were identified by flow cytometry using the markers CD90 and CD34 at passage 3.

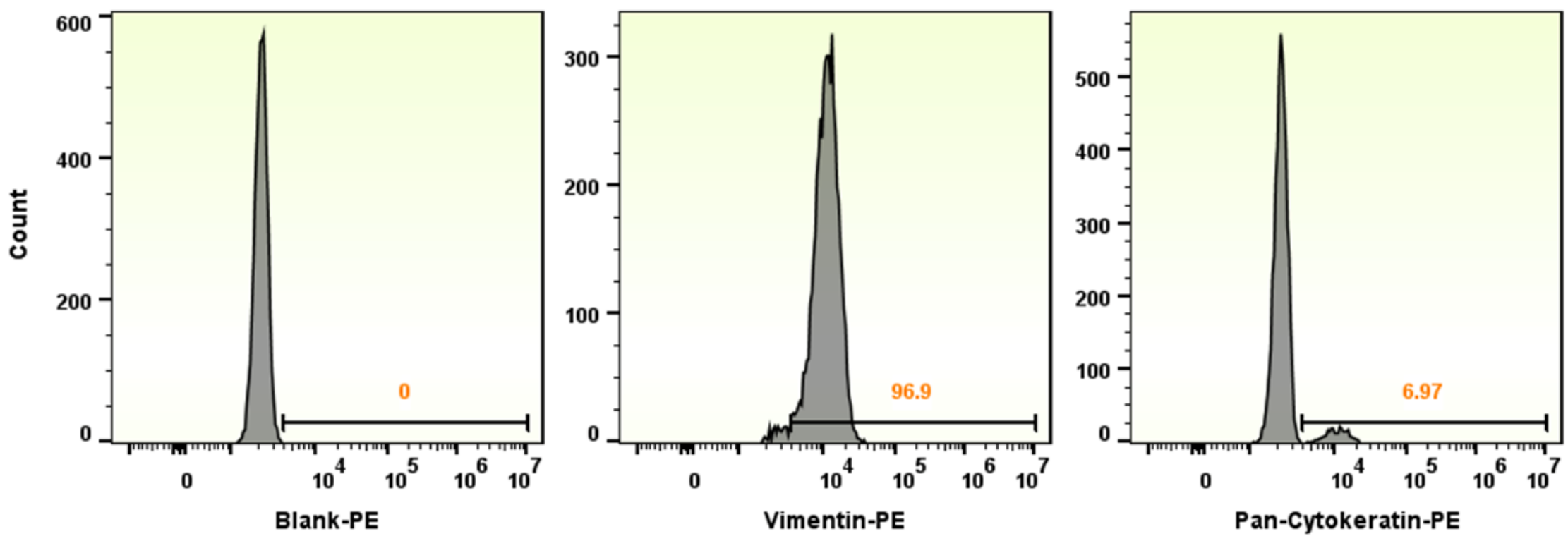

Figure S63. Identification of rat gingival fibroblasts (R-GFs) was performed via flow cytometry with Vimentin and Pan-Cytokeratin markers at the third passage of culture.

Supporting tables

Table S1. The compositions of the barrier layer hydrogels in each group.

Groups (name)	PEGDA (w/v%)	GelMA (w/v%)	MPC (w/v%)
M0	9	1	0
M10	9	1	10
M20	9	1	20
M30	9	1	30

Table S2. The compositions of adhesive layer hydrogels in each group.

Groups (name)	AA (w/v%)	GelMA (w/v%)	Gelatin (w/v%)	DA (w/v%)
D0	30	0.5	10	0
D0.1	30	0.5	10	0.1
D0.5	30	0.5	10	0.5
D1	30	0.5	10	1

Table S3. The compositions of osteogenic layer hydrogels in each group.

Groups (name)	GelMA (w/v%)	nHAp (w/v%)	Laponite (w/v%)
G15	15	0	0
L0	15	6	0
L0.5	15	6	0.5
L1	15	6	1
L2	15	6	2

Table S4. Grouping and naming of animal experiments (rabbits).

Groups	Definition
B	Blank control
CM-BP	Collagen membrane+bone powder (common clinical management without fixation)
CM-BP-P	Collagen membrane+bone powder+pin (common clinical management with fixation)
SS	Single Scaffold (a single-layer construct composed solely of the osteogenic layer)
ML-NA	Multilayer Non-adhesive Scaffold (a three-layer construct consisting of an osteogenic layer, a non-adhesive layer, and a barrier layer; the non-adhesive layer is prepared by prolonging the UV exposure for the adhesive layer), the chemical composition and structure are identical to those of ML-AD.
ML-AD	Multilayer Adhesive Scaffold (a three-layer construct consisting of an osteogenic layer, an adhesive layer, and a barrier layer)

Table S5. Primer sequences for RT-qPCR assay.

Gene	Forward (5'-3')	Reverse (5'-3')
rat-GAPDH	ATGGTGAAGGTCGGTGTGAAC	GAGGTCAATGAAGGGGTCGTT
rat-OSX	GCCTACTTACCCGTCTGACTTTG	CCCTCCAGTTGCCCACTATTG
rat-OCN	CCTCTCTCTGCTCACTCTGCT	CTTACTGCCCTCCTGCTTGG
rat-ALP	CATCGCCTATCAGCTAATGCACA	ATGAGGTCCAGGCCATCCAG
rat-OPN	CCGAGGTGATAGCTTGGCTTA	GATAGCCTCATCGGACTCCTG
rat-Runx-2	CATGGCCGGGAATGATGAG	TGTGAAGACCGTTATGGTCAAAGTG
rat-Col-1	CACTGCAAGAACAGCGTAGC	AAGTTCCGGTGTGACTCGTG

Table S6. The primary antibodies used for immunofluorescence staining in rats.

Primary antibody	Supplier	Catalog No.	Dilution
CD68	servicebio	GB113109	1:500
iNOS	servicebio	GB11119	1:600
CD163	servicebio	GB113751	1:600
VEGF	servicebio	GB15165	1:600
BMP-2	servicebio	GB15252	1:600
CD31	abcam	ab182981	1:300
EMCN	SANTA CRUZ	Sc-65495	1:200
OSX	abcam	ab209484	1:300
OPN	Proteintech	25715-1-AP	1:300
p-NFκB p65	SANTA CRUZ	Sc-166748	1:400
p-STAT6	ZhongHe	ZHS1234	1:500
Piezo1	servicebio	GB150053	1:800

Supporting videos

Video S1.

Testing procedure for bone adhesion tensile strength of the adhesive layer gels (in the presence of SBF and blood).

Video S2.

Demonstration of cranial bone adhesion using the adhesive layer hydrogel (D0.5) in rats under anesthesia.

Video S3.

Demonstration of ML-AD adhesion strength in rabbit tibial plateau defects in the presence of SBF and blood, and peeling off the scaffold adhered to the defect in the presence of blood using a tweezer.

Video S4.

Demonstration of burst test for the scaffold adhered to a rabbit tibial plateau defect in the presence of blood.

Video S5.

Demonstration of peeling off the scaffold adhered to a rabbit tibial plateau defect in the presence of SBF at 7 days post-adhesion using a tweezer.

Video S6.

Display of treadmill running in rabbits with in vivo bone augmentation model during the first 7 days post-modeling surgery

Video S7.

Demonstration of chewing movements during normal diet in rabbits with bone augmentation model post-modeling surgery.

Video S8.

B-mode ultrasound evaluation of scaffold adhesion fixation at defect sites for all groups in rabbit mandibular and tibial bone-augmentation models at 3 days post-surgery.

Video S9.

Demonstration of B-mode ultrasound for evaluating the adhesion fixation of scaffolds at the defect sites in each group at 7 days post-modeling surgery of in vivo mandibular and tibial bone augmentation models in rabbits

Video S10.

Illustration of the implantation process of ML-AD into the mandibular defect sites in rabbit mandibular bone augmentation models.

Video S11.

Presentation of ML-AD scaffold implantation into mandibular defect sites in rabbit mandibular bone augmentation models.

Video S12.

B-mode ultrasound evaluation of scaffold micromotion at defect sites for ML-NA and ML-AD groups in rat tibial bone-augmentation models at 3 and 7 days post-surgery.

References

[1] D. Zhao, X. Wang, B. Cheng, et al., “Degradation-Kinetics-Controllable and Tissue-Regeneration-Matchable Photocross-linked Alginate Hydrogels for Bone Repair,” *ACS Applied Materials & Interfaces* 14 (2022): 21886-21905, https//:doi.org/10.1021/acsami.2c01739.

[2] H. Min, Y. Qi, Y. Zhang, et al., “A Graphdiyne Oxide-Based Iron Sponge with Photothermally Enhanced Tumor-Specific Fenton Chemistry,” *Advanced Materials* 32 (2020): e2000038, https//:doi.org/10.1002/adma.202000038.

[3] L. Yuan, C. Yuan, J. Wei, et al., “Electrospinning/3D printing-integrated porous scaffold guides oral tissue regeneration in beagles,” *Bio-Design and Manufacturing* 7 (2024): 1000-1017, https//:doi.org/10.1007/s42242-024-00311-4.

[4] Y. Li, D. Zhao, Z. Wang, et al., “Minimally invasive bone augmentation through subperiosteal injectable hydroxylapatite/laponite/alginate nanocomposite hydrogels,” *International Journal of Biological Macromolecules* 231 (2023): 123232, https//:doi.org/10.1016/j.ijbiomac.2023.123232.

[5] M. Wu, H. Liu, D. Li, et al., “Smart-Responsive Multifunctional Therapeutic System for Improved Regenerative Microenvironment and Accelerated Bone Regeneration via Mild Photothermal Therapy,” *Advanced Science (Weinheim)* 11 (2024): e2304641, https//:doi.org/10.1002/advs.202304641.
